# Mental health in academia: A national survey on risk and protective factors in the Indonesian context

**DOI:** 10.1371/journal.pone.0358652

**Published:** 2026-09-21

**Authors:** Fitri Ariyanti Abidin, Evi Eliyanah, Fredrick Dermawan Purba, Anissa Lestari Kadiyono, Grace Natasha Sunardy, Syipa Husni Fadilah, Joeri K. Tijdink

**Affiliations:** 1 Department of General, Developmental, and Educational Psychology, Faculty of Psychology, Universitas Padjadjaran, Sumedang, Indonesia; 2 Center for Relationship, Family Life and Parenting Studies, Faculty of Psychology, Universitas Padjadjaran, Sumedang, Indonesia; 3 Department of English, Faculty of Letters, Universitas Negeri Malang, Malang, Indonesia; 4 Akademi Ilmuwan Muda Indonesia (Indonesian Young Academy of Sciences), Jakarta Pusat, Indonesia; 5 Department of Clinical and Health Psychology, Faculty of Psychology, Universitas Padjadjaran, Sumedang, Indonesia; 6 Center for Innovation and Psychological Research, Faculty of Psychology, Universitas Padjadjaran, Sumedang, Indonesia; 7 Department of Social, Organizational, and Cultural Psychology, Faculty of Psychology, Universitas Padjadjaran, Sumedang, Indonesia; 8 Center for Human Capital and Organization Development Studies, Faculty of Psychology, Universitas Padjadjaran, Sumedang, Indonesia; 9 Faculty of Psychology, Universitas Padjadjaran, Sumedang, Indonesia; 10 Department of Ethics, Law and Humanities, Amsterdam UMC, Amsterdam, the Netherlands; Universitas Prima Indonesia, INDONESIA

## Abstract

University academics are central to advancing higher education through teaching, research, and community engagement. However, mental-health challenges in this population have risen in recent years, partially driven by systemic pressures such as hypercompetition, heavy workloads, job insecurity, and escalating expectations for research productivity and publication output. This national survey included 1,380 academics (37.1% men, 62.7% women; M = 40.09 years) from 32 of Indonesia’s 38 provinces, representing both public and private institutions and all academic ranks from lecturer to full professor. The findings showed that nearly half of the participants (40.5%) reported working more than 40 hours per week, exceeding the standard legal working hours in Indonesia, with work often extending into evenings, weekends, and holidays. Most participants reported low/minimal anxiety (63.1%) and moderate stress (63.8%), while depression was mostly low/minimal (43.3%) or mild (36.3%). Female, younger age, academics without children, and those in lower ranks with lower income were the groups most vulnerable to poorer mental health outcomes. Across the risk and protective factors examined, higher publication stress and greater experience of unwanted behavior at the workplace were consistently associated with lower levels of well-being and higher psychological distress (*β* = −.19 to.20 and −.15 to.16, respectively; all *p*s < .001). In contrast, higher personal mastery was consistently associated with better mental-health (*β* = .39), greater work satisfaction (*β* = .33), higher happiness at work (*β* = .37), and lower stress (*β* = −.50), anxiety (*β* = −.40), depression (*β* = −.41), and emotional exhaustion (*β* = −.31) (all *p*s < .001). These findings suggest that academic mental health is shaped by intersecting individual capacities, institutional climates, and global publication pressures. Interventions should therefore move beyond individual-focused strategies and include institutional policies that prevent and address unwanted behavior in the workplace, reduce publication stress, and promote healthier work practices.

## Introduction

Mental health is widely recognized as a crucial determinant of individual functioning, productivity, and institutional performance. Globally, there has been increasing scholarly attention to the mental health of higher education academics, with studies documenting high levels of stress, anxiety, depression, and burnout among faculty members across diverse contexts [1,2]. In many countries, the prevalence of psychological distress among academics exceeds that observed in the general working population [3,4].

A substantial body of research in Europe, North America, and Australia highlights systemic pressures that undermine academic well-being. Specifically, market-oriented managerial reforms and hypercompetition have fostered escalating expectations for research output and publication pressure [5]. These systemic shifts are further compounded by heavy workloads and persistent job insecurity, which have been identified as primary sources of occupational stress for university staff [6]. Previous research further indicates that academics are at heightened risk of emotional exhaustion and burnout, influenced not only by institutional pressures and performance metrics like the H-index, but also by demands arising from their personal and domestic lives [7]. The expansion of performance-based funding and the global university ranking system has intensified competition and bureaucratic demands, often at the expense of academic autonomy, though the extent of these changes varies across countries [8,9]. In Indonesia, for example, recent policy reforms have increasingly tied institutional evaluation to research outputs and accreditation metrics, contributing to greater administrative pressure on academics (e.g., through Merdeka Belajar–Kampus Merdeka and performance-based research grants). This policy is clearly stated, for instance, in the Decree of the Minister of Education, Culture, Research and Technology No. 13/2022 on the Ministry’s Strategic Plan 2020–2024 [10]. It is stated that higher education institutions’ quality is benchmarked through international ranking systems, which has lead academic productivity to be evaluated largely through research outputs and publications. While such expectations may foster academic achievement and institutional development, they may also contribute to emotional exhaustion, reduced job satisfaction [2,11], and increased turnover intentions [12], particularly when accompanied by limited institutional support, mentoring, or resources.

Demographic and occupational characteristics have consistently been identified as important determinants of academics’ mental health. Gender differences are among the most consistently reported findings, with female academics generally experiencing higher levels of stress, anxiety, and burnout than their male counterparts [13,14]. These disparities have been attributed to greater work–family conflict [15], disproportionate caregiving responsibilities [16], and higher psychosocial occupational demands in academic settings [17]. As a result, female academics are more vulnerable to emotional exhaustion and burnout [18]. Gender has also been identified as a significant determinant of anxiety and depression among university teachers [19].

Age has also been associated with mental health outcomes. Younger academics generally report greater psychological distress than older colleagues [19]. Age has likewise been associated with psychosocial work hazards among university lecturers [20]. This increased vulnerability has been linked to early-career challenges, including precarious employment, career uncertainty, and intense publication and promotion pressures [21]. These occupational demands have also been associated with lower job satisfaction among academics [22]. Academic rank further contributes to mental health differences, with academics in junior positions reporting higher levels of anxiety and depression than those in senior positions [23].

Other sociodemographic characteristics have also been linked to academics’ mental health. Marital status may influence psychological well-being through differences in social support and family responsibilities [24]. Balancing family commitments with academic responsibilities may further increase work-related stress and burnout [2].

On the other hand, protective factors have received growing empirical attention. Studies demonstrate that supportive leadership, collegiality, and psychological safety within institutions buffer against mental health deterioration [25–27]. Individual coping strategies, such as adaptive emotion regulation and work–life boundary management, are also linked to resilience and sustained engagement [28].

In Indonesia, attention to mental health in academia is still scarce. The academic profession in Indonesia continues to be perceived as a *calling* for advancing and disseminating knowledge rather than as a financially driven occupation. Lecturers are frequently regarded as engaging in a vocation of service, closely linked to the spiritual notion of *ibadah* (devotion), whereby inner and spiritual satisfaction is expected to take precedence over material gain [29]. Such expectations place academics under pressure to accept working conditions that may be detrimental to their private lives and consequently to their mental health, while still fulfilling their educational responsibilities. Difficult working circumstances are often framed as forms of sacrifice and service, thereby generating spiritual meaning. This idealism, however, confronts the realities of excessive bureaucratization, ambiguous academic standards, and low professionalism in career structures [30].

Under Law of the Republic of Indonesia No. 12/2012 on Higher Education [31], Indonesian lecturers are required to fulfill the *Tri Dharma Perguruan Tinggi,* which comprises teaching, research, and community service. These three pillars function as core indicators of academic productivity and represent the minimum criteria for eligibility for professional allowances and career advancement. Furthermore, Regulation of the Minister of Research, Technology, and Higher Education of the Republic of Indonesia No. 20/2017 [32] stipulates that failure to meet the minimum requirements in teaching, research, and community service may result in the temporary suspension of lecturer professional allowances and professor honorary allowances (Articles 7 and 9).

Since 1999, the Indonesian government has actively encouraged universities to compete in global rankings such as the QS World University Rankings or the The Times Higher Education World University Rankings [33]. In fact, the government has been financially investing in autonomous public universities to advance their global ranking positions [34,35]. While this initiative has stimulated quantity improvement, especially in the academic reputation and publication productivity, it has simultaneously escalated administrative demands through metric-based performance-monitoring systems [36,37] and has threatened research integrity and research quality. Pressure to publish has contributed to unethical research and publication practices [38]. In Indonesia, these practices include submitting to questionable or predatory journals, engaging in cheating, or purchasing publication services via Indonesian e-commerce platforms, as authors and journals face heightened urgency to publish [39]. Moreover, ranking pressures and institutional mandates have translated into expanded teaching loads, research and publishing its results, and financial obligations, particularly as universities pursue greater financial independence [40,41].

Policies designed to elevate international rankings have been operationalized through regulations emphasizing increased research and publication output [42], often without adequate institutional support. Structural barriers to conducting research and publication include demographic disparities and unequal access to digital resources, especially outside Java [43], as well as heavy workloads. Time demands associated with teaching, supervision, and institutional service further constrain research capacity. For female academics, domestic responsibilities exacerbate challenges in balancing professional and personal domains [44]. These accumulated pressures could compromise both academic performance and mental health.

These findings underscore the importance of addressing mental health among academics. Beyond its implications for academics’ own well-being and functioning, poor mental health may also affect their ability to fulfill the multiple roles expected of them within higher education [45]. Academics are responsible not only for teaching, research, and institutional duties, but may also serve as an important source of support for students experiencing psychological difficulties [46]. Mental health difficulties among academics may therefore have broader implications for the academic environment.

In contrast to the extensive body of international research, empirical studies focusing on the mental health of academics in Indonesia are remain limited. Building on insights from global scholarship, the present study seeks to provide the first large-scale, national-level study on the psychological well-being of Indonesian academics. This study addressed four research questions:

1. What is the current state of mental health among academics in Indonesia?
2. Are there differences in mental health status based on gender, academic rank, type of university, age group, financial status, marital status, or geographical location?
3. To what extent are risk factors such as workload, publication pressure, and experiences of unwanted behavior at the workplace associated with mental health outcomes?
4. To what extent are protective factors such as coping strategies associated with mental health in this population?

## Materials and methods

### Design

This study employed a cross-sectional survey design to investigate the mental health of academics in Indonesian higher education institutions.

### Participants

We targeted all academics holding a NIDK registration number in Indonesia’s 4,416 higher education institutions (https://pddikti.kemdiktisaintek.go.id/statistik). Based on https://pddikti.kemdiktisaintek.go.id/statistik, there were 303,067 academics. We calculate minimum sample size requirements using Cochran’s Formula for large populations [47,48], assuming a 95% confidence level, a 3% margin of error, and maximum variability (*p* = .50). The calculation indicated a minimum sample size of 1.067.

Of the 2,675 academics who accessed the survey link, 2,477 met the inclusion criteria, 2,307 provided informed consent and voluntarily participated, and 1,380 completed all seven mental health outcome measures. The final analytic sample exceeded the minimum required sample size of 1,067.

Table 1 shows the demographic characteristics of the 1,380 higher education academics who participated in the study. The majority of participants were female (62.7%). The participants represented a wide range of age groups, predominantly 31–40 years old (46.4%). In terms of marital and parental status, most respondents were married with children (67.0%). Regarding academic rank, the largest group was assistant professors (40.2%). Meanwhile, associate professors and full professors made up 10.5% and 2.5% of the sample, respectively. Most participants were affiliated with private universities (71.7%). Geographically, the majority were based in Java Island (62.4%), with 35.6% located in other regions of Indonesia. In terms of income, the largest proportion of participants (33.2%) reported earning IDR 5,000,000 – IDR 9,999,999 (USD 278–556) followed by 30.3% who earned IDR 3,000,000 – IDR 4,999,999 (USD 167–278). Only 4.2% reported income above USD 1,111. Approximately half of the participants (49.8%) reported having secondary (non-academic) employment, while 48.8% reported not having such employment.

**Table 1:** Demographic characteristics of participants (n = 1,380).

Demographics	n	%
**Gender**		
Male	512	37.10
Female	865	62.68
Missing value	3	.22
**Age Group**		
< 26	9	.65
26-30	193	13.99
31-40	641	46.45
41-50	312	22.61
51-60	171	12.39
61-70	46	3.33
> 71	2	.14
Missing value	6	.43
**Marital and Parental Status**		
Not married without children	253	18.33
Not married with children	56	4.06
Married without children	147	10.65
Married with children	924	66.96
**Academic Rank**		
Teaching staff	215	15.58
Lecturer	428	31.01
Assistant professor	555	40.22
Associate professor	145	10.51
Professor	35	2.54
Missing value	2	.14
**Type of University**		
Public – fully autonomous	157	11.38
Public – semi-autonomous	164	11.88
Public – non-autonomous	61	4.42
Private	989	71.67
Missing value	9	.65
**Geographical Location**		
Java island	861	62.39
Other islands	491	35.58
Missing value	28	2.03
**Income**		
< IDR 3,000,000 (<USD 167)	234	16.96
IDR 3,000,000 – IDR 4,999,999 (USD 167–278)	418	30.29
IDR 5,000,000 – IDR 9,999,999 (USD 278–556)	458	33.19
IDR 10,000,000 – IDR 19,999,999 (USD 556–1,111)	211	15.29
IDR 20,000,000 – IDR 49,999,999 (USD 1,111–2,778)	58	4.20
Missing value	1	.07
**Secondary (Non-Academic) Employment**		
No	673	48.77
Yes	687	49.78
Missing value	20	1.45

### Data collection

Data were collected using an online self-administered questionnaire distributed through multiple recruitment channels. Initially, the survey was disseminated via institutional networks, including the Indonesian Psychology Higher Education Organizer Association (AP2TPI), professional associations (e.g., the Indonesian Campus Workers’ Union), and various WhatsApp groups for academics (e.g., lecturer groups across multiple universities). Personalized email invitations were also sent to 5,827 lecturers.

After one month of recruitment, an evaluation showed that the initial strategy had not yielded the targeted sample size. Formal invitations were therefore distributed through Regional Higher Education Service Institutions (LLDIKTI; formerly known as KOPERTIS). As the targeted sample size had still not been reached after another month, the survey was subsequently promoted through Dosen Update (https://www.instagram.com/dosenupdate/), an Instagram account followed by approximately 96,200 Indonesian academics and sharing information relevant to higher education and academic life.

Data collection was conducted between 27 July and 31 October 2025. The survey was administered in Bahasa Indonesia, the national language and the primary language of instruction in Indonesian higher education. Given that all participants were university academics, no language-related barriers to survey completion were anticipated. Participation was voluntary, and electronic written informed consent was obtained online prior to survey completion. Anonymity and confidentiality were assured. To respect participants’ autonomy and preserve anonymity, individuals who did not complete the survey were not contacted for follow-up or reminder purposes.

### Measurements

#### Demographic variables.

Information was collected on gender, age, marital and parental status, academic rank, type of university (public vs. private), and location of university (Java vs outside Java), income, and secondary (non-academic) employment.

#### Mental health indicators.

Well-being was measured using the Indonesia version of the Mental Health Continuum–Short Form (MHC-SF) [49,50], a 14-item measure of emotional, psychological, and social well-being. The scale comprises three subscales: emotional well-being (3 items; e.g., “happy”), psychological well-being (6 items; e.g., “that you liked most parts of your personality”), and social well-being (5 items; e.g., “that you had something important to contribute to society”). Participants reported the frequency of their experiences over the past month on a 6-point scale ranging from 0 (never) to 5 (every day). The Indonesian version has demonstrated adequate psychometric properties [50]. Previous validation findings provided support for the original three-factor structure, with acceptable model fit indices (CFI = .97, IFI = .97, GFI = .99, RMSEA = .05), and internal consistency coefficients of.81,.81, and.73 for the emotional, psychological, and social well-being subscales, respectively [50]. In the present study, internal consistency estimates were α = .90 for emotional well-being, α = .87 for psychological well-being, α = .82 for social well-being, and α = .93 for the overall scale.

Work satisfaction was measured using the Satisfaction With Work Scale (SWWS) [51], a work-context version of the Satisfaction With Life Scale (SWLS) [52–54]. It is a 5-item scale measuring cognitive evaluations of one’s work life satisfaction (e.g., “In most ways my life is close to my ideal”). Responses were rated on a 7-point Likert scale ranging from 1 (strongly disagree) to 7 (strongly agree). The scale was translated and adapted into Indonesian through a forward–backward translation procedure, followed by expert review and cognitive interviews to ensure conceptual and cultural equivalence. In the present study, CFA supported the expected one-factor structure (CFI = .99, TLI = .97, GFI = .98, RMSEA = .11, SRMR = .02). The scale also demonstrated good internal consistency (α = .90).

Subjective happiness was measured using an adapted work-context version of the Subjective Happiness Scale (SHS) [55], a 4-item measure of global subjective happiness at work. The scale was translated and adapted into Indonesian through a forward–backward translation procedure, followed by expert review and cognitive interviews to ensure conceptual and cultural equivalence. Items were adapted to the work context while preserving the original item structure and response format. An example item is “In general, I consider myself very happy at work.” Responses were rated on a 7-point Likert scale ranging from 1 (strongly disagree) to 7 (strongly agree), with negatively worded items (items 2 and 4) reverse-coded. In the present study, CFA supported the expected one-factor structure (CFI = .99, TLI = .99, GFI = .99, RMSEA = .04, SRMR = .01). The scale also demonstrated good internal consistency (α = .83).

Subjective stress levels were measured using the Indonesian version of Perceived Stress Scale (PSS) [56–58], a 10-item measure perceived stress during the past month (e.g., “How often have you been upset because of something that happened unexpectedly?”). Responses were rated on a 5-point scale ranging from 0 (never) to 4 (very often), with positively worded items (items 4, 5, 7, and 8) reverse-coded. Stress levels were classified using established cutoff scores [59–62], categorizing scores as low (0–12), moderate (13–27), and high stress (28–40). The Indonesian version has demonstrated good psychometric properties. CFA supported the original two-factor structure, with acceptable model fit indices (CFI = .99, TLI = .99, RMSEA = .08, and SRMR = .06) [57]. The scale also demonstrated good internal consistency in the present study (α = .82).

Anxiety severity was assessed using the Indonesian version of the Generalized Anxiety Disorder Scale (GAD-7) [63,64], a 7-item measures of anxiety symptoms experienced during the past two weeks (e.g., “Feeling nervous, anxious, or on edge”). Responses were rated on a 4-point scale from 0 (not at all) to 3 (nearly every day). Anxiety levels were interpreted according to established cutoff scores [63]: minimal (0–4), mild (5–9), moderate (10–14), and severe anxiety (15–21). The Indonesian version demonstrated good predictive validity in an Indonesian sample, with an AUC of.86, indicating good discrimination between individuals with and without anxiety symptoms [64]. In the present study, CFA supported the expected one-factor structure (CFI = .98, TLI = .96, GFI = .97, RMSEA = .08, SRMR = .03). The scale also demonstrated good internal consistency (α = .91).

Depressive symptoms were measured using the Indonesian version of the Quick Inventory of Depressive Symptomatology–Self Report (QIDS-SR) [65,66], 16-item measure assessing nine symptom domains of major depressive disorder (e.g., “Feels intensely sad virtually all the time.”). The Indonesian version was adapted from the Inventory of Depressive Symptomatology–Self Report (IDS-SR) [66], selecting the 16 items consistent with the QIDS-SR structure. Responses were rated on a 4-point scale (0–3). Following Rush et al. [67], depression scores were classified as no symptoms (0–5), mild (6–10), moderate (11–15), severe (16–20), and very severe depression (21–27). In the present study, CFA supported one-factor structure (CFI = .92, TLI = .90, GFI = .95, RMSEA = .05, SRMR = .04. The scale also demonstrated good internal consistency (α = .80).

Emotional Exhaustion was assessed using the Maslach Burnout Inventory – Emotional Exhaustion subscale (MBI) [68], a 7-items measure of work-related emotional exhaustion (e.g., “I feel emotionally drained by my work”). The scale was translated and adapted into Indonesian through a forward–backward translation procedure, followed by expert review and cognitive interviews. Participants rated how often they experienced each symptom on a 7-point scale ranging from 0 (never) to 6 (every day). In the present study, CFA supported the expected one-factor structure (CFI = .98, TLI = .97, GFI = .98, RMSEA = .08, SRMR = .03). The scale also demonstrated good internal consistency (α = .92).

#### Risk and protective factors.

In addition to demographic characteristics, risk and protective factors were assessed using the following instruments:

Publication stress was measured using the Publication Pressure Questionnaire–Revised (PPQ-r) [69], specifically the 6-item publication stress subscale assessing perceived pressure related to academic publishing (e.g., “I feel forced to spend time on my publications outside office hours”). The scale was translated and adapted into Indonesian through a forward–backward translation procedure, followed by expert review and cognitive interviews. Responses were rated on a 5-point Likert scale ranging from 1 (totally disagree) to 5 (totally agree), with positively worded items (items 5 and 6) reverse-coded. In the present study, CFA supported the expected one-factor structure (CFI = .98, TLI = .96, GFI = .98, RMSEA = .08, SRMR = .03). The scale also demonstrated good internal consistency (α = .84).

Workload was assessed using three questions designed to capture the intensity and distribution of academic work demands. Participants were asked to report the average number of hours they worked per week, with response options representing a range from 1–8 hours to more than 56 hours per week. A second question assessed how often they worked on weekends or public holidays, with response categories spanning from almost never to most weekends or holidays. A third question measured the frequency of working overtime or at night, with responses ranging from almost never to most afternoons or evenings of the week. These questions were developed to reflect varying patterns of workload typically experienced within academic roles.

Experiences of unwanted behavior at the workplace were assessed using questions that examined whether participants had experienced any form of unwanted behavior or violence in their workplace. Participants who reported such incidents were asked to provide further details regarding (1) the type of violence experienced, by selecting one or more categories including verbal violence, bullying, threats or intimidation, sexual intimidation, discrimination or racism, and physical violence; an “other” option was also provided, with participants asked to briefly describe experiences not covered by the listed categories; (2) the individuals involved in the incident; and (3) the participant’s role in the incident.

Perceived control over life circumstances was measured using the Pearlin Mastery Scale [70], a 7-item scale measure of perceived personal coping and mastery over life circumstances (e.g., “I have little control over the things that happen to me”). The scale was translated and adapted into Indonesian through a forward–backward translation procedure, followed by expert review and cognitive interviews. Responses were rated on a 5-point Likert scale ranging from 1 (strongly disagree) to 5 (strongly agree), with negatively worded items (items 1, 2, 3, 5, and 7) reverse-coded. In the present study, CFA supported the expected one-factor structure (CFI = .98, TLI = .96, GFI = .98, RMSEA = .06, SRMR = .03). The scale also demonstrated acceptable internal consistency (α = .78).

Relational coping was measured using two items that assessed the availability and quality of interpersonal support. The first item evaluated the number of significant others through categorical response options ranging from 0–1, 2–5, 6–10, 11–15, 16–20, and more than 20. The second item measured quality time with significant others, using three response categories: No, Sometimes, and Yes.

Self-care coping was assessed using two questions capturing personal well-being activities. The first question measured time to do hobbies, with three response options (No, Sometimes, Yes). The second question assessed time to exercise, also using three categories (No, Sometimes, Yes/Yes, regularly).

See S1 Table for a summary of all instruments.

### Data analysis

Of the 2,675 academics who accessed the survey link, 2,477 met the inclusion criteria, 2,307 provided informed consent and voluntarily participated, and 1,380 completed all seven mental health outcome measures, corresponding to a completion rate of 51.6%. An attrition analysis was conducted by comparing completers and non-completers across demographic characteristics using Pearson’s chi-square tests. Significant differences were observed for institution type, χ²(3) = 9.04, p = .029, and geographic location, χ²(1) = 5.06, *p* = .025. No significant differences were found for gender, age group, marital and parental status, academic rank, income, or secondary (non-academic) employment category (all *p*s > .05). Detailed attrition analysis results are provided in S2 Table.

Descriptive statistics were employed to summarize demographic characteristics and key mental health indicators. Group differences across gender, age, academic rank, and university type were analyzed using independent-sample t-tests and one-way ANOVAs. Multiple regression analyses using the Ordinary Least Squares (OLS) estimation method were subsequently performed to examine the correlation of identified risk factors (e.g., workload, publication pressure, experience of unwanted behavior at the workplace, and demographic characteristics) and protective factors (e.g., coping strategies) to mental health outcomes.

Regression assumptions, including normality of residuals, multicollinearity, and homoscedasticity, were evaluated prior to the multiple regression analyses. Visual inspection of the histograms and normal Q–Q plots indicated that the residuals were mostly normally distributed, with only slight departures from normality. Multicollinearity was examined using tolerance and variance inflation factor (VIF) statistics, with tolerance values ranging from.411 to.960 and VIF values ranging from 1.041 to 2.432, indicating no problematic multicollinearity. Homoscedasticity was assessed using scatterplots of standardized residuals and the Breusch–Pagan test. As some evidence of heteroscedasticity was observed, all regression models were estimated using heteroscedasticity-consistent (HC3) robust standard errors.

Separate multiple regression models were estimated for each of the seven mental health outcomes (well-being, work satisfaction, happiness at work, stress, anxiety, depression, emotional exhaustion). Each regression model included 18 predictors representing demographic, occupational, relational, and psychological domains. All predictors were entered simultaneously into each model. Nominal demographic variables with more than two categories were recoded into dichotomous categories and entered them as binary predictors (e.g., married vs. not married; private vs. public university). Other demographic variables with naturally ordered categories (e.g., academic rank and income level) were entered using their ordinal coding. To control for the inflation of Type I error due to simultaneous hypothesis testing within each regression model, Bonferroni corrections were applied to the regression coefficient p-values (adjusted significance threshold: α = .05/18 ≈ .0028). Interpretation of statistical significance was based on these corrected thresholds. All statistical analyses were conducted using IBM SPSS Statistics (version 29.0) and R (version 4.6.1), with a two-tailed significance level set at *p* < .05.

For experiences of unwanted behavior in the workplace, responses provided under the “other” option were further examined to determine whether they could be incorporated into the predefined categories. These responses were reviewed and coded by the research team. Where applicable, responses were recategorized into the existing categories, while responses that could not be classified within them were grouped into new categories based on similarities in their content.

We preregistered our study on the Open Science Framework. The preregistration details can be publicly at https://osf.io/zq6ef. Ethical approval for the study was granted by the Ethical Committee of Universitas Padjadjaran under approval number [586/UN6.KEP/EC/2025].

## Results

### The current state of mental health among academics in Indonesia

Descriptive statistics indicated that Indonesian higher-education academics reported a mean well-being score of 62.73 (SD = 13.86), a life satisfaction score of 24.47 (SD = 6.68), and a happiness-at-work score of 20.14 (SD = 5.55). Indicators of psychological distress showed mean scores of 14.98 (SD = 6.43) for stress, 3.99 (SD = 4.23) for anxiety, and 6.99 (SD = 4.46) for depression. Emotional exhaustion was characterized by a mean score of 13.08 (SD = 10.73).

**Fig 1:**
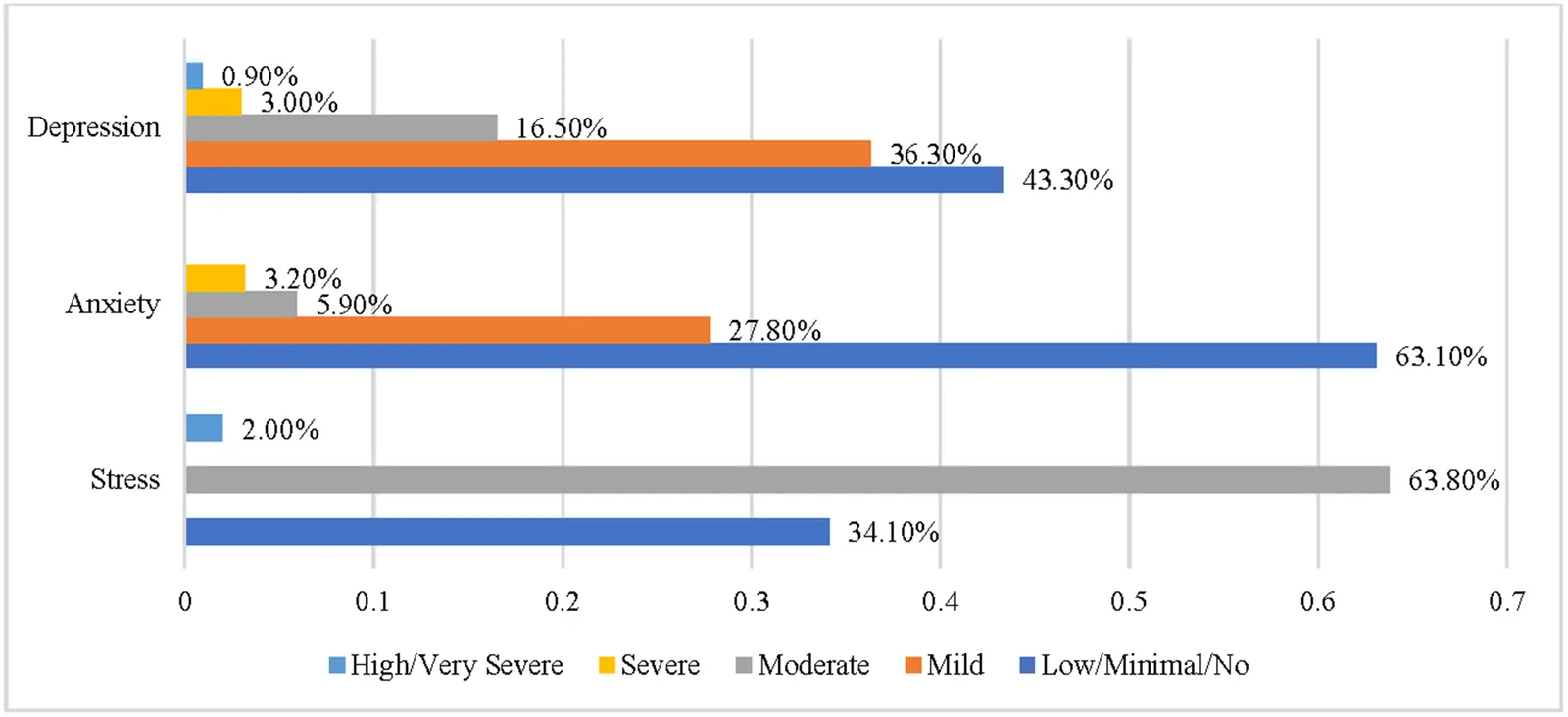
Prevalence of stress, anxiety, and depression.

Moderate stress was the most frequently reported category, while minimal to mild symptoms constituted the majority of anxiety and depression classifications. Notably, only a small proportion of respondents (approximately 2–3%) fell within the severe or very severe ranges across all three domains, indicating that high levels of psychological distress were relatively uncommon in participants (Fig 1).

### The differences in mental health status based on demographic characteristics

Table 2 presents differences in mental health outcomes across sociodemographic and occupational characteristics. Female academics reported lower mean levels of well-being than their male counterparts and higher mean levels of stress, anxiety, depression, and emotional exhaustion. Age-related differences were also observed. Academics aged 26–40 reported the lowest mean levels of well-being, job satisfaction, and happiness, alongside the highest mean levels of stress, anxiety, depression, and emotional exhaustion. Differences by marital and parental status were evident. Academics without children reported lower mean scores on well-being, work satisfaction, and happiness, and higher mean levels of stress, anxiety, and depression compared with those who had children. Marked variations were also found across academic ranks. Teaching staff and lecturers reported the lowest mean levels of well-being, work satisfaction, and happiness, together with the highest mean levels of stress, anxiety, and emotional exhaustion.

**Table 2:** Differences in mental health status based on gender, academic rank, type of university, age group, financial status, marital status, or geographical location.

	Well-being (MHC-SF [49])	Work Satisfaction (SWWS [51])	Happiness at Work (SHS [55])	Stress (PSS [56])	Anxiety (GAD-7 [63])	Depression (QIDS-SR [65])	Emotional Exhaustion (MBI [68])
	Mean (SD)						
**Gender**							
Male	64.13 (13.94)	24.94 (6.84)	20.40 (5.54)	14.01 (6.25)	3.38 (3.95)	6.33 (4.31)	11.79 (10.24)
Female	**61.92 (13.74)**	24.21 (6.56)	19.99 (5.55)	**15.54 (6.45)**	**4.35 (4.35)**	**7.37 (4.51)**	**13.84 (10.94)**
**Age Group**							
< 26	**61.33 (10.44)**	**23.00 (4.85)**	19.22 (4.38)	17.11 (5.67)	6.22 (5.26)	9.11 (5.82)	18.33 (11.14)
26-30	**57.91 (13.17)**	**22.90 (6.68)**	**18.37 (4.99)**	**16.60 (5.38)**	**4.89 (3.94)**	**8.25 (4.33)**	**16.10 (10.42)**
31-40	**61.26 (13.74)**	**23.95 (6.57)**	**19.69 (5.53)**	**16.03 (6.16)**	**4.53 (4.34)**	**7.71 (4.53)**	**14.61 (11.58)**
41-50	64.54 (13.57)	24.42 (6.95)	20.48 (5.72)	14.20 (6.68)	3.75 (4.55)	6.30 (4.43)	11.58 (10.12)
51-60	67.94 (13.57)	27.08 (6.07)	22.40 (5.23)	12.02 (6.52)	2.14 (2.83)	4.69 (3.73)	8.35 (8.77)
61-70	71.87 (9.29)	28.98 (4.10)	23.35 (4.06)	9.76 (4.85)	1.17 (1.97)	4.41 (2.56)	5.02 (4.66)
> 71	73.00 (9.90)	28.00 (2.83)	22.50 (7.78)	7.00 (9.90)	1.00 (1.41)	4.50 (2.12)	5.50 (4.66)
**Marital and Parental Status**							
Not married, without children	**58.86 (13.58)**	**22.81 (6.71)**	**18.53 (5.39)**	**16.54 (6.03)**	**5.02 (4.21)**	**8.34 (4.71)**	**15.79 (10.91)**
Not married, with children	65.11 (12.65)	25.79 (5.85)	21.54 (4.38)	13.30 (6.14)	2.66 (3.14)	5.93 (4.12)	9.43 (8.03)
Married, without children	**59.67 (14.09)**	**22.88 (6.62)**	**18.96 (5.66)**	**16.16 (6.50)**	**4.90 (4.94)**	**7.89 (4.48)**	**16.13 (11.27)**
Married, with children	64.13 (13.70)	25.10 (6.61)	20.69 (5.52)	14.47 (6.44)	3.65 (4.10)	6.54 (4.31)	12.08 (10.5)
**Academic rank**							
Teaching staff	**61.00 (13.28)**	**23.20 (6.79)**	**19.40 (5.47)**	**15.46 (5.94)**	**4.36 (4.08)**	**7.64 (4.60)**	**14.26 (10.85)**
Lecturer	**60.55 (13.85)**	**23.07 (6.79)**	**19.12 (5.63)**	**15.83 (6.29)**	**4.53 (4.58)**	**7.73 (4.76)**	**14.70 (11.10)**
Assistant professor	**63.13 (13.74)**	**25.02 (6.29)**	**20.38 (5.35)**	**15.01 (6.42)**	**3.89 (4.11)**	6.77 (4.25)	**12.52 (10.60)**
Associate professor	67.37 (13.94)	27.19 (6.40)	22.48 (5.36)	12.81 (6.76)	2.70 (3.63)	5.32 (3.70)	10.43 (9.60)
Professor	74.03 (7.58)	29.40 (4.97)	23.74 (4.24)	10.11 (5.86)	2.26 (3.50)	4.32 (2.60)	6.31 (5.52)
**Type of University**							
Public – fully autonomous	61.08 (14.65)	23.83 (7.32)	19.20 (5.81)	15.07 (7.00)	4.41 (4.43)	7.17 (4.95)	14.29 (11.07)
Public – semi-autonomous	62.44 (13.15)	24.95 (6.91)	20.24 (5.70)	15.23 (6.37)	3.84 (4.06)	7.10 (4.20)	12.96 (10.18)
Public – non-autonomous	60.85 (12.70)	24.33 (5.86)	19.26 (5.57)	15.85 (6.19)	3.67 (3.63)	7.30 (4.46)	16.02 (10.16)
Private	63.19 (13.89)	24.54 (6.58)	20.34 (5.55)	14.85 (6.63)	3.96 (4.25)	6.89 (4.42)	12.73 (10.75)
**Geographical Location**							
Java island	62.73 (13.96)	24.55 (6.70)	19.99 (5.63)	15.10 (6.45)	4.10 (4.25)	6.92 (4.43)	**13.76 (11.13)**
Other island	62.84 (13.72)	24.34 (6.69)	20.37 (5.44)	14.77 (6.40)	3.79 (4.15)	7.05 (4.54)	11.96 (10.02)
**Income**							
< IDR 3,000,000 (USD 167)	**61.30 (14.22)**	**22.97 (6.93)**	**19.18 (5.65)**	**15.75 (5.76)**	**4.46 (4.39)**	**7.49 (4.53)**	**13.80 (11.75)**
IDR 3,000,000–IDR 4,999,999 (USD 167–278)	**61.13 (13.81)**	**23.81 (6.58)**	**19.37 (5.47)**	**15.50 (6.33)**	**4.18 (4.17)**	**7.60 (4.50)**	**13.48 (10.36)**
IDR 5,000,000–IDR 9,999,999 (USD 278–556)	62.42 (13.82)	24.57 (6.53)	**20.36 (5.36)**	**15.63 (6.50)**	**4.18 (4.32)**	7.14 (4.42)	**13.94 (11.01)**
IDR 10,000,000–IDR 19,999,999 (USD 556–1,111)	66.16 (12.88)	26.23 (6.31)	21.62 (5.56)	12.73 (6.43)	3.04 (3.86)	5.54 (4.11)	10.78 (9.56)
IDR 20,000,000–IDR 49,999,999 (USD 1,111–2,778)	69.83 (12.42)	27.93 (6.31)	22.50 (5.25)	11.34 (6.13)	2.84 (4.00)	4.67 (3.59)	9.09 (8.91)
**Secondary (Non-Academic) Employment**							
No	**60.81 (14.19)**	**23.92 (6.67)**	**19.49 (5.72)**	**15.70 (6.40)**	**4.36 (4.32)**	**7.48 (4.45)**	**13.97 (10.86)**
Yes	64.57 (13.42)	25.00 (6.65)	20.76 (5.34)	14.28 (6.37)	3.64 (4.15)	6.48 (4.44)	12.15 (10.54)

Bold values represent statistically significant differences (p < .05). Lower scores on positive indicators and higher scores on negative indicators denote groups that exhibit greater psychological vulnerability. The complete statistical results are provided in the S3 Table. See S1 Table for instrument details.

Finally, income level demonstrated a clear gradient pattern. Academics earning below IDR 3 million per month reported the lowest mean levels of well-being and the highest levels of stress, anxiety, and depression, while those earning above IDR 20 million reported the most favorable mental health indicators. Differences were also evident by employment arrangement. Academics relying solely on academic employment reported lower well-being, work satisfaction, and happiness, and higher stress, anxiety, depression, and emotional exhaustion than those with secondary sources of income.

### Descriptive of risk and protective factors on mental health outcomes

**Fig 2:**
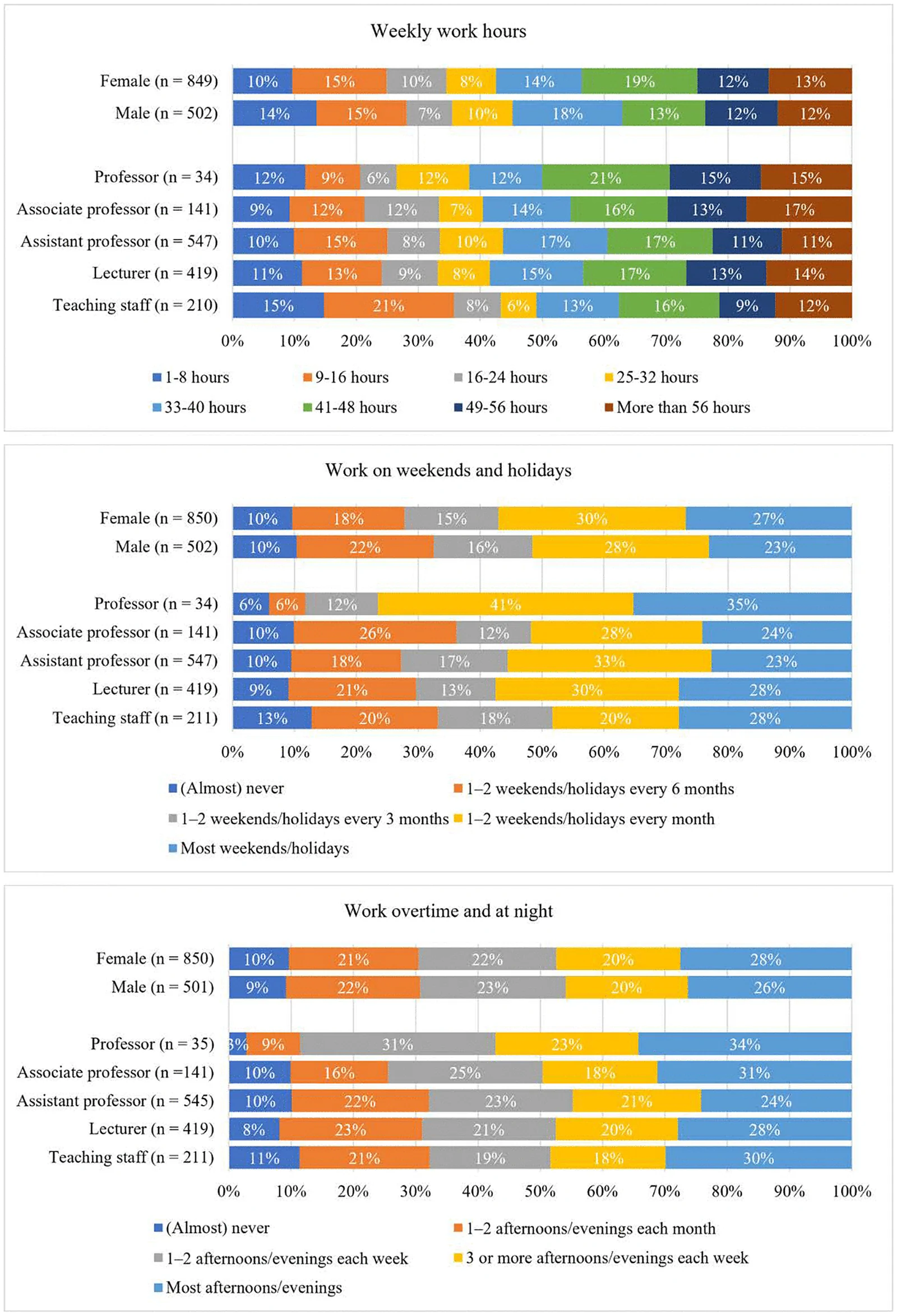
Distribution of workload indicators among Indonesian academics across gender and academic rank. *n* values represent the number of participants with non-missing data for the variable shown in this figure.

Regarding risk factors, publication pressure showed a mean score of 17.78 (SD = 5.45). In contrast, mastery as a protective factor demonstrated a mean score of 24.85 (SD = 4.83). Fig 2 illustrates substantial workload demands among Indonesian academics, characterized by long weekly working hours and frequent work outside regular working days. Across gender and academic ranks, a large proportion of respondents reported working more than 40 hours per week, with a notable share exceeding 56 hours. Work during weekends and holidays was common, with many academics reporting engagement at least monthly or more frequently. Overtime and night work were also prevalent, with most respondents indicating weekly or near-daily work outside standard hours. These patterns were observed across all academic ranks, with a tendency toward higher workload demands among more senior positions.

**Fig 3:**
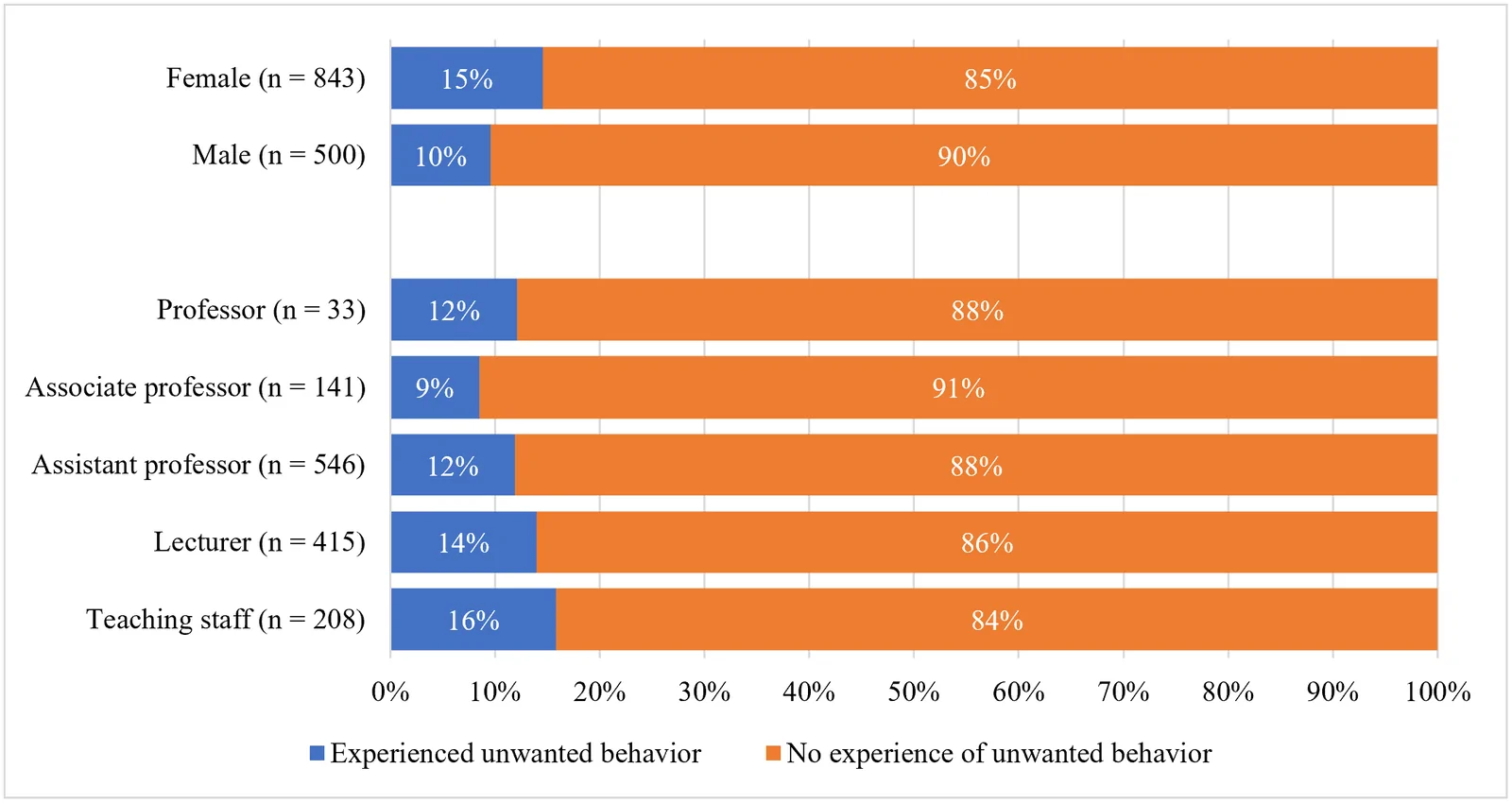
Distribution of experiences of unwanted behavior at the workplace among Indonesian academics across gender and academic rank. *n* values represent the number of participants with non-missing data for the variable shown in this figure.

Fig 3 presents reports of experiences of unwanted behavior at the workplace among Indonesian academics across gender and academic ranks. Overall, the majority of respondents indicated not experiencing unwanted behavior or violence in the workplace, with proportions ranging from approximately 84% to 91% across groups. Nevertheless, a non-negligible proportion reported experiencing unwanted behavior or violence, ranging from about 9% to 16%. Reports of experiencing violence were observed across both genders, with a slightly higher proportion among female academics compared with males. Across academic ranks, experiences of unwanted behavior or violence were present at all levels, with teaching staff and lecturers reporting relatively higher proportions compared with professors and associate professors.

Table 3 shows that participants reported the types of unwanted behavior, with the most frequently reported forms being verbal violence, including insults, harsh criticism, and humiliation; threats and intimidation intended to elicit fear or compliance; and bullying, characterized by repeated targeted mistreatment or exclusion. Other reported behaviors included discrimination and racism, physical violence, and sexual intimidation. Additional forms emerged from responses provided under the “other” category, including professional injustice, character defamation and slander, psychological harassment or emotional abuse, and political or organizational violence.

**Table 3:** Types of unwanted behavior and violence experienced in the workplace reported by participants.

Category	n	Description/ Examples	Illustrative Responses
Verbal violence	106	Insults, harsh criticism, humiliation, or negative labeling from colleagues or superiors.	“Constantly being blamed,” “Receiving sarcastic remarks,” “Public humiliation.”
Bullying	39	Repeated, targeted mistreatment or exclusion aimed at undermining a colleague.	“Being ignored and isolated,” “Colleagues obstructing my administrative work,” “Unequal task distribution.”
Threats and intimidation	57	Verbal or nonverbal actions intended to create fear, submission, or compliance.	“Verbal threats and intimidation at work,” “Power abuse by superiors.”
Sexual intimidation	5	Unwanted sexual comments, gestures, or advances.	“Digital harassment,” “Inappropriate personal remarks.”
Discrimination and racism	39	Unfair treatment based on gender, position, academic background, or personal characteristics.	“Unfair workload and promotion practices,” “Being undervalued due to professional background.”
Physical violence	10	Physical aggression or harm.	“Being hit with a stick,” “Physical assault by a colleague.”
Professional injustice (additional category)	12	Abuse of institutional power, maladministration, or career obstruction.	“Career restriction,” “Unilateral dismissal,” “Unfair SKP evaluation,” “Subjective leadership decisions.”
Character defamation and slander (additional category)	5	Spreading false or tendentious information to damage reputation.	“Cruel defamation,” “Slander among colleagues,” “Spreading false rumors.”
Psychological harassment/ emotional abuse (additional category)	2	Acts that affect emotional well-being or mood stability.	“Loss of control due to others,” “Feeling constantly wrong,” “Disturbed mood due to external issues.”
Political or organizational violence (additional category)	1	Power manipulation or exclusion within institutional or political contexts.	“Political violence,” “Favoritism and group-based injustice.”

Table 4 summarizes the individuals involved in unwanted behaviors or violent incidents and the participants’ roles. Most incidents involved colleagues within the department, program, faculty, or university, followed by supervisors and individuals encountered in participants’ personal lives. Other involved parties included administrative or support staff and students, while some participants chose not to specify. In terms of roles, participants were predominantly victims, with a few reporting being perpetrators, witnesses, or occupying other roles.

**Table 4:** The individuals involved and participant’s role in the unwanted behavior or violent incident.

Individual Involved	Frequency
Supervisor	76
Colleagues in department/program/faculty/university	89
Administrative/ support staff	16
Students	9
Experienced in personal life	34
Prefer not to say	29
**Participant Role**	**Frequency**
Victim/ I was the victim	141
Perpetrator/ I was the perpetrator	4
Witness	13
Other	12
Prefer not to answer	25

**Fig 4:**
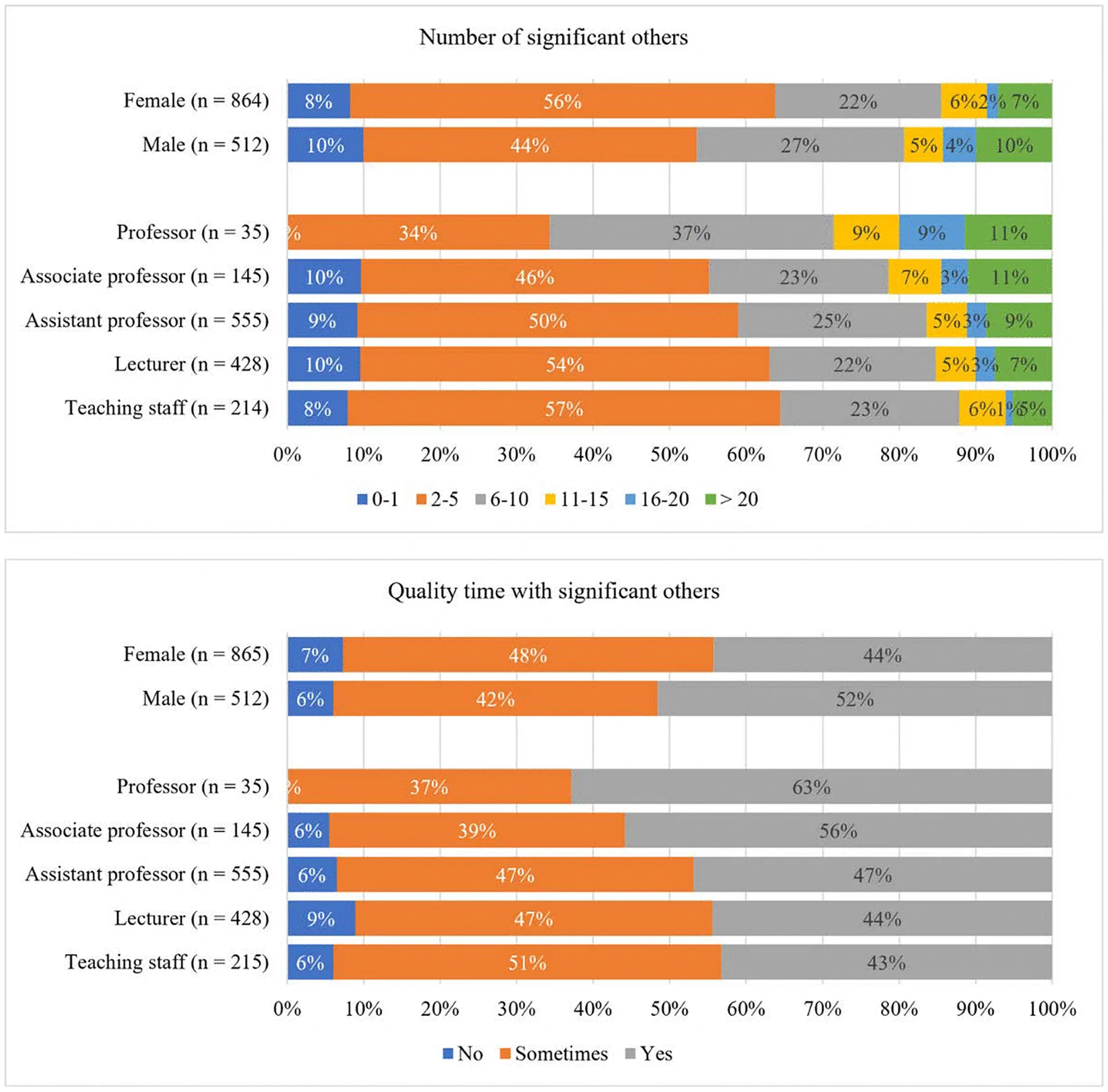
Distribution of relational indicators among Indonesian academics across gender and academic rank. *n* values represent the number of participants with non-missing data for the variable shown in this figure.

Fig 4 depicts relational characteristics among Indonesian academics across gender and academic ranks. Across all groups, the majority of respondents reported having a small to moderate number of significant others, most commonly in the range of 2–5 individuals, followed by 6–10 individuals. Only a small proportion reported having very few (0–1) or a large number of significant others. This overall pattern was consistent across gender and academic positions. Regarding quality time with significant others, most academics indicated that they were able to spend quality time at least sometimes, while a substantial proportion reported doing so regularly. Reports of having no quality time were relatively uncommon across groups. However, consistent quality time appeared less prevalent among teaching staff and lecturers compared with more senior academic ranks.

**Fig 5:**
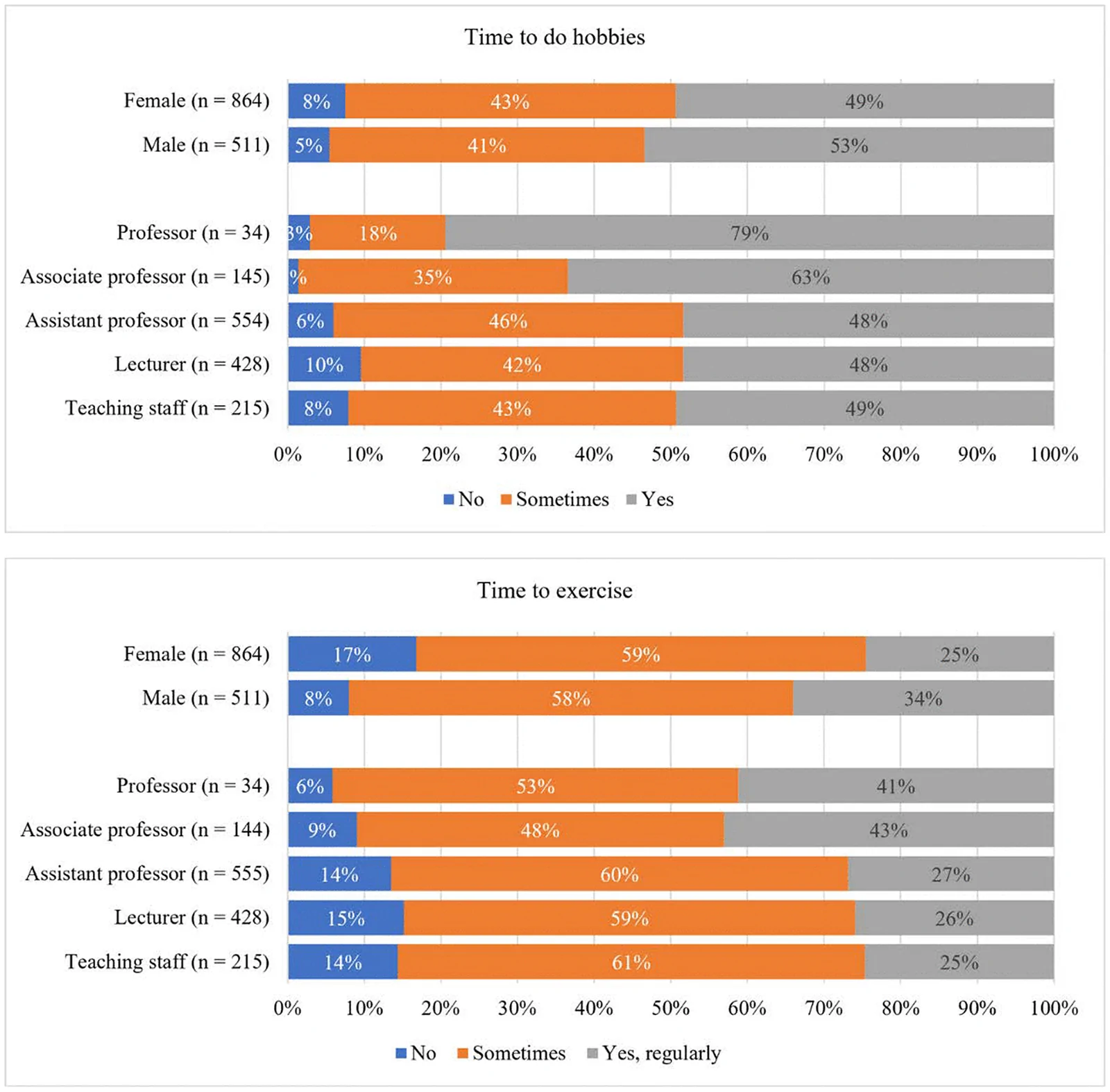
Distribution of self-care patterns among Indonesian academics across gender and academic rank. *n* values represent the number of participants with non-missing data for the variable shown in this figure.

Fig 5 presents self-care patterns among Indonesian academics across gender and academic ranks, focusing on time for hobbies and exercise. Overall, most academics reported having time for hobbies or exercise only sometimes, while fewer indicated having regular opportunities for these activities. With regard to time for hobbies, a substantial proportion of respondents across ranks reported having such time occasionally, whereas regular engagement was more commonly reported among senior academics, particularly professors and associate professors. Reports of having no time for hobbies were relatively uncommon but appeared more frequently among early-career academics, including lecturers and teaching staff. A similar pattern was observed for time to exercise. Across all groups, most respondents indicated exercising sometimes, while regular exercise was reported by a smaller proportion, again more frequently among senior academics. Early-career academics more often reported limited or no time for exercise.

### Associations between risk and protective factors and mental health outcomes

Table 5 presents the results of multiple regression analyses predicting various indicators of mental health and well-being. Across all outcomes, publication stress, personal mastery, and workplace safety consistently emerged as significant predictors across outcomes, with personal mastery showing the strongest associations. Higher publication stress was associated with poorer mental health (*β* = −.15, *p* < .001), lower work satisfaction (*β* = −.21, *p* < .001), reduced happiness at work (*β* = −.19, *p* < .001), and elevated psychological distress (stress: *β* = .14; anxiety: *β* = .14; depression: *β* = .11; emotional exhaustion: *β* = .20; all *p*s < .001). In contrast, higher personal mastery was associated with better mental health (*β* = .39, *p* < .001), greater work satisfaction (*β* = .33, *p* < .001), higher happiness (*β* = .37, *p* < .001), and lower stress (*β* = −.50, *p* < .001), anxiety (*β* = −.40, *p* < .001), depression (*β* = −.41, *p* < .001), and emotional exhaustion at work (*β* = −.31, *p* < .001). Experiencing less unwanted behavior at the workplace was related to better mental health (*β* = −.12, *p* < .001), higher work satisfaction (*β* = −.11, *p* < .001), greater happiness (*β* = −.16, *p* < .001), as well as lower psychological distress, including reduced stress (*β* = .10, *p* < .001), anxiety (*β* = .15, p < .001), depression (*β* = .13, *p* < .001), and emotional exhaustion (*β* = .12, *p* < .001).

**Table 5:** Summary of multiple regression models for mental health outcomes.

	Well-being	Work Satisfaction	Happiness at Work	Stress	Anxiety	Depression	Emotional Exhaustion at Work
Model Summary	*R²* = .47 adj. *R²* = .46 *F*(18, 1226) = 59.90 *p* < .001	*R²* = .41 adj. *R²* = .40 *F*(18, 1226) = 46.30 *p* < .001	*R²* = .46 adj. *R²* = .46 *F*(18, 1226) = 58.70 *p* < .001	*R²* = .53 adj. *R²* = .53 *F*(18, 1226) = 78.00 *p* < .001	*R²* = .43 adj. *R²* = .42 *F*(18, 1226) = 50.40 *p* < .001	*R²* = .49 adj. *R²* = .48 *F*(18, 1226) = 64.80 *p* < .001	*R²* = .52 adj. *R²* = .51 *F*(18, 1226) = 73.20 *p* < .001
**Predictors**	***β***						
Gender^a^	−.00	.01	.03	.06	.06	.04	.02
Age	.08	.03	.05	**−.15**	−.12	**−.10**	**−.16**
Marital Status^b^	.02	.00	.02	.01	−.01	−.02	−.00
Academic Rank^c^	−.00	**.10**	.04	.06	.05	−.01	.03
Type of University^d^	−.02	−.06	−.01	.05	.05	.05	.04
Geographical Location^e^	.00	−.03	.03	−.01	−.03	.01	**−.07**
Income^f^	.03	.02	.04	−.02	−.03	−.04	.01
Secondary (Non-Academic) Employment^g^	−.01	.03	.01	.01	−.02	−.02	.00
Publication Stress	**−.15**	**−.21**	**−.19**	**.14**	**.14**	**.11**	**.20**
Workload: Weekly work hours^h^	.01	−.01	−.02	.02	.04	.02	**.12**
Workload: Work on weekends and holidays^i^	−.02	−.01	−.01	.00	.02	.08	**.09**
Workload: Work overtime	.00	−.07	−.04	.02	.06	.05	**.12**
Unwanted Behavior at the Workplace^k^	**−.12**	**−.11**	**−.16**	**.10**	**.15**	**.13**	**.12**
Personal Mastery	**.39**	**.33**	**.37**	**−.50**	**−.40**	**−.41**	**−.31**
Relational: Number of significant others^l^	**.08**	.07	.06	−.02	−.01	−.02	−.03
Relational: Quality time with significant others^m^	.06	−.03	.02	−.03	−.01	−.05	.03
Self-care: Time to do hobbies^n^	.07	**.10**	**.10**	−.08	−.07	−.07	**−.11**
Self-care: Time to exercise^o^	**.09**	.03	.00	−.02	−.03	**−.10**	−.02

Significance levels were adjusted using Bonferroni correction (** *p* < .003). Bold values indicate statistically significant results.

^a^Gender = (0) Male, (1) Female.

^b^Marital Status = (0) Not married, (1) Married.

^c^Academic Rank = (1) Teaching staff, (2) Lecturer, (3) Assistant professor, (4) Associate professor, (5) Professor.

^d^Type of University = (0) Public, (2) Private. ^e^Geographical Location = (0) Java island, (1) Other island.

^f^Income = (1) <IDR 3,000,000 [<USD 167], (2) IDR 3,000,000 – IDR 4,999,999 [USD 167–278], (3) IDR 5,000,000 – IDR 9,999,999 [USD 278–556], (4) IDR 10,000,000 – IDR 19,999,999 [USD 556–1,111], (5) IDR 20,000,000 – IDR 49,999,999 [USD 1,111–2,778].

^g^Secondary (Non-Academic) Employment = (0) No, (1) Yes.

^h^Workload: Weekly work hours = (1) 1–8 hours, (2) 9–16 hours, (3) 16–24 hours, (4) 25–32 hours, (5) 33–40 hours, (6) 41–48 hours, (7) 49–56 hours, (8) more than 56 hours.

^i^Workload: Work on weekends and holidays = (1) (Almost) never, (2) 1–2 weekends/holidays every 6 months, (3) 1–2 weekends/holidays every 3 months, (4) 1–2 weekends/holidays every month, (5) Most weekends/holidays.

^j^Workload: Work overtime and at night = (1) (Almost) never, (2) 1–2 afternoons/evenings each month, (3) 1–2 afternoons/evenings each week, (4) 3 or more afternoons/evenings each week, (5) Most afternoons/evenings.

^k^Unwanted Behavior at the workplace = (0) Did not experience unwanted behavior (1) Experienced unwanted behavior.

^l^Relational: Number of significant others = (1) 0–1, (2) 2–5, (3) 6–10, (4) 11–15, (5) 16–20, (6) > 20.

^m^Relational: Quality time with significant others = (1) No, (2) Sometimes, (3) Yes.

^n^Self-care: Time to do hobbies = (1) No, (2) Sometimes, (3) Yes.

^o^Self-care: Time to exercise = (1) No, (2) Sometimes, (3) Yes, regularly.

Several demographic and contextual variables significantly associated with mental health outcomes. Older academics reported lower psychological distress, including reduced stress (*β* = −.15, *p* < .001), anxiety (*β* = −.12, *p* < .001), depression (*β* = −.10, *p* < .001), and emotional exhaustion (*β* = −.16, *p* < .001). Additionally, having sufficient time to engage in hobbies was linked to better well-being, reflected in higher work satisfaction (*β* = .10, *p* < .001) and happiness (*β* = .10, *p* < .001), alongside lower stress (*β* = .08, *p* < .001) and emotional exhaustion (*β* = .11, *p* < .001).

A more detailed examination of emotional exhaustion highlighted the differential roles of protective and risk factors. Lower emotional exhaustion was associated with older age (*β* = −.16, *p* < .001), residence outside Java (*β* = −.07, *p* < .001), higher personal mastery (*β* = −.31, *p* < .001), and having adequate time for hobbies (*β* = −.11, *p* < .001). In contrast, higher emotional exhaustion was associated with greater publication stress (*β* = .20, *p* < .001), more unwanted behavior at workplace (*β* = .12, *p* < .001), and higher workload demands, including longer weekly working hours (*β* = .12, *p* < .001), working during weekends or holidays (*β* = .09, *p* < .001), and particularly night or overtime work (*β* = .12, *p* < .001).

The complete statistical results are provided in S4 Table.

## Discussion

This study aimed to examine the current state of mental health among Indonesian academics, explore differences across demographic and institutional groups, and investigate the association of risk and protective factors with mental-health outcomes. The findings showed that most participants experienced mild to moderate stress and anxiety, while severe psychiatric symptoms were uncommon. Female academics, younger individuals, academics without children, and those in lower academic ranks with lower income appeared to be more vulnerable, as they were associated with poorer mental health outcomes. Across the risk and protective factors examined, publication stress and unwanted behavior at the workplace was consistently associated with lower levels of well-being and higher psychological distress. In contrast, personal mastery was consistently associated with better mental-health indicators, including greater well-being, work satisfaction, and happiness at work, as well as lower stress, anxiety, depression, and emotional exhaustion.

Overall, we found that Indonesian academics reported mild to moderate levels of stress, anxiety, and depression. A similar result was reported in academics in Qatar that shows between one-third and two-third of academics experienced at least a moderate level of stress, anxiety, and depression [71]. It is widely recognized that academics have heavy workloads [5,72,73]. This also reflected in our finding that 40,5% academics work more than 40 hours per week, even in evenings, weekends, and holidays. This exceeded the maximum Indonesian work hours (40 hours a week) according to the Law of the Republic of Indonesia No. 6/2023 (Article 77) [74]. Interestingly, although workload was associated with emotional exhaustion, it was not associated with stress, anxiety, or depression. One possible explanation, which was not examined in the present study, is that academic work may be driven by intrinsic motivation and a strong sense of calling and commitment to service, commonly referred to as *pengabdian* in the Indonesian context, which may be associated with lower levels of psychological distress [75]. Future qualitative research could explore whether these factors shape how academics experience workload and psychological distress.

To highlight the positive aspects of mental health, we descriptively compared our findings with previous studies in Indonesian population using the same instrument. The mean well-being score (MHC-SF) in our sample (M = 62.73) appears slightly lower than that reported among teachers (M = 69.3) [76], yet higher than the average score found in the general Indonesian population aged 18–40 years (M = 49.21) [77]. A similar pattern emerged for life satisfaction. The mean score in our sample (M = 24.47) was higher than that observed in a large Indonesian sample aged 14–50 years, in which men scored an average of M = 15.74 and women M = 16.60 [53]. In terms of happiness, our sample also reported a higher mean score (M = 20.14) compared to Indonesian female university lecturers in a previous study (M = 18.42) [78]. While these comparisons offer valuable descriptive context, they should be interpreted with caution given that the samples were drawn from different populations and contexts, and no direct statistical comparisons were conducted. Even so, the overall descriptive pattern suggests that academics may experience relatively favorable levels of well-being, life satisfaction, and happiness compared to the general population and certain academic subgroups.

Significant differences in mental-health status emerged across demographic groups. Female, younger, and academics without child/children, as well as those in lower academic ranks and lower income groups, reported poorer mental health than their counterparts. These results align with various studies among academics. Female academics tend to report poorer mental health [14]. This pattern mirrors broader epidemiological evidence indicating that women are generally more likely than men to experience common mental health problems, including anxiety and depression [79,80]. Such vulnerabilities are further compounded by the dual demands of professional responsibilities and domestic obligations, which can limit women’s opportunities for career advancement and may be linked to persistent stress and emotional strain [15].

Concerning academic rank, we found that full professors experienced best mental health conditions compared to academics in lower ranks. A study by Saeed et al. [19] involving academics in Pakistan reported consistent findings, showing that individuals in lower academic ranks, such as lecturers and assistant professors, had a higher likelihood of experiencing depression and anxiety than full professors. This pattern has been linked to the heavier workloads carried by junior academics. In our study, however, we found that workloads were relatively similar across academic ranks. The distinguishing factor was that higher-ranking academics reported greater opportunities to pursue hobbies and self-care practices, which may act as protective mechanisms that promote better mental health.

Our study also found that academics without children were more vulnerable to mental health problems, regardless of their marital status. One possible explanation is that this association may partly reflect career stage rather than parenthood status per se. Previous research suggests that many academics postpone parenthood until their careers become more established, indicating that childlessness during this period often reflects career timing rather than a permanent life choice [81]. Consequently, some academics without children may be at earlier career stages, during which structural and career-related pressures, including establishing research productivity, securing stable employment, obtaining permanent positions, and meeting promotion expectations, are particularly pronounced and may adversely affect mental health [82]. Consistent with this interpretation, a recent systematic review identified childlessness as one of the characteristics associated with poorer mental health among university academics, although the underlying mechanisms remain unclear [45]. In the Indonesian context, cultural values surrounding parenthood may also influence how this life stage is experienced, as children are widely perceived as blessings and sources of joy and hope [83], and parenthood is often regarded as an important life transition [84]. However, these cultural mechanisms were not examined in the present study and should be explored in future research.

In examining risk factors, publication pressure emerged as a salient risk factor associated with academic mental health. Our findings indicate that these publication demands significantly associated with symptoms of stress, anxiety, depression, and emotional exhaustion. International research similarly demonstrates that performance-based university rankings and competition for publication output intensify psychological strain [3,7]. The psychological burden is further intensified by how the Indonesian higher-education system structurally ties publication output to career advancement, financial incentives, and institutional performance measures. Promotion to higher academic ranks requires publication in accredited national or reputable international journals, while many universities offer monetary rewards for Scopus-indexed outputs. This creates a high-stakes environment where output quantity becomes synonymous with academic worth, recognition and prestige. Yet the institutional ecosystem does not provide the necessary scaffolding to support these expectations. As reported in Hanami et al. [39], Indonesian academics face limited research funding, heavy teaching loads, inadequate mentoring in research writing, low English academic literacy, and the high cost of open-access publication fees, which are often borne personally due to limited subsidy systems. However, these associations may be bidirectional, as higher emotional exhaustion may also influence how academics perceive publication demands and other workplace experiences.

Another important risk factor associated with poorer mental health was the experience of unwanted behavior at the workplace. Academics who reported experiencing unwanted workplace behaviors showed lower levels of well-being, work satisfaction, and happiness at work, together with higher levels of stress, anxiety, depression, and emotional exhaustion. Previous studies have demonstrated that workplace bullying is associated with poorer psychological well-being and increased psychological distress among employees [85]. Workplace bullying has also been linked to adverse emotional health outcomes and reduced employee well-being through its effects on organizational climate and workplace experiences [86]. Furthermore, exposure to workplace bullying has been associated with diminished self-worth and poorer psychological functioning [87]. Recent multinational evidence further confirmed that workplace offensive behaviors are consistently associated with adverse mental health outcomes across occupational settings [88]. Similarly, both experiencing and witnessing workplace bullying have been linked to psychological distress, sickness absence, and unfavorable organizational outcomes [89].

In our study, about 12% of academics reported experiencing such incidents, most often involving supervisors or colleagues. Most respondents identified themselves as victims, while only a few reported perpetration or witnessing incidents. Participants reported a broad spectrum of unwanted behaviors and workplace violence within academic settings. Verbal violence was the most frequently mentioned, ranging from harsh criticism and public humiliation to negative labeling by colleagues or superiors. This finding shows that incivility and verbal aggression are among the most normalized yet damaging forms of violence in higher education settings, primarily because they are often discursively framed as feedback, discipline, or professional standards [90,91]. Furthermore, the prevalence of threats and intimidation also confirms that fear is often used as an organizing force in many of our participants’ professional lives. The acts, which range from power abuse by superiors to systematic exclusion and obstruction of work–are aligned with the evidence in many other countries highlighting academic bullying being structurally embedded in competitive, hierarchical, and precarious work environments [92,93]. One possible interpretation is that bullying may also function as a means of maintaining authority and discouraging dissent, although this was not directly examined in the present study.

The additional categories deepen the structural nature of this violence. Unfair evaluations and career obstruction which constitute professional injustice demonstrate how harm can be delivered through formal bureaucratic mechanisms, rather than overt aggression. This data reflects the recent critiques on managerial university, in which opaque evaluation systems can lead to administrative violence [94]. In addition, character defamation and slander suggests the presence of reputational violence, which is potentially linked to career survival [93]In addition to these risk factors, our findings also highlighted potential protective factors.. In our findings, perceived personal mastery was consistently associated with better mental-health outcomes. This result echoes previous studies showing that a higher sense of mastery is linked to lower depressive symptoms and anxiety [95,96]. In occupational settings, perceived control, which is closely related to mastery, has been shown to reduce work-related stress and depression while increasing work engagement and satisfaction [97]. Evidence from longitudinal research also supports these associations, indicating that mastery predicts lower psychological distress, reduced depression risk, and greater resilience over time across different populations, including mid-aged adults [98], patients with chronic illness [99,100], and healthy adults [101].

These empirical patterns align with Pearlin and Schooler’s stress and coping framework, which conceptualizes mastery as a sense of personal control over life circumstances [102]. Individuals with higher mastery believe they can influence outcomes rather than feeling powerless in the face of external pressures [103]. Within this framework, mastery functions as a core psychological resource related to stress appraisal and coping strategies, as well as to better regulation of emotional responses and greater psychological resilience. Conversely, low mastery is associated with passive coping, helplessness, and higher vulnerability to stress-related symptoms [102,103]. Given the structural pressures in academia, such as publication demands, competition, and workload, personal mastery may be associated with a stronger sense of agency, better wellbeing, and lower level of psychological strain related to academic work.

This study offers several important strengths. It is the first national study to examine the mental health of academics in Indonesia, providing foundational empirical evidence in a previously understudied context. The large sample size, drawn from multiple provinces, institutional types (public and private), and academic ranks, allows for a broad overview of mental-health patterns within the higher-education sector. In addition, the use of a questionnaire that is comparable to instruments applied in studies from the Netherlands [104] enables cross-country comparison and the potential for meaningful international benchmarking.

Nonetheless, several limitations should be acknowledged. First, the cross-sectional design restricts interpretation to non-causal associations. Second, the exclusive reliance on self-report instruments may introduce social desirability and underreporting biases, despite the use of validated measures. Third, although the sample is large, it is not fully representative of Indonesia’s academic workforce. Paid social media promotion helped achieve broad national coverage, however, recruitment relied on voluntary participation and social media exposure. As a result, the sample may not be fully representative of all Indonesian academics, as early-career academics, who are generally more active on social media, may have been more likely to participate. Furthermore, the number of participants recruited from each institution may have varied, and institutional clustering was not accounted for in the analyses. As academics within the same institution may share similar organizational contexts, policies, and working environments, potential non-independence of observations cannot be ruled out and may have affected the precision of the estimated associations.

Fourth, potential selection or response bias cannot be ruled out, as academics with stronger views or specific experiences may have been more motivated to participate. The completion rate was 51.6% of those who accessed the survey link (59.8% of consenting participants). Attrition analyses indicated that completers and non-completers were largely comparable across most demographic characteristics. Yet differences by institution type and geographic location suggest that some degree of selection bias cannot be ruled out. In addition, only a minority of the broader eligible academic community participated in the survey, which may limit the representativeness of the sample. Similar challenges with participation rates have also been reported in large-scale surveys of academic populations [105]. Finally, the use of the Bonferroni correction to account for multiple comparisons. Although this approach reduces the risk of Type I error, it is conservative and may increase the likelihood of Type II error. Consequently, non-significant results may still be true effects and should be taken into account in future research endeavours.

In conclusion, this national survey suggests that the academics who participated reported mild to moderate levels of stress and anxiety, while severe psychiatric symptoms were relatively uncommon. Within this sample, women, younger academics, unmarried academics, and those in lower academic ranks and income groups appeared to report greater psychological vulnerability. Among the risk factors assessed, publication related stress and unwanted behavior in the workplace emerged as a consistent correlate of reduced well-being and greater psychological distress. In contrast, personal mastery functioned as an important protective correlate, showing consistent associations with higher levels of well-being, work satisfaction, and happiness at work, as well as lower levels of stress, anxiety, depression, and emotional exhaustion. The relatively strong association observed for mastery (*β* = .31 −.50) further suggest that it may represent a relevant focus for future research and intervention development. These findings point to several practical priorities for supporting mental health in academia. At the individual level, initiatives that strengthen personal mastery and coping resources may be beneficial. At the institutional level, universities should consider strategies to reduce excessive publication-related pressure, prevent and respond appropriately to unwanted behavior in the workplace, and provide additional support for groups identified as more vulnerable. Reviewing workload expectations, performance evaluation systems, career pathways, and access to mental health support may also contribute to healthier academic working environments. Future research should develop and test such approaches using longitudinal, intervention, and mixed-method designs to better understand their effectiveness and the direction of the observed associations. At a broader level, universities and national stakeholders should work toward organizational cultures and policies that support open discussion of mental health and promote sustainable and supportive working conditions for academics.

## Supporting information

S1 Table Summary of the study measures. (DOCX)

S2 Table Comparison of completers and non-completers on demographic characteristics. (DOCX)

S3 Table Summary of independent t-test and one-way ANOVA results. (DOCX)

S4 Table Results of multiple regression analyses predicting mental health. (DOCX)
