# Supplementary material for: Mental health in academia: A national survey on risk and protective factors in the Indonesian context
Source: PLoS One. 2026 Sep 21;21(9):e0358652. doi: 10.1371/journal.pone.0358652 (PMC13599600; doi:10.1371/journal.pone.0358652)
Supplement: Supplementary file 1 [file pone.0358652.s007.pdf]

Secon revision of the paper titled Mental health in academia: A national survey on risk and protective factors in the Indonesian context

Dear Authors

I carefully read the revised paper and your detailed point by point replies to my comments. All 13 points raised by my review have been addressed and the quality of paper has substantially improved. Almost all points do not need further refinement, but some of them (n. 2 and 12) need, in my view, minor revisions

Specifically,

Point 1 "*The introduction could be improved by better highlighting the reason because mental health of academics needs attention and what would be the key-points for this, even by stressing the link between academic performance and academics' mental health. It would be useful also to have a more in-depth description of the variables of the previous studies on the same topic cited (i.e. gender, age, status as possible risk and/or risk factors"* has been fully addressed both in text and in the response letter

Point 2 concerning the part related to "*It is expected to have the number and ratio of participants who accepted the invitation to collaborate in the survey already here and a mention on missing data treatment. It is not clear how many participants accepted and how many were invited"* has been well addressed by adding essential information on participants flow. Nevertheless, from your accurate description it appears that only a minority of the whole eligible group of participants accepted to take part in the survey. This needs to be fully discussed in the conclusion and limitation section, because is part of the ecological validity of your study. I understand that the issue of completers vs non completers and the attrition analysis has been fully addressed, but it needs to be clear to the reader that only a part of the academic faculty community accepted to participate, possibly with a mention of similar studies that reported this data.

Point 3 *Description of measures* has been successfully improved.

Point 4 The inclusion of a summary table for instruments and their description would be a helpful contribution of the clarity of your paper

Point 5 The inclusion of a further CFA analysis, as suggested, fully clarified and completed the psychometric properties of all adapted or modified measures used in your study

Point 6 Missing data and attrition rate analysis for comparing completers and non completers added to your paper, fully contributed to reliability of your data

Point 7 The completion of information on table 2 results fully addressed my point

Point 8 The completion of this information by adding the model building strategy for regression analyses contributed in clarifying this aspect

Point 9 The specification of the coding of the categorical predictors in the regression analyses are now clear

Point 10 The accepted suggestion to assess multicollinearity contributed in full reporting regression analyses results

Point 11 table 5 is now reporting in a correct and complete way and allows the reader to properly catch the information on mental health levels and its correspondence with the severity categories reported for each measure in the Measures section

Point 12 page 40-41 on discussion, lines 748-771 needs, in my view, to be reworded in a more nuanced and cautious way, given that the sample inquired on all variables is only a small part of the whole eligible academic faculty population. This point is a strength and, at the same time, a limitation of your study. You reached a large sample which is, nevertheless, only a part of the whole population.

Point 13 the reduction of causal language improve the reliability of your conclusion.

To sum up my final comments, I found the paper significantly improved, with main points raised in the revision process accurately addressed by the Authors. Only 2 points need a further refinement as minor revisions.

Lavinia Barone

University of Pavia, IT
