# Supplementary material for: Mental health in academia: A national survey on risk and protective factors in the Indonesian context
Source: PLoS One. 2026 Sep 21;21(9):e0358652. doi: 10.1371/journal.pone.0358652 (PMC13599600; doi:10.1371/journal.pone.0358652)
Supplement: Supplementary file 2 [file pone.0358652.s001.docx]

**S1 Table. Summary of the Study Measures.**

| **Construct** | **Instrument** | **Items** | **Response Scale** | **Score Range** | **Higher Scores Indicate** | **Cronbach's α (Present Study)** | **Validation/Adaptation** |
| --- | --- | --- | --- | --- | --- | --- | --- |
| Well-being | Mental Health Continuum-Short Form (MHC-SF) [49] | 14 | 0 (Never) – 5 (Every day) | 0 – 70 | Better well-being | .93 | Indonesian validated version [50] |
| Work satisfaction | Satisfaction With Work Scale (SWWS) [51], adapted to work context | 5 | 1 (Strongly disagree) – 7 (Strongly agree) | 5 – 35 | Greater work satisfaction | .90 | Forward–backward translated into Indonesian |
| Happiness at work | Subjective Happiness Scale (SHS) [55], adapted to work context | 4 | 1 (Strongly disagree) – 7 (Strongly agree) | 4 – 28 | Greater happiness at work | .83 | Forward–backward translated; adapted to work context |
| Stress | Perceived Stress Scale (PSS-10) [56] | 10 | 0 (Never) – 4 (Very often)  Items 4, 5, 7, and 8 reverse-coded | 0 – 40 | Greater perceived stress | .82 | Indonesian validated version [57=58] |
| Anxiety | Generalized Anxiety Disorder Scale (GAD-7) [63] | 7 | 0 (Not at all) – 3 (Nearly every day) | 0 – 21 | Greater anxiety | .91 | Indonesian validated version [64] |
| Depression | Quick Inventory of Depressive Symptomatology-Self Report (QIDS-SR) [65] | 16 | 0 – 3 | 0 – 27 | Greater depressive symptoms | .80 | Indonesian adaptation from IDS-SR [66] |
| Emotional exhaustion | Maslach Burnout Inventory-Emotional Exhaustion subscale (MBI) [68] | 7 | 0 (Never) – 6 (Every day) | 0 – 42 | Greater emotional exhaustion | .92 | Forward–backward translated into Indonesian |
| Publication stress | Publication Pressure Questionnaire–Revised (PPQ-r) [69] | 6 | 1 (Totally disagree) – 5 (Totally agree) | 6 – 30 | Greater publication stress | .84 | Forward–backward translated into Indonesian |
| Workload | Three self-developed questions | 3 | Categorical | — | Greater workload | — |  |
| Experiences of unwanted behavior at the workplace | Self-developed questions | Multiple | Dichotomous & categorical follow-up | — | Exposure to unwanted workplace behavior | — |  |
| Personal mastery | Pearlin Mastery Scale [70] | 7 | 1 (Strongly disagree) – 5 (Strongly agree)  items 1, 2, 3, 5, and 7; reverse-coded | 7 – 35 | Greater perceived mastery | .78 | Forward–backward translated into Indonesian |
| Relational coping | Two self-developed questions | 2 | Categorical | — | Greater interpersonal support | — |  |
| Self-care coping | Two self-developed questions | 2 | Categorical | — | Greater engagement in self-care | — |  |
