# Supplementary material for: Mental health in academia: A national survey on risk and protective factors in the Indonesian context
Source: PLoS One. 2026 Sep 21;21(9):e0358652. doi: 10.1371/journal.pone.0358652 (PMC13599600; doi:10.1371/journal.pone.0358652)
Supplement: Supplementary file 3 [file pone.0358652.s002.docx]

**S2 Table. Comparison of Completers and Non-Completers on Demographic Characteristics.**

| **Characteristic** | **Completers** | | **Non-completers** | | **χ^2^(df)** | **p** |
| --- | --- | --- | --- | --- | --- | --- |
|  | **n** | **%** | **n** | **%** |  |  |
| **Gender** |  |  |  |  | .021 | .886 |
| Male | 512 | 37.18 | 312 | 36.88 |  |  |
| Female | 865 | 62.82 | 534 | 63.12 |  |  |
| **Age Group** |  |  |  |  | 6.80 | .339 |
| <26 | 9 | 0.66 | 6 | 0.72 |  |  |
| 26-30 | 192 | 13.97 | 134 | 16.07 |  |  |
| 31-40 | 642 | 46.72 | 381 | 45.68 |  |  |
| 41-50 | 312 | 22.71 | 186 | 22.30 |  |  |
| 51-60 | 171 | 12.45 | 86 | 10.31 |  |  |
| 61-70 | 46 | 3.35 | 38 | 4.56 |  |  |
| >71 | 2 | 0.15 | 3 | 0.36 |  |  |
| **Marital and Parental Status** |  |  |  |  | 4.52 | .211 |
| Not married without children | 253 | 18.35 | 164 | 19.39 |  |  |
| Not married with children | 56 | 4.06 | 22 | 2.60 |  |  |
| Married without children | 146 | 10.59 | 79 | 9.34 |  |  |
| Married with children | 924 | 67.01 | 581 | 68.68 |  |  |
| **Academic Rank** |  |  |  |  | .98 | .91 |
| Teaching staff | 215 | 15.60 | 122 | 16.64 |  |  |
| Lecturer | 428 | 31.06 | 219 | 29.88 |  |  |
| Assistant professor | 555 | 40.28 | 302 | 41.20 |  |  |
| Associate professor | 145 | 10.52 | 74 | 10.10 |  |  |
| Professor | 35 | 2.54 | 16 | 2.18 |  |  |
| **Type of University** |  |  |  |  | 9.04 | .029 |
| Public - fully autonomous | 157 | 11.45 | 102 | 13.92 |  |  |
| Public - semi-autonomous | 164 | 11.96 | 105 | 14.32 |  |  |
| Public - non-autonomous | 61 | 4.45 | 43 | 5.87 |  |  |
| Private | 989 | 72.14 | 483 | 65.89 |  |  |
| **Geographical Location** |  |  |  |  | 5.06 | .025 |
| Java island | 861 | 63.68 | 412 | 58.61 |  |  |
| Other islands | 491 | 36.32 | 291 | 41.39 |  |  |
| **Income** |  |  |  |  | 10.95 | .052 |
| < IDR 3,000,000 | 234 | 16.97 | 124 | 16.53 |  |  |
| IDR 3,000,000 – IDR 4,999,999 | 418 | 30.31 | 235 | 31.33 |  |  |
| IDR 5,000,000 – IDR 9,999,999 | 458 | 33.21 | 272 | 36.27 |  |  |
| IDR 10,000,000 – IDR 19,999,999 | 211 | 15.30 | 104 | 13.87 |  |  |
| IDR 20,000,000 – IDR 49,999,999 | 58 | 4.21 | 15 | 2.00 |  |  |
| **Secondary (Non-Academic) Employment** |  |  |  |  | .88 | .350 |
| No | 673 | 49.49 | 382 | 51.62 |  |  |
| Yes | 687 | 50.51 | 358 | 48.38 |  |  |
