# Supplementary material for: Mental health in academia: A national survey on risk and protective factors in the Indonesian context
Source: PLoS One. 2026 Sep 21;21(9):e0358652. doi: 10.1371/journal.pone.0358652 (PMC13599600; doi:10.1371/journal.pone.0358652)
Supplement: Supplementary file 4 [file pone.0358652.s003.docx]

**S3 Table. Summary of Independent t-test and One-Way ANOVA Results.**

| **Variable** | **Well-being** | **Work Satisfaction** | **Happiness at Work** | **Stress** | **Anxiety** | **Depression** | **Emotional Exhaustion at Work** |
| --- | --- | --- | --- | --- | --- | --- | --- |
| Gender | t(1375) = 2.87, p = .004, p_adj = .029, Cohen’s d = 0.16 | t(1375) = 1.98, p = .048, p_adj = .338, Cohen’s d = 0.11 | t(1375) = 1.33, p = .185, p_adj = 1.000, Cohen’s d = 0.07 | t(1375) = -4.31, p < .001, p_adj < .001, Cohen’s d = -0.24 | t(1375) = -4.17, p < .001, p_adj < .001, Cohen’s d = -0.23 | t(1375) = -4.19, p < .001, p_adj < .001, Cohen’s d = -0.23 | t(1375) = -3.43, p = .001, p_adj = .004, Cohen’s d = -0.19 |
| Age Group | F(6, 1367) = 14.36, p < .001, p_adj < .001, η²ₚ = 0.06 | F(6, 1367) = 10.89, p < .001, p_adj < .001, η²ₚ = 0.05 | F(6, 1367) = 12.12, p < .001, p_adj < .001, η²ₚ = 0.05 | F(6, 1367) = 18.83, p < .001, p_adj < .001, η²ₚ = 0.08 | F(6, 1367) = 13.47, p < .001, p_adj < .001, η²ₚ = 0.06 | F(6, 1367) = 18.45, p < .001, p_adj < .001, η²ₚ = 0.07 | F(6, 1367) = 17.38, p < .001, p_adj < .001, η²ₚ = 0.07 |
| Marital Status | t(1378) = -3.96, p < .001, p_adj = .001, Cohen’s d = -0.26 | t(1378) = -3.37, p = .001, p_adj = .005, Cohen’s d = -0.22 | t(1378) = -3.86, p < .001, p_adj = 0.001, Cohen’s d = -0.25 | t(1378) = 3.02, p = .003, p_adj = .018, Cohen’s d = 0.19 | t(1378) = 2.85, p = .004, p_adj = .031, Cohen’s d = 0.18 | t(1378) = 4.13, p < .001, p_adj < .001, Cohen’s d = 0.27 | t(1378) = 2.91, p = .004, p_adj = .026, Cohen’s d = 0.19 |
| Marital Status | F(3, 1376) = 13.01, p < .001, p_adj < .001, η²ₚ = 0.03 | F(3, 1376) = 11.72, p < .001, p_adj < .001, η²ₚ = 0.02 | F(3, 1376) = 13.87, p < .001, p_adj < .001, η²ₚ = 0.03 | F(3, 1376) = 10.12, p < .001, p_adj < .001, η²ₚ = 0.02 | F(3, 1376) = 11.40, p < .001, p_adj < .001, η²ₚ = 0.02 | F(3, 1376) = 14.42, p < .001, p_adj < .001, η²ₚ = 0.03 | F(3, 1376) = 14.63, p < .001, p_adj < .001, η²ₚ = 0.03 |
| Academic Rank | F(4, 1373) = 13.98, p < .001, p_adj < .001, η²ₚ = 0.04 | F(4, 1373) = 19.30, p < .001, p_adj < .001, η²ₚ = 0.05 | F(4, 1373) = 12.12, p < .001, p_adj < .001, η²ₚ = 0.04 | F(4, 1373) = 11.68, p < .001, p_adj < .001, η²ₚ = 0.03 | F(4, 1373) = 7.22, p < .001, p_adj < .001, η²ₚ = 0.02 | F(4, 1373) = 13.04, p < .001, p_adj < .001, η²ₚ = 0.04 | F(4, 1373) = 9.40, p < .001, p_adj < .001, η²ₚ = 0.03 |
| Type of University | F(3, 1367) = 1.50, p = .213, p_adj = 1.000, η²ₚ = 0.00 | F(3, 1367) = 0.80, p = .498, p_adj = 1.000, η²ₚ = 0.00 | F(3, 1367) = 2.49, p = .058, p_adj = .409, η²ₚ = 0.01 | F(3, 1367) = 0.60, p = .616, p_adj = 1.000, η²ₚ = 0.00 | F(3, 1367) = 0.71, p = .545, p_adj = 1.000, η²ₚ = 0.00 | F(3, 1367) = 0.35, p = .788, p_adj = 1.000, η²ₚ = 0.00 | F(3, 1367) = 2.55, p = .054, p_adj = .381, η²ₚ = 0.01 |
| Geographical Location | t(1350) = -.14, p = .890, p_adj = 1.000, Cohen’s d = -0.01 | t(1350) = 0.56, p = .576, p_adj = 1.000, Cohen’s d = 0.03 | t(1350) = -1.21, p = .227, p_adj = 1.000, Cohen’s d = -0.07 | t(1350) = 0.93, p = .353, p_adj = 1.000, Cohen’s d = 0.05 | t(1350) = 1.32, p = .187, p_adj = 1.000, Cohen’s d = 0.07 | t(1350) = -0.52, p = .601, p_adj = 1.000, Cohen’s d = -0.03 | t(1350) = 2.95, p = .003, p_adj = .022, Cohen’s d = 0.17 |
| Income | F(4, 1374) = 9.32, p < .001, p_adj < .001, η²ₚ = 0.03 | F(4, 1374) = 11.90, p < .001, p_adj < .001, η²ₚ = 0.03 | F(4, 1374) = 10.64, p < .001, p_adj < .001, η²ₚ = 0.03 | F(4, 1374) = 14.34, p < .001, p_adj < .001, η²ₚ = 0.04 | F(4, 1374) = 4.96, p = .001, p_adj = .004, η²ₚ = 0.01 | F(4, 1374) = 12.71, p < .001, p_adj < .001, η²ₚ = 0.04 | F(4, 1374) = 5.67, p < .001, p_adj = .001, η²ₚ = 0.02 |
| Having a Side Job | t(1358) = -5.01, p < .001, p_adj < .001, Cohen’s d = -0.27 | t(1358) = -2.99, p = .003, p_adj = .020, Cohen’s d = -0.16 | t(1358) = -4.26, p < .001, p_adj < .001, Cohen’s d = -0.23 | t(1358) = 4.11, p < .001, p_adj < .001, Cohen’s d = 0.22 | t(1358) = 3.12, p = .002, p_adj = .013, Cohen’s d = 0.17 | t(1358) = 4.12, p < .001, p_adj < .001, Cohen’s d = 0.22 | t(1358) = 3.15, p = .002, p_adj = .012, Cohen’s d = 0.17 |

**Post Hoc Comparison for Significant Independent Variables**

| **Dependent Variable** | **Independent Variable** | **Comparison** | **MD** | **SE** | **p** | **95% CI** |
| --- | --- | --- | --- | --- | --- | --- |
| Mental Health | Age Group | <26 years - 26-30 years | 3.43 | 4.59 | .990 | -10.13 - 16.98 |
|  |  | <26 years - 31-40 years | 0.07 | 4.52 | 1.000 | -13.27 - 13.41 |
|  |  | <26 years - 41-50 years | -3.21 | 4.55 | .992 | -16.64 - 10.23 |
|  |  | <26 years - 51-60 years | -6.61 | 4.60 | .782 | -20.2 - 6.98 |
|  |  | <26 years - 61-70 years | -10.54 | 4.91 | .325 | -25.02 - 3.95 |
|  |  | <26 years - >71 years | -11.67 | 10.52 | .926 | -42.73 - 19.4 |
|  |  | 26-30 years - <26 years | -3.43 | 4.59 | .990 | -16.98 - 10.13 |
|  |  | 26-30 years - 31-40 years | -3.36^*^ | 1.11 | .039 | -6.62 - -0.09 |
|  |  | 26-30 years - 41-50 years | -6.63^*^ | 1.23 | .000 | -10.27 - -2.99 |
|  |  | 26-30 years - 51-60 years | -10.04^*^ | 1.41 | .000 | -14.21 - -5.86 |
|  |  | 26-30 years - 61-70 years | -13.96^*^ | 2.21 | .000 | -20.48 - -7.44 |
|  |  | 26-30 years - >71 years | -15.09 | 9.57 | .697 | -43.34 - 13.15 |
|  |  | 31-40 years - <26 years | -0.07 | 4.52 | 1.000 | -13.41 - 13.27 |
|  |  | 31-40 years - 26-30 years | 3.36^*^ | 1.11 | .039 | 0.09 - 6.62 |
|  |  | 31-40 years - 41-50 years | -3.28^*^ | .93 | .008 | -6.02 - -0.53 |
|  |  | 31-40 years - 51-60 years | -6.68^*^ | 1.16 | .000 | -10.1 - -3.26 |
|  |  | 31-40 years - 61-70 years | -10.61^*^ | 2.05 | .000 | -16.67 - -4.54 |
|  |  | 31-40 years - >71 years | -11.74 | 9.53 | .882 | -39.88 - 16.41 |
|  |  | 41-50 years - <26 years | 3.21 | 4.55 | .992 | -10.23 - 16.64 |
|  |  | 41-50 years - 26-30 years | 6.63^*^ | 1.23 | .000 | 2.99 - 10.27 |
|  |  | 41-50 years - 31-40 years | 3.28^*^ | 0.93 | .008 | 0.53 - 6.02 |
|  |  | 41-50 years - 51-60 years | -3.40 | 1.28 | .110 | -7.18 - 0.38 |
|  |  | 41-50 years - 61-70 years | -7.33^*^ | 2.13 | .010 | -13.61 - -1.05 |
|  |  | 41-50 years - >71 years | -8.46 | 9.55 | .975 | -36.65 - 19.73 |
|  |  | 51-60 years - <26 years | 6.61 | 4.60 | .782 | -6.98 - 20.20 |
|  |  | 51-60 years - 26-30 years | 10.04^*^ | 1.41 | .000 | 5.86 - 14.21 |
|  |  | 51-60 years - 31-40 years | 6.68^*^ | 1.16 | .000 | 3.26 - 10.10 |
|  |  | 51-60 years - 41-50 years | 3.40 | 1.28 | .110 | -0.38 - 7.18 |
|  |  | 51-60 years - 61-70 years | -3.93 | 2.24 | .577 | -10.53 - 2.67 |
|  |  | 51-60 years - >71 years | -5.06 | 9.57 | .998 | -33.32 - 23.21 |
|  |  | 61-70 years - <26 years | 10.54 | 4.91 | .325 | -3.95 - 25.02 |
|  |  | 61-70 years - 26-30 years | 13.96^*^ | 2.21 | .000 | 7.44 - 20.48 |
|  |  | 61-70 years - 31-40 years | 10.61^*^ | 2.05 | .000 | 4.54 - 16.67 |
|  |  | 61-70 years - 41-50 years | 7.33^*^ | 2.13 | .010 | 1.05 - 13.61 |
|  |  | 61-70 years - 51-60 years | 3.93 | 2.24 | .577 | -2.67 - 10.53 |
|  |  | 61-70 years - >71 years | -1.13 | 9.72 | 1.000 | -29.84 - 27.57 |
|  |  | >7 years - <26 years | 11.67 | 10.52 | .926 | -19.4 - 42.73 |
|  |  | >7 years - 26-30 years | 15.09 | 9.57 | .697 | -13.15 - 43.34 |
|  |  | >7 years - 31-40 years | 11.74 | 9.53 | .882 | -16.41 - 39.88 |
|  |  | >7 years - 41-50 years | 8.46 | 9.55 | .975 | -19.73 - 36.65 |
|  |  | >7 years - 51-60 years | 5.06 | 9.57 | .998 | -23.21 - 33.32 |
|  |  | >7 years - 61-70 years | 1.13 | 9.72 | 1.000 | -27.57 - 29.84 |
|  | Marital Status | Not married without children - Not married with children | -6.245^*^ | 2.02 | .010 | -11.44 - -1.05 |
|  |  | Not married without children - Married without children | -0.81 | 1.42 | .940 | -4.45 - 2.84 |
|  |  | Not married without children - Married with children | -5.273^*^ | 0.97 | .000 | -7.77 - -2.78 |
|  |  | Not married with children - Not married without children | 6.245^*^ | 2.02 | .010 | 1.05 - 11.44 |
|  |  | Not married with children - Married without children | 5.44 | 2.15 | .060 | -0.08 - 10.97 |
|  |  | Not married with children - Married with children | 0.97 | 1.88 | .960 | -3.87 - 5.81 |
|  |  | Married without children - Not married without children | 0.81 | 1.42 | .940 | -2.84 - 4.45 |
|  |  | Married without children - Not married with children | -5.44 | 2.15 | .060 | -10.97 - 0.08 |
|  |  | Married without children - Married with children | -4.468^*^ | 1.21 | .000 | -7.59 - -1.34 |
|  |  | Married with children - Not married without children | 5.273^*^ | 0.97 | .000 | 2.78 - 7.77 |
|  |  | Married with children - Not married with children | -0.97 | 1.88 | .960 | -5.81 - 3.87 |
|  |  | Married with children - Married without children | 4.468^*^ | 1.21 | .000 | 1.34 - 7.59 |
|  | Academic Rank | Teaching staff - Lecturer | 0.45 | 1.14 | .995 | -2.66 - 3.55 |
|  |  | Teaching staff - Assistant professor | -2.13 | 1.09 | .292 | -5.12 - 0.86 |
|  |  | Teaching staff - Associate professor | -6.37^*^ | 1.46 | .000 | -10.37 - -2.38 |
|  |  | Teaching staff - Professor | -13.03^*^ | 2.48 | .000 | -19.8 - -6.25 |
|  |  | Lecturer - Teaching staff | -0.45 | 1.14 | .995 | -3.55 - 2.66 |
|  |  | Lecturer - Assistant professor | -2.58^*^ | 0.88 | .027 | -4.97 - -0.19 |
|  |  | Lecturer - Associate professor | -6.82^*^ | 1.31 | .000 | -10.39 - -3.25 |
|  |  | Lecturer - Professor | -13.48^*^ | 2.39 | .000 | -20.01 - -6.94 |
|  |  | Assistant professor - Teaching staff | 2.13 | 1.09 | .292 | -0.86 - 5.12 |
|  |  | Assistant professor - Lecturer | 2.58^*^ | 0.88 | .027 | 0.19 - 4.97 |
|  |  | Assistant professor - Associate professor | -4.24^*^ | 1.27 | .008 | -7.71 - -0.78 |
|  |  | Assistant professor - Professor | -10.90^*^ | 2.37 | .000 | -17.38 - -4.42 |
|  |  | Associate professor - Teaching staff | 6.37^*^ | 1.46 | .000 | 2.38 - 10.37 |
|  |  | Associate professor - Lecturer | 6.82^*^ | 1.31 | .000 | 3.25 - 10.39 |
|  |  | Associate professor - Assistant professor | 4.24^*^ | 1.27 | .008 | 0.78 - 7.71 |
|  |  | Associate professor - Professor | -6.66 | 2.56 | .071 | -13.66 - 0.34 |
|  |  | Professor - Teaching staff | 13.03^*^ | 2.48 | .000 | 6.25 - 19.8 |
|  |  | Professor - Lecturer | 13.48^*^ | 2.39 | .000 | 6.94 - 20.01 |
|  |  | Professor - Assistant professor | 10.90^*^ | 2.37 | .000 | 4.42 - 17.38 |
|  |  | Professor - Associate professor | 6.66 | 2.56 | .071 | -0.34 - 13.66 |
|  | Income | < Rp 3,000,000 - Rp 3,000,000 - Rp 4,999,999 | 0.18 | 1.12 | 1.000 | -2.88 - 3.23 |
|  |  | < Rp 3,000,000 - Rp 5,000,000 - Rp 9,999,999 | -1.12 | 1.10 | .847 | -4.13 - 1.89 |
|  |  | < Rp 3,000,000 - Rp 10,000,000 - Rp 19,999,999 | -4.85^*^ | 1.30 | .002 | -8.4 - -1.3 |
|  |  | < Rp 3,000,000 - Rp 20,000,000 - Rp 49,999,999 | -8.52^*^ | 2.01 | .000 | -14.01 - -3.04 |
|  |  | Rp 3,000,000 - Rp 4,999,999 - < Rp 3,000,000 | -0.18 | 1.12 | 1.000 | -3.23 - 2.88 |
|  |  | Rp 3,000,000 - Rp 4,999,999 - Rp 5,000,000 - Rp 9,999,999 | -1.30 | 0.93 | .628 | -3.83 - 1.23 |
|  |  | Rp 3,000,000 - Rp 4,999,999 - Rp 10,000,000 - Rp 19,999,999 | -5.03^*^ | 1.16 | .000 | -8.19 - -1.87 |
|  |  | Rp 3,000,000 - Rp 4,999,999 - Rp 20,000,000 - Rp 49,999,999 | -8.70^*^ | 1.92 | .000 | -13.94 - -3.46 |
|  |  | Rp 5,000,000 - Rp 9,999,999 - < Rp 3,000,000 | 1.12 | 1.10 | .847 | -1.89 - 4.13 |
|  |  | Rp 5,000,000 - Rp 9,999,999 - Rp 3,000,000 - Rp 4,999,999 | 1.30 | 0.93 | .628 | -1.23 - 3.83 |
|  |  | Rp 5,000,000 - Rp 9,999,999 - Rp 10,000,000 - Rp 19,999,999 | -3.73^*^ | 1.14 | .009 | -6.84 - -0.62 |
|  |  | Rp 5,000,000 - Rp 9,999,999 - Rp 20,000,000 - Rp 49,999,999 | -7.40^*^ | 1.91 | .001 | -12.62 - -2.19 |
|  |  | Rp 10,000,000 - Rp 19,999,999 - < Rp 3,000,000 | 4.85^*^ | 1.30 | .002 | 1.3 - 8.4 |
|  |  | Rp 10,000,000 - Rp 19,999,999 - Rp 3,000,000 - Rp 4,999,999 | 5.03^*^ | 1.16 | .000 | 1.87 - 8.19 |
|  |  | Rp 10,000,000 - Rp 19,999,999 - Rp 5,000,000 - Rp 9,999,999 | 3.73^*^ | 1.14 | .009 | 0.62 - 6.84 |
|  |  | Rp 10,000,000 - Rp 19,999,999 - Rp 20,000,000 - Rp 49,999,999 | -3.67 | 2.03 | .369 | -9.22 - 1.87 |
|  |  | Rp 20,000,000 - Rp 49,999,999 - < Rp 3,000,000 | 8.52^*^ | 2.01 | .000 | 3.04 - 14.01 |
|  |  | Rp 20,000,000 - Rp 49,999,999 - Rp 3,000,000 - Rp 4,999,999 | 8.70^*^ | 1.92 | .000 | 3.46 - 13.94 |
|  |  | Rp 20,000,000 - Rp 49,999,999 - Rp 5,000,000 - Rp 9,999,999 | 7.40^*^ | 1.91 | .001 | 2.19 - 12.62 |
|  |  | Rp 20,000,000 - Rp 49,999,999 - Rp 10,000,000 - Rp 19,999,999 | 3.67 | 2.03 | .369 | -1.87 - 9.22 |
| Work Satisfaction | Age Group | <26 years - 26-30 years | 0.10 | 2.23 | 1.000 | -6.49 - 6.68 |
|  |  | <26 years - 31-40 years | -0.95 | 2.19 | .999 | -7.43 - 5.53 |
|  |  | <26 years - 41-50 years | -1.42 | 2.21 | .995 | -7.95 - 5.11 |
|  |  | <26 years - 51-60 years | -4.08 | 2.24 | .531 | -10.68 - 2.52 |
|  |  | <26 years - 61-70 years | -5.98 | 2.38 | .157 | -13.02 - 1.06 |
|  |  | <26 years - >71 years | -5.00 | 5.11 | .959 | -20.09 - 10.09 |
|  |  | 26-30 years - <26 years | -0.10 | 2.23 | 1.000 | -6.68 - 6.49 |
|  |  | 26-30 years - 31-40 years | -1.05 | 0.54 | .444 | -2.64 - 0.54 |
|  |  | 26-30 years - 41-50 years | -1.52 | 0.60 | .147 | -3.29 - 0.25 |
|  |  | 26-30 years - 51-60 years | -4.18^*^ | 0.69 | .000 | -6.21 - -2.15 |
|  |  | 26-30 years - 61-70 years | -6.08^*^ | 1.07 | .000 | -9.24 - -2.91 |
|  |  | 26-30 years - >71 years | -5.10 | 4.65 | .929 | -18.82 - 8.62 |
|  |  | 31-40 years - <26 years | 0.95 | 2.19 | .999 | -5.53 - 7.43 |
|  |  | 31-40 years - 26-30 years | 1.05 | 0.54 | .444 | -0.54 - 2.64 |
|  |  | 31-40 years - 41-50 years | -0.47 | 0.45 | .945 | -1.8 - 0.86 |
|  |  | 31-40 years - 51-60 years | -3.13^*^ | 0.56 | .000 | -4.79 - -1.47 |
|  |  | 31-40 years - 61-70 years | -5.03^*^ | 1.00 | .000 | -7.97 - -2.08 |
|  |  | 31-40 years - >71 years | -4.05 | 4.63 | .976 | -17.72 - 9.63 |
|  |  | 41-50 years - <26 years | 1.42 | 2.21 | .995 | -5.11 - 7.95 |
|  |  | 41-50 years - 26-30 years | 1.52 | 0.60 | .147 | -0.25 - 3.29 |
|  |  | 41-50 years - 31-40 years | 0.47 | 0.45 | .945 | -0.86 - 1.8 |
|  |  | 41-50 years - 51-60 years | -2.66^*^ | 0.62 | .000 | -4.5 - -0.82 |
|  |  | 41-50 years - 61-70 years | -4.56^*^ | 1.03 | .000 | -7.61 - -1.51 |
|  |  | 41-50 years - >71 years | -3.58 | 4.64 | .988 | -17.28 - 10.12 |
|  |  | 51-60 years - <26 years | 4.08 | 2.24 | .531 | -2.52 - 10.68 |
|  |  | 51-60 years - 26-30 years | 4.18^*^ | 0.69 | .000 | 2.15 - 6.21 |
|  |  | 51-60 years - 31-40 years | 3.13^*^ | 0.56 | .000 | 1.47 - 4.79 |
|  |  | 51-60 years - 41-50 years | 2.66^*^ | 0.62 | .000 | 0.82 - 4.5 |
|  |  | 51-60 years - 61-70 years | -1.90 | 1.09 | .585 | -5.1 - 1.31 |
|  |  | 51-60 years - >71 years | -0.92 | 4.65 | 1.000 | -14.65 - 12.81 |
|  |  | 61-70 years - <26 years | 5.98 | 2.38 | .157 | -1.06 - 13.02 |
|  |  | 61-70 years - 26-30 years | 6.08^*^ | 1.07 | .000 | 2.91 - 9.24 |
|  |  | 61-70 years - 31-40 years | 5.03^*^ | 1.00 | .000 | 2.08 - 7.97 |
|  |  | 61-70 years - 41-50 years | 4.56^*^ | 1.03 | .000 | 1.51 - 7.61 |
|  |  | 61-70 years - 51-60 years | 1.90 | 1.09 | .585 | -1.31 - 5.1 |
|  |  | 61-70 years - >71 years | 0.98 | 4.72 | 1.000 | -12.97 - 14.92 |
|  |  | >7 years - <26 years | 5.00 | 5.11 | .959 | -10.09 - 20.09 |
|  |  | >7 years - 26-30 years | 5.10 | 4.65 | .929 | -8.62 - 18.82 |
|  |  | >7 years - 31-40 years | 4.05 | 4.63 | .976 | -9.63 - 17.72 |
|  |  | >7 years - 41-50 years | 3.58 | 4.64 | .988 | -10.12 - 17.28 |
|  |  | >7 years - 51-60 years | 0.92 | 4.65 | 1.000 | -12.81 - 14.65 |
|  |  | >7 years - 61-70 years | -0.98 | 4.72 | 1.000 | -14.92 - 12.97 |
|  | Marital Status | Not married without children - Not married with children | -2.979^*^ | 0.97 | .012 | -5.49 - -0.47 |
|  |  | Not married without children - Married without children | -0.08 | 0.68 | .999 | -1.84 - 1.68 |
|  |  | Not married without children - Married with children | -2.292^*^ | 0.47 | .000 | -3.5 - -1.09 |
|  |  | Not married with children - Not married without children | 2.979^*^ | 0.97 | .012 | 0.47 - 5.49 |
|  |  | Not married with children - Married without children | 2.901^*^ | 1.04 | .027 | 0.24 - 5.57 |
|  |  | Not married with children - Married with children | 0.69 | 0.91 | .874 | -1.65 - 3.02 |
|  |  | Married without children - Not married without children | 0.08 | 0.68 | .999 | -1.68 - 1.84 |
|  |  | Married without children - Not married with children | -2.901^*^ | 1.04 | .027 | -5.57 - -0.24 |
|  |  | Married without children - Married with children | -2.214^*^ | 0.59 | .001 | -3.72 - -0.71 |
|  |  | Married with children - Not married without children | 2.292^*^ | 0.47 | .000 | 1.09 - 3.5 |
|  |  | Married with children - Not married with children | -0.69 | 0.91 | .874 | -3.02 - 1.65 |
|  |  | Married with children - Married without children | 2.214^*^ | 0.59 | .001 | 0.71 - 3.72 |
|  | Academic Rank | Teaching staff - Lecturer | 0.13 | 0.54 | .999 | -1.36 - 1.62 |
|  |  | Teaching staff - Assistant professor | -1.82^*^ | 0.52 | .005 | -3.25 - -0.39 |
|  |  | Teaching staff - Associate professor | -3.99^*^ | 0.70 | .000 | -5.9 - -2.08 |
|  |  | Teaching staff - Professor | -6.20^*^ | 1.19 | .000 | -9.44 - -2.95 |
|  |  | Lecturer - Teaching staff | -0.13 | 0.54 | .999 | -1.62 - 1.36 |
|  |  | Lecturer - Assistant professor | -1.95^*^ | 0.42 | .000 | -3.09 - -0.8 |
|  |  | Lecturer - Associate professor | -4.12^*^ | 0.63 | .000 | -5.83 - -2.41 |
|  |  | Lecturer - Professor | -6.33^*^ | 1.14 | .000 | -9.45 - -3.2 |
|  |  | Assistant professor - Teaching staff | 1.82^*^ | 0.52 | .005 | 0.39 - 3.25 |
|  |  | Assistant professor - Lecturer | 1.95^*^ | 0.42 | .000 | 0.8 - 3.09 |
|  |  | Assistant professor - Associate professor | -2.17^*^ | 0.61 | .003 | -3.83 - -0.51 |
|  |  | Assistant professor - Professor | -4.38^*^ | 1.13 | .001 | -7.48 - -1.28 |
|  |  | Associate professor - Teaching staff | 3.99^*^ | 0.70 | .000 | 2.08 - 5.9 |
|  |  | Associate professor - Lecturer | 4.12^*^ | 0.63 | .000 | 2.41 - 5.83 |
|  |  | Associate professor - Assistant professor | 2.17^*^ | 0.61 | .003 | 0.51 - 3.83 |
|  |  | Associate professor - Professor | -2.21 | 1.23 | .374 | -5.56 - 1.14 |
|  |  | Professor - Teaching staff | 6.20^*^ | 1.19 | .000 | 2.95 - 9.44 |
|  |  | Professor - Lecturer | 6.33^*^ | 1.14 | .000 | 3.2 - 9.45 |
|  |  | Professor - Assistant professor | 4.38^*^ | 1.13 | .001 | 1.28 - 7.48 |
|  |  | Professor - Associate professor | 2.21 | 1.23 | .374 | -1.14 - 5.56 |
|  | Income | < Rp 3,000,000 - Rp 3,000,000 - Rp 4,999,999 | -0.84 | 0.54 | .524 | -2.3 - 0.63 |
|  |  | < Rp 3,000,000 - Rp 5,000,000 - Rp 9,999,999 | -1.60^*^ | 0.53 | .022 | -3.04 - -0.15 |
|  |  | < Rp 3,000,000 - Rp 10,000,000 - Rp 19,999,999 | -3.25^*^ | 0.62 | .000 | -4.96 - -1.55 |
|  |  | < Rp 3,000,000 - Rp 20,000,000 - Rp 49,999,999 | -4.96^*^ | 0.96 | .000 | -7.59 - -2.32 |
|  |  | Rp 3,000,000 - Rp 4,999,999 - < Rp 3,000,000 | 0.84 | 0.54 | .524 | -0.63 - 2.3 |
|  |  | Rp 3,000,000 - Rp 4,999,999 - Rp 5,000,000 - Rp 9,999,999 | -0.76 | 0.44 | .430 | -1.97 - 0.46 |
|  |  | Rp 3,000,000 - Rp 4,999,999 - Rp 10,000,000 - Rp 19,999,999 | -2.42^*^ | 0.56 | .000 | -3.93 - -0.9 |
|  |  | Rp 3,000,000 - Rp 4,999,999 - Rp 20,000,000 - Rp 49,999,999 | -4.12^*^ | 0.92 | .000 | -6.64 - -1.6 |
|  |  | Rp 5,000,000 - Rp 9,999,999 - < Rp 3,000,000 | 1.60^*^ | 0.53 | .022 | 0.15 - 3.04 |
|  |  | Rp 5,000,000 - Rp 9,999,999 - Rp 3,000,000 - Rp 4,999,999 | 0.76 | 0.44 | .430 | -0.46 - 1.97 |
|  |  | Rp 5,000,000 - Rp 9,999,999 - Rp 10,000,000 - Rp 19,999,999 | -1.66^*^ | 0.55 | .021 | -3.15 - -0.16 |
|  |  | Rp 5,000,000 - Rp 9,999,999 - Rp 20,000,000 - Rp 49,999,999 | -3.36^*^ | 0.92 | .002 | -5.86 - -0.86 |
|  |  | Rp 10,000,000 - Rp 19,999,999 - < Rp 3,000,000 | 3.25^*^ | 0.62 | .000 | 1.55 - 4.96 |
|  |  | Rp 10,000,000 - Rp 19,999,999 - Rp 3,000,000 - Rp 4,999,999 | 2.42^*^ | 0.56 | .000 | 0.9 - 3.93 |
|  |  | Rp 10,000,000 - Rp 19,999,999 - Rp 5,000,000 - Rp 9,999,999 | 1.66^*^ | 0.55 | .021 | 0.16 - 3.15 |
|  |  | Rp 10,000,000 - Rp 19,999,999 - Rp 20,000,000 - Rp 49,999,999 | -1.70 | 0.97 | .405 | -4.37 - 0.96 |
|  |  | Rp 20,000,000 - Rp 49,999,999 - < Rp 3,000,000 | 4.96^*^ | 0.96 | .000 | 2.32 - 7.59 |
|  |  | Rp 20,000,000 - Rp 49,999,999 - Rp 3,000,000 - Rp 4,999,999 | 4.12^*^ | 0.92 | .000 | 1.6 - 6.64 |
|  |  | Rp 20,000,000 - Rp 49,999,999 - Rp 5,000,000 - Rp 9,999,999 | 3.36^*^ | 0.92 | .002 | 0.86 - 5.86 |
|  |  | Rp 20,000,000 - Rp 49,999,999 - Rp 10,000,000 - Rp 19,999,999 | 1.70 | 0.97 | .405 | -0.96 - 4.37 |
| Happiness at Work | Age Group | <26 years - 26-30 years | 0.85 | 1.85 | .999 | -4.61 - 6.3 |
|  |  | <26 years - 31-40 years | -0.47 | 1.82 | 1.000 | -5.83 - 4.9 |
|  |  | <26 years - 41-50 years | -1.26 | 1.83 | .993 | -6.67 - 4.15 |
|  |  | <26 years - 51-60 years | -3.18 | 1.85 | .607 | -8.65 - 2.29 |
|  |  | <26 years - 61-70 years | -4.13 | 1.97 | .359 | -9.96 - 1.7 |
|  |  | <26 years - >71 years | -3.28 | 4.23 | .987 | -15.78 - 9.23 |
|  |  | 26-30 years - <26 years | -0.85 | 1.85 | .999 | -6.3 - 4.61 |
|  |  | 26-30 years - 31-40 years | -1.32^*^ | 0.44 | .049 | -2.63 - 0 |
|  |  | 26-30 years - 41-50 years | -2.11^*^ | 0.50 | .000 | -3.57 - -0.64 |
|  |  | 26-30 years - 51-60 years | -4.03^*^ | 0.57 | .000 | -5.7 - -2.34 |
|  |  | 26-30 years - 61-70 years | -4.98^*^ | 0.89 | .000 | -7.6 - -2.35 |
|  |  | 26-30 years - >71 years | -4.13 | 3.85 | .936 | -15.5 - 7.24 |
|  |  | 31-40 years - <26 years | 0.47 | 1.82 | 1.000 | -4.9 - 5.83 |
|  |  | 31-40 years - 26-30 years | 1.32^*^ | 0.44 | .049 | 0 - 2.63 |
|  |  | 31-40 years - 41-50 years | -0.79 | 0.37 | .341 | -1.9 - 0.31 |
|  |  | 31-40 years - 51-60 years | -2.71^*^ | 0.47 | .000 | -4.09 - -1.33 |
|  |  | 31-40 years - 61-70 years | -3.66^*^ | 0.83 | .000 | -6.1 - -1.22 |
|  |  | 31-40 years - >71 years | -2.81 | 3.84 | .991 | -14.14 - 8.52 |
|  |  | 41-50 years - <26 years | 1.26 | 1.83 | .993 | -4.15 - 6.67 |
|  |  | 41-50 years - 26-30 years | 2.11^*^ | 0.50 | .000 | 0.64 - 3.57 |
|  |  | 41-50 years - 31-40 years | 0.79 | 0.37 | .341 | -0.31 - 1.9 |
|  |  | 41-50 years - 51-60 years | -1.92^*^ | 0.52 | .004 | -3.44 - -0.39 |
|  |  | 41-50 years - 61-70 years | -2.87^*^ | 0.86 | .014 | -5.39 - -0.34 |
|  |  | 41-50 years - >71 years | -2.02 | 3.84 | .998 | -13.37 - 9.33 |
|  |  | 51-60 years - <26 years | 3.18 | 1.85 | .607 | -2.29 - 8.65 |
|  |  | 51-60 years - 26-30 years | 4.03^*^ | 0.57 | .000 | 2.34 - 5.7 |
|  |  | 51-60 years - 31-40 years | 2.71^*^ | 0.47 | .000 | 1.33 - 4.09 |
|  |  | 51-60 years - 41-50 years | 1.92^*^ | 0.52 | .004 | 0.39 - 3.44 |
|  |  | 51-60 years - 61-70 years | -0.95 | 0.90 | .941 | -3.61 - 1.71 |
|  |  | 51-60 years - >71 years | -0.10 | 3.85 | 1.000 | -11.48 - 11.27 |
|  |  | 61-70 years - <26 years | 4.13 | 1.97 | .359 | -1.7 - 9.96 |
|  |  | 61-70 years - 26-30 years | 4.98^*^ | 0.89 | .000 | 2.35 - 7.6 |
|  |  | 61-70 years - 31-40 years | 3.66^*^ | 0.83 | .000 | 1.22 - 6.1 |
|  |  | 61-70 years - 41-50 years | 2.87^*^ | 0.86 | .014 | 0.34 - 5.39 |
|  |  | 61-70 years - 51-60 years | 0.95 | 0.90 | .941 | -1.71 - 3.61 |
|  |  | 61-70 years - >71 years | 0.85 | 3.91 | 1.000 | -10.71 - 12.4 |
|  |  | >7 years - <26 years | 3.28 | 4.23 | .987 | -9.23 - 15.78 |
|  |  | >7 years - 26-30 years | 4.13 | 3.85 | .936 | -7.24 - 15.5 |
|  |  | >7 years - 31-40 years | 2.81 | 3.84 | .991 | -8.52 - 14.14 |
|  |  | >7 years - 41-50 years | 2.02 | 3.84 | .998 | -9.33 - 13.37 |
|  |  | >7 years - 51-60 years | 0.10 | 3.85 | 1.000 | -11.27 - 11.48 |
|  |  | >7 years - 61-70 years | -0.85 | 3.91 | 1.000 | -12.4 - 10.71 |
|  | Marital Status | Not married without children - Not married with children | -3.006^*^ | 0.81 | .001 | -5.08 - -0.93 |
|  |  | Not married without children - Married without children | -0.43 | 0.57 | .874 | -1.89 - 1.03 |
|  |  | Not married without children - Married with children | -2.155^*^ | 0.39 | .000 | -3.15 - -1.16 |
|  |  | Not married with children - Not married without children | 3.006^*^ | 0.81 | .001 | 0.93 - 5.08 |
|  |  | Not married with children - Married without children | 2.577^*^ | 0.86 | .015 | 0.37 - 4.79 |
|  |  | Not married with children - Married with children | 0.85 | 0.75 | .671 | -1.09 - 2.79 |
|  |  | Married without children - Not married without children | 0.43 | 0.57 | .874 | -1.03 - 1.89 |
|  |  | Married without children - Not married with children | -2.577^*^ | 0.86 | .015 | -4.79 - -0.37 |
|  |  | Married without children - Married with children | -1.726^*^ | 0.49 | .002 | -2.98 - -0.48 |
|  |  | Married with children - Not married without children | 2.155^*^ | 0.39 | .000 | 1.16 - 3.15 |
|  |  | Married with children - Not married with children | -0.85 | 0.75 | .671 | -2.79 - 1.09 |
|  |  | Married with children - Married without children | 1.726^*^ | 0.49 | .002 | 0.48 - 2.98 |
|  | Academic Rank | Teaching staff - Lecturer | 0.28 | 0.45 | .972 | -0.96 - 1.52 |
|  |  | Teaching staff - Assistant professor | -0.98 | 0.44 | .161 | -2.18 - 0.21 |
|  |  | Teaching staff - Associate professor | -3.08^*^ | 0.58 | .000 | -4.68 - -1.49 |
|  |  | Teaching staff - Professor | -4.34^*^ | 0.99 | .000 | -7.05 - -1.64 |
|  |  | Lecturer - Teaching staff | -0.28 | 0.45 | .972 | -1.52 - 0.96 |
|  |  | Lecturer - Assistant professor | -1.27^*^ | 0.35 | .003 | -2.22 - -0.31 |
|  |  | Lecturer - Associate professor | -3.36^*^ | 0.52 | .000 | -4.79 - -1.94 |
|  |  | Lecturer - Professor | -4.62^*^ | 0.96 | .000 | -7.23 - -2.01 |
|  |  | Assistant professor - Teaching staff | 0.98 | 0.44 | .161 | -0.21 - 2.18 |
|  |  | Assistant professor - Lecturer | 1.27^*^ | 0.35 | .003 | 0.31 - 2.22 |
|  |  | Assistant professor - Associate professor | -2.10^*^ | 0.51 | .000 | -3.48 - -0.71 |
|  |  | Assistant professor - Professor | -3.36^*^ | 0.95 | .004 | -5.95 - -0.77 |
|  |  | Associate professor - Teaching staff | 3.08^*^ | 0.58 | .000 | 1.49 - 4.68 |
|  |  | Associate professor - Lecturer | 3.36^*^ | 0.52 | .000 | 1.94 - 4.79 |
|  |  | Associate professor - Assistant professor | 2.10^*^ | 0.51 | .000 | 0.71 - 3.48 |
|  |  | Associate professor - Professor | -1.26 | 1.02 | .733 | -4.06 - 1.54 |
|  |  | Professor - Teaching staff | 4.34^*^ | 0.99 | .000 | 1.64 - 7.05 |
|  |  | Professor - Lecturer | 4.62^*^ | 0.96 | .000 | 2.01 - 7.23 |
|  |  | Professor - Assistant professor | 3.36^*^ | 0.95 | .004 | 0.77 - 5.95 |
|  |  | Professor - Associate professor | 1.26 | 1.02 | .733 | -1.54 - 4.06 |
|  | Income | < Rp 3,000,000 - Rp 3,000,000 - Rp 4,999,999 | -0.19 | 0.45 | .993 | -1.41 - 1.03 |
|  |  | < Rp 3,000,000 - Rp 5,000,000 - Rp 9,999,999 | -1.19 | 0.44 | .054 | -2.39 - 0.01 |
|  |  | < Rp 3,000,000 - Rp 10,000,000 - Rp 19,999,999 | -2.44^*^ | 0.52 | .000 | -3.86 - -1.02 |
|  |  | < Rp 3,000,000 - Rp 20,000,000 - Rp 49,999,999 | -3.33^*^ | 0.80 | .000 | -5.52 - -1.13 |
|  |  | Rp 3,000,000 - Rp 4,999,999 - < Rp 3,000,000 | 0.19 | 0.45 | .993 | -1.03 - 1.41 |
|  |  | Rp 3,000,000 - Rp 4,999,999 - Rp 5,000,000 - Rp 9,999,999 | -1.00 | 0.37 | .056 | -2.01 - 0.01 |
|  |  | Rp 3,000,000 - Rp 4,999,999 - Rp 10,000,000 - Rp 19,999,999 | -2.25^*^ | 0.46 | .000 | -3.51 - -0.99 |
|  |  | Rp 3,000,000 - Rp 4,999,999 - Rp 20,000,000 - Rp 49,999,999 | -3.13^*^ | 0.77 | .000 | -5.23 - -1.04 |
|  |  | Rp 5,000,000 - Rp 9,999,999 - < Rp 3,000,000 | 1.19 | 0.44 | .054 | -0.01 - 2.39 |
|  |  | Rp 5,000,000 - Rp 9,999,999 - Rp 3,000,000 - Rp 4,999,999 | 1.00 | 0.37 | .056 | -0.01 - 2.01 |
|  |  | Rp 5,000,000 - Rp 9,999,999 - Rp 10,000,000 - Rp 19,999,999 | -1.25^*^ | 0.46 | .047 | -2.5 - -0.01 |
|  |  | Rp 5,000,000 - Rp 9,999,999 - Rp 20,000,000 - Rp 49,999,999 | -2.14^*^ | 0.76 | .041 | -4.22 - -0.05 |
|  |  | Rp 10,000,000 - Rp 19,999,999 - < Rp 3,000,000 | 2.44^*^ | 0.52 | .000 | 1.02 - 3.86 |
|  |  | Rp 10,000,000 - Rp 19,999,999 - Rp 3,000,000 - Rp 4,999,999 | 2.25^*^ | 0.46 | .000 | 0.99 - 3.51 |
|  |  | Rp 10,000,000 - Rp 19,999,999 - Rp 5,000,000 - Rp 9,999,999 | 1.25^*^ | 0.46 | .047 | 0.01 - 2.5 |
|  |  | Rp 10,000,000 - Rp 19,999,999 - Rp 20,000,000 - Rp 49,999,999 | -0.88 | 0.81 | .812 | -3.1 - 1.33 |
|  |  | Rp 20,000,000 - Rp 49,999,999 - < Rp 3,000,000 | 3.33^*^ | 0.80 | .000 | 1.13 - 5.52 |
|  |  | Rp 20,000,000 - Rp 49,999,999 - Rp 3,000,000 - Rp 4,999,999 | 3.13^*^ | 0.77 | .000 | 1.04 - 5.23 |
|  |  | Rp 20,000,000 - Rp 49,999,999 - Rp 5,000,000 - Rp 9,999,999 | 2.14^*^ | 0.76 | .041 | 0.05 - 4.22 |
|  |  | Rp 20,000,000 - Rp 49,999,999 - Rp 10,000,000 - Rp 19,999,999 | 0.88 | 0.81 | .812 | -1.33 - 3.1 |
| Stress | Age Group | <26 years - 26-30 years | 0.52 | 2.11 | 1.000 | -5.72 - 6.75 |
|  |  | <26 years - 31-40 years | 1.08 | 2.08 | .999 | -5.06 - 7.21 |
|  |  | <26 years - 41-50 years | 2.91 | 2.09 | .806 | -3.27 - 9.09 |
|  |  | <26 years - 51-60 years | 5.09 | 2.12 | .196 | -1.16 - 11.34 |
|  |  | <26 years - 61-70 years | 7.35^*^ | 2.26 | .020 | 0.69 - 14.01 |
|  |  | <26 years - >71 years | 10.11 | 4.84 | .359 | -4.17 - 24.4 |
|  |  | 26-30 years - <26 years | -0.52 | 2.11 | 1.000 | -6.75 - 5.72 |
|  |  | 26-30 years - 31-40 years | 0.56 | 0.51 | .926 | -0.94 - 2.06 |
|  |  | 26-30 years - 41-50 years | 2.40^*^ | 0.57 | .000 | 0.72 - 4.07 |
|  |  | 26-30 years - 51-60 years | 4.58^*^ | 0.65 | .000 | 2.66 - 6.5 |
|  |  | 26-30 years - 61-70 years | 6.84^*^ | 1.02 | .000 | 3.84 - 9.83 |
|  |  | 26-30 years - >71 years | 9.60 | 4.40 | .306 | -3.39 - 22.58 |
|  |  | 31-40 years - <26 years | -1.08 | 2.08 | .999 | -7.21 - 5.06 |
|  |  | 31-40 years - 26-30 years | -0.56 | 0.51 | .926 | -2.06 - 0.94 |
|  |  | 31-40 years - 41-50 years | 1.83^*^ | 0.43 | .000 | 0.57 - 3.1 |
|  |  | 31-40 years - 51-60 years | 4.02^*^ | 0.53 | .000 | 2.44 - 5.59 |
|  |  | 31-40 years - 61-70 years | 6.27^*^ | 0.94 | .000 | 3.48 - 9.06 |
|  |  | 31-40 years - >71 years | 9.03 | 4.38 | .377 | -3.91 - 21.97 |
|  |  | 41-50 years - <26 years | -2.91 | 2.09 | .806 | -9.09 - 3.27 |
|  |  | 41-50 years - 26-30 years | -2.40^*^ | 0.57 | .000 | -4.07 - -0.72 |
|  |  | 41-50 years - 31-40 years | -1.83^*^ | 0.43 | .000 | -3.1 - -0.57 |
|  |  | 41-50 years - 51-60 years | 2.18^*^ | 0.59 | .004 | 0.44 - 3.92 |
|  |  | 41-50 years - 61-70 years | 4.44^*^ | 0.98 | .000 | 1.55 - 7.32 |
|  |  | 41-50 years - >71 years | 7.20 | 4.39 | .656 | -5.76 - 20.16 |
|  |  | 51-60 years - <26 years | -5.09 | 2.12 | .196 | -11.34 - 1.16 |
|  |  | 51-60 years - 26-30 years | -4.58^*^ | 0.65 | .000 | -6.5 - -2.66 |
|  |  | 51-60 years - 31-40 years | -4.02^*^ | 0.53 | .000 | -5.59 - -2.44 |
|  |  | 51-60 years - 41-50 years | -2.18^*^ | 0.59 | .004 | -3.92 - -0.44 |
|  |  | 51-60 years - 61-70 years | 2.26 | 1.03 | .299 | -0.78 - 5.29 |
|  |  | 51-60 years - >71 years | 5.02 | 4.40 | .916 | -7.98 - 18.01 |
|  |  | 61-70 years - <26 years | -7.35^*^ | 2.26 | .020 | -14.01 - -0.69 |
|  |  | 61-70 years - 26-30 years | -6.84^*^ | 1.02 | .000 | -9.83 - -3.84 |
|  |  | 61-70 years - 31-40 years | -6.27^*^ | 0.94 | .000 | -9.06 - -3.48 |
|  |  | 61-70 years - 41-50 years | -4.44^*^ | 0.98 | .000 | -7.32 - -1.55 |
|  |  | 61-70 years - 51-60 years | -2.26 | 1.03 | .299 | -5.29 - 0.78 |
|  |  | 61-70 years - >71 years | 2.76 | 4.47 | .996 | -10.44 - 15.96 |
|  |  | >7 years - <26 years | -10.11 | 4.84 | .359 | -24.4 - 4.17 |
|  |  | >7 years - 26-30 years | -9.60 | 4.40 | .306 | -22.58 - 3.39 |
|  |  | >7 years - 31-40 years | -9.03 | 4.38 | .377 | -21.97 - 3.91 |
|  |  | >7 years - 41-50 years | -7.20 | 4.39 | .656 | -20.16 - 5.76 |
|  |  | >7 years - 51-60 years | -5.02 | 4.40 | .916 | -18.01 - 7.98 |
|  |  | >7 years - 61-70 years | -2.76 | 4.47 | .996 | -15.96 - 10.44 |
|  | Marital Status | Not married without children - Not married with children | 3.234^*^ | 0.94 | .003 | 0.82 - 5.65 |
|  |  | Not married without children - Married without children | 0.37 | 0.66 | .942 | -1.32 - 2.07 |
|  |  | Not married without children - Married with children | 2.067^*^ | 0.45 | .000 | 0.91 - 3.23 |
|  |  | Not married with children - Not married without children | -3.234^*^ | 0.94 | .003 | -5.65 - -0.82 |
|  |  | Not married with children - Married without children | -2.860^*^ | 1.00 | .022 | -5.43 - -0.29 |
|  |  | Not married with children - Married with children | -1.17 | 0.88 | .542 | -3.42 - 1.08 |
|  |  | Married without children - Not married without children | -0.37 | 0.66 | .942 | -2.07 - 1.32 |
|  |  | Married without children - Not married with children | 2.860^*^ | 1.00 | .022 | 0.29 - 5.43 |
|  |  | Married without children - Married with children | 1.692^*^ | 0.56 | .015 | 0.24 - 3.15 |
|  |  | Married with children - Not married without children | -2.067^*^ | 0.45 | .000 | -3.23 - -0.91 |
|  |  | Married with children - Not married with children | 1.17 | 0.88 | .542 | -1.08 - 3.42 |
|  |  | Married with children - Married without children | -1.692^*^ | 0.56 | .015 | -3.15 - -0.24 |
|  | Academic Rank | Teaching staff - Lecturer | -0.37 | 0.53 | .955 | -1.82 - 1.07 |
|  |  | Teaching staff - Assistant professor | 0.45 | 0.51 | .905 | -0.94 - 1.84 |
|  |  | Teaching staff - Associate professor | 2.65^*^ | 0.68 | .001 | 0.79 - 4.51 |
|  |  | Teaching staff - Professor | 5.34^*^ | 1.15 | .000 | 2.19 - 8.49 |
|  |  | Lecturer - Teaching staff | 0.37 | 0.53 | .955 | -1.07 - 1.82 |
|  |  | Lecturer - Assistant professor | 0.82 | 0.41 | .259 | -0.29 - 1.93 |
|  |  | Lecturer - Associate professor | 3.02^*^ | 0.61 | .000 | 1.36 - 4.68 |
|  |  | Lecturer - Professor | 5.72^*^ | 1.11 | .000 | 2.68 - 8.75 |
|  |  | Assistant professor - Teaching staff | -0.45 | 0.51 | .905 | -1.84 - 0.94 |
|  |  | Assistant professor - Lecturer | -0.82 | 0.41 | .259 | -1.93 - 0.29 |
|  |  | Assistant professor - Associate professor | 2.20^*^ | 0.59 | .002 | 0.59 - 3.81 |
|  |  | Assistant professor - Professor | 4.90^*^ | 1.10 | .000 | 1.88 - 7.91 |
|  |  | Associate professor - Teaching staff | -2.65^*^ | 0.68 | .001 | -4.51 - -0.79 |
|  |  | Associate professor - Lecturer | -3.02^*^ | 0.61 | .000 | -4.68 - -1.36 |
|  |  | Associate professor - Assistant professor | -2.20^*^ | 0.59 | .002 | -3.81 - -0.59 |
|  |  | Associate professor - Professor | 2.69 | 1.19 | .159 | -0.56 - 5.95 |
|  |  | Professor - Teaching staff | -5.34^*^ | 1.15 | .000 | -8.49 - -2.19 |
|  |  | Professor - Lecturer | -5.72^*^ | 1.11 | .000 | -8.75 - -2.68 |
|  |  | Professor - Assistant professor | -4.90^*^ | 1.10 | .000 | -7.91 - -1.88 |
|  |  | Professor - Associate professor | -2.69 | 1.19 | .159 | -5.95 - 0.56 |
|  | Income | < Rp 3,000,000 - Rp 3,000,000 - Rp 4,999,999 | 0.24 | 0.51 | .990 | -1.16 - 1.65 |
|  |  | < Rp 3,000,000 - Rp 5,000,000 - Rp 9,999,999 | 0.12 | 0.51 | .999 | -1.27 - 1.5 |
|  |  | < Rp 3,000,000 - Rp 10,000,000 - Rp 19,999,999 | 3.01^*^ | 0.60 | .000 | 1.38 - 4.65 |
|  |  | < Rp 3,000,000 - Rp 20,000,000 - Rp 49,999,999 | 4.40^*^ | 0.92 | .000 | 1.88 - 6.93 |
|  |  | Rp 3,000,000 - Rp 4,999,999 - < Rp 3,000,000 | -0.24 | 0.51 | .990 | -1.65 - 1.16 |
|  |  | Rp 3,000,000 - Rp 4,999,999 - Rp 5,000,000 - Rp 9,999,999 | -0.13 | 0.43 | .998 | -1.29 - 1.04 |
|  |  | Rp 3,000,000 - Rp 4,999,999 - Rp 10,000,000 - Rp 19,999,999 | 2.77^*^ | 0.53 | .000 | 1.32 - 4.22 |
|  |  | Rp 3,000,000 - Rp 4,999,999 - Rp 20,000,000 - Rp 49,999,999 | 4.16^*^ | 0.88 | .000 | 1.75 - 6.57 |
|  |  | Rp 5,000,000 - Rp 9,999,999 - < Rp 3,000,000 | -0.12 | 0.51 | .999 | -1.5 - 1.27 |
|  |  | Rp 5,000,000 - Rp 9,999,999 - Rp 3,000,000 - Rp 4,999,999 | 0.13 | 0.43 | .998 | -1.04 - 1.29 |
|  |  | Rp 5,000,000 - Rp 9,999,999 - Rp 10,000,000 - Rp 19,999,999 | 2.90^*^ | 0.52 | .000 | 1.46 - 4.33 |
|  |  | Rp 5,000,000 - Rp 9,999,999 - Rp 20,000,000 - Rp 49,999,999 | 4.29^*^ | 0.88 | .000 | 1.89 - 6.69 |
|  |  | Rp 10,000,000 - Rp 19,999,999 - < Rp 3,000,000 | -3.01^*^ | 0.60 | .000 | -4.65 - -1.38 |
|  |  | Rp 10,000,000 - Rp 19,999,999 - Rp 3,000,000 - Rp 4,999,999 | -2.77^*^ | 0.53 | .000 | -4.22 - -1.32 |
|  |  | Rp 10,000,000 - Rp 19,999,999 - Rp 5,000,000 - Rp 9,999,999 | -2.90^*^ | 0.52 | .000 | -4.33 - -1.46 |
|  |  | Rp 10,000,000 - Rp 19,999,999 - Rp 20,000,000 - Rp 49,999,999 | 1.39 | 0.93 | .571 | -1.16 - 3.94 |
|  |  | Rp 20,000,000 - Rp 49,999,999 - < Rp 3,000,000 | -4.40^*^ | 0.92 | .000 | -6.93 - -1.88 |
|  |  | Rp 20,000,000 - Rp 49,999,999 - Rp 3,000,000 - Rp 4,999,999 | -4.16^*^ | 0.88 | .000 | -6.57 - -1.75 |
|  |  | Rp 20,000,000 - Rp 49,999,999 - Rp 5,000,000 - Rp 9,999,999 | -4.29^*^ | 0.88 | .000 | -6.69 - -1.89 |
|  |  | Rp 20,000,000 - Rp 49,999,999 - Rp 10,000,000 - Rp 19,999,999 | -1.39 | 0.93 | .571 | -3.94 - 1.16 |
| Anxiety | Age Group | <26 years - 26-30 years | 1.33 | 1.41 | .965 | -2.82 - 5.48 |
|  |  | <26 years - 31-40 years | 1.69 | 1.38 | .885 | -2.39 - 5.78 |
|  |  | <26 years - 41-50 years | 2.47 | 1.39 | .568 | -1.65 - 6.58 |
|  |  | <26 years - 51-60 years | 4.08 | 1.41 | .059 | -0.08 - 8.24 |
|  |  | <26 years - 61-70 years | 5.05^*^ | 1.50 | .014 | 0.61 - 9.48 |
|  |  | <26 years - >71 years | 5.22 | 3.22 | .669 | -4.29 - 14.74 |
|  |  | 26-30 years - <26 years | -1.33 | 1.41 | .965 | -5.48 - 2.82 |
|  |  | 26-30 years - 31-40 years | 0.36 | 0.34 | .937 | -0.64 - 1.36 |
|  |  | 26-30 years - 41-50 years | 1.14^*^ | 0.38 | .042 | 0.02 - 2.25 |
|  |  | 26-30 years - 51-60 years | 2.75^*^ | 0.43 | .000 | 1.47 - 4.03 |
|  |  | 26-30 years - 61-70 years | 3.72^*^ | 0.68 | .000 | 1.72 - 5.71 |
|  |  | 26-30 years - >71 years | 3.89 | 2.93 | .839 | -4.76 - 12.54 |
|  |  | 31-40 years - <26 years | -1.69 | 1.38 | .885 | -5.78 - 2.39 |
|  |  | 31-40 years - 26-30 years | -0.36 | 0.34 | .937 | -1.36 - 0.64 |
|  |  | 31-40 years - 41-50 years | 0.78 | 0.28 | .093 | -0.06 - 1.62 |
|  |  | 31-40 years - 51-60 years | 2.39^*^ | 0.35 | .000 | 1.34 - 3.44 |
|  |  | 31-40 years - 61-70 years | 3.36^*^ | 0.63 | .000 | 1.5 - 5.21 |
|  |  | 31-40 years - >71 years | 3.53 | 2.92 | .891 | -5.09 - 12.15 |
|  |  | 41-50 years - <26 years | -2.47 | 1.39 | .568 | -6.58 - 1.65 |
|  |  | 41-50 years - 26-30 years | -1.14^*^ | 0.38 | .042 | -2.25 - -0.02 |
|  |  | 41-50 years - 31-40 years | -0.78 | 0.28 | .093 | -1.62 - 0.06 |
|  |  | 41-50 years - 51-60 years | 1.61^*^ | 0.39 | .001 | 0.45 - 2.77 |
|  |  | 41-50 years - 61-70 years | 2.58^*^ | 0.65 | .002 | 0.66 - 4.5 |
|  |  | 41-50 years - >71 years | 2.75 | 2.92 | .966 | -5.88 - 11.39 |
|  |  | 51-60 years - <26 years | -4.08 | 1.41 | .059 | -8.24 - 0.08 |
|  |  | 51-60 years - 26-30 years | -2.75^*^ | 0.43 | .000 | -4.03 - -1.47 |
|  |  | 51-60 years - 31-40 years | -2.39^*^ | 0.35 | .000 | -3.44 - -1.34 |
|  |  | 51-60 years - 41-50 years | -1.61^*^ | 0.39 | .001 | -2.77 - -0.45 |
|  |  | 51-60 years - 61-70 years | 0.97 | 0.68 | .796 | -1.05 - 2.99 |
|  |  | 51-60 years - >71 years | 1.14 | 2.93 | 1.000 | -7.52 - 9.8 |
|  |  | 61-70 years - <26 years | -5.05^*^ | 1.50 | .014 | -9.48 - -0.61 |
|  |  | 61-70 years - 26-30 years | -3.72^*^ | 0.68 | .000 | -5.71 - -1.72 |
|  |  | 61-70 years - 31-40 years | -3.36^*^ | 0.63 | .000 | -5.21 - -1.5 |
|  |  | 61-70 years - 41-50 years | -2.58^*^ | 0.65 | .002 | -4.5 - -0.66 |
|  |  | 61-70 years - 51-60 years | -0.97 | 0.68 | .796 | -2.99 - 1.05 |
|  |  | 61-70 years - >71 years | 0.17 | 2.98 | 1.000 | -8.62 - 8.96 |
|  |  | >7 years - <26 years | -5.22 | 3.22 | .669 | -14.74 - 4.29 |
|  |  | >7 years - 26-30 years | -3.89 | 2.93 | .839 | -12.54 - 4.76 |
|  |  | >7 years - 31-40 years | -3.53 | 2.92 | .891 | -12.15 - 5.09 |
|  |  | >7 years - 41-50 years | -2.75 | 2.92 | .966 | -11.39 - 5.88 |
|  |  | >7 years - 51-60 years | -1.14 | 2.93 | 1.000 | -9.8 - 7.52 |
|  |  | >7 years - 61-70 years | -0.17 | 2.98 | 1.000 | -8.96 - 8.62 |
|  | Marital Status | Not married without children - Not married with children | 2.363^*^ | 0.62 | .001 | 0.77 - 3.95 |
|  |  | Not married without children - Married without children | 0.13 | 0.43 | .992 | -0.99 - 1.24 |
|  |  | Not married without children - Married with children | 1.375^*^ | 0.30 | .000 | 0.61 - 2.14 |
|  |  | Not married with children - Not married without children | -2.363^*^ | 0.62 | .001 | -3.95 - -0.77 |
|  |  | Not married with children - Married without children | -2.237^*^ | 0.66 | .004 | -3.93 - -0.55 |
|  |  | Not married with children - Married with children | -0.99 | 0.58 | .316 | -2.47 - 0.49 |
|  |  | Married without children - Not married without children | -0.13 | 0.43 | .992 | -1.24 - 0.99 |
|  |  | Married without children - Not married with children | 2.237^*^ | 0.66 | .004 | 0.55 - 3.93 |
|  |  | Married without children - Married with children | 1.250^*^ | 0.37 | .004 | 0.29 - 2.21 |
|  |  | Married with children - Not married without children | -1.375^*^ | 0.30 | .000 | -2.14 - -0.61 |
|  |  | Married with children - Not married with children | 0.99 | 0.58 | .316 | -0.49 - 2.47 |
|  |  | Married with children - Married without children | -1.250^*^ | 0.37 | .004 | -2.21 - -0.29 |
|  | Academic Rank | Teaching staff - Lecturer | -0.17 | 0.35 | .989 | -1.13 - 0.79 |
|  |  | Teaching staff - Assistant professor | 0.47 | 0.34 | .622 | -0.45 - 1.39 |
|  |  | Teaching staff - Associate professor | 1.67^*^ | 0.45 | .002 | 0.44 - 2.9 |
|  |  | Teaching staff - Professor | 2.11^*^ | 0.76 | .047 | 0.02 - 4.19 |
|  |  | Lecturer - Teaching staff | 0.17 | 0.35 | .989 | -0.79 - 1.13 |
|  |  | Lecturer - Assistant professor | 0.64 | 0.27 | .121 | -0.09 - 1.38 |
|  |  | Lecturer - Associate professor | 1.83^*^ | 0.40 | .000 | 0.73 - 2.93 |
|  |  | Lecturer - Professor | 2.27^*^ | 0.74 | .018 | 0.26 - 4.29 |
|  |  | Assistant professor - Teaching staff | -0.47 | 0.34 | .622 | -1.39 - 0.45 |
|  |  | Assistant professor - Lecturer | -0.64 | 0.27 | .121 | -1.38 - 0.09 |
|  |  | Assistant professor - Associate professor | 1.19^*^ | 0.39 | .020 | 0.12 - 2.26 |
|  |  | Assistant professor - Professor | 1.63 | 0.73 | .169 | -0.37 - 3.63 |
|  |  | Associate professor - Teaching staff | -1.67^*^ | 0.45 | .002 | -2.9 - -0.44 |
|  |  | Associate professor - Lecturer | -1.83^*^ | 0.40 | .000 | -2.93 - -0.73 |
|  |  | Associate professor - Assistant professor | -1.19^*^ | 0.39 | .020 | -2.26 - -0.12 |
|  |  | Associate professor - Professor | 0.44 | 0.79 | .981 | -1.72 - 2.6 |
|  |  | Professor - Teaching staff | -2.11^*^ | 0.76 | .047 | -4.19 - -0.02 |
|  |  | Professor - Lecturer | -2.27^*^ | 0.74 | .018 | -4.29 - -0.26 |
|  |  | Professor - Assistant professor | -1.63 | 0.73 | .169 | -3.63 - 0.37 |
|  |  | Professor - Associate professor | -0.44 | 0.79 | .981 | -2.6 - 1.72 |
|  | Income | < Rp 3,000,000 - Rp 3,000,000 - Rp 4,999,999 | 0.28 | 0.34 | .926 | -0.66 - 1.22 |
|  |  | < Rp 3,000,000 - Rp 5,000,000 - Rp 9,999,999 | 0.28 | 0.34 | .917 | -0.64 - 1.21 |
|  |  | < Rp 3,000,000 - Rp 10,000,000 - Rp 19,999,999 | 1.42^*^ | 0.40 | .003 | 0.33 - 2.51 |
|  |  | < Rp 3,000,000 - Rp 20,000,000 - Rp 49,999,999 | 1.62 | 0.62 | .067 | -0.07 - 3.3 |
|  |  | Rp 3,000,000 - Rp 4,999,999 - < Rp 3,000,000 | -0.28 | 0.34 | .926 | -1.22 - 0.66 |
|  |  | Rp 3,000,000 - Rp 4,999,999 - Rp 5,000,000 - Rp 9,999,999 | 0.00 | 0.28 | 1.000 | -0.77 - 0.78 |
|  |  | Rp 3,000,000 - Rp 4,999,999 - Rp 10,000,000 - Rp 19,999,999 | 1.14^*^ | 0.36 | .011 | 0.17 - 2.11 |
|  |  | Rp 3,000,000 - Rp 4,999,999 - Rp 20,000,000 - Rp 49,999,999 | 1.34 | 0.59 | .156 | -0.27 - 2.95 |
|  |  | Rp 5,000,000 - Rp 9,999,999 - < Rp 3,000,000 | -0.28 | 0.34 | .917 | -1.21 - 0.64 |
|  |  | Rp 5,000,000 - Rp 9,999,999 - Rp 3,000,000 - Rp 4,999,999 | 0.00 | 0.28 | 1.000 | -0.78 - 0.77 |
|  |  | Rp 5,000,000 - Rp 9,999,999 - Rp 10,000,000 - Rp 19,999,999 | 1.14^*^ | 0.35 | .010 | 0.18 - 2.09 |
|  |  | Rp 5,000,000 - Rp 9,999,999 - Rp 20,000,000 - Rp 49,999,999 | 1.33 | 0.59 | .154 | -0.27 - 2.93 |
|  |  | Rp 10,000,000 - Rp 19,999,999 - < Rp 3,000,000 | -1.42^*^ | 0.40 | .003 | -2.51 - -0.33 |
|  |  | Rp 10,000,000 - Rp 19,999,999 - Rp 3,000,000 - Rp 4,999,999 | -1.14^*^ | 0.36 | .011 | -2.11 - -0.17 |
|  |  | Rp 10,000,000 - Rp 19,999,999 - Rp 5,000,000 - Rp 9,999,999 | -1.14^*^ | 0.35 | .010 | -2.09 - -0.18 |
|  |  | Rp 10,000,000 - Rp 19,999,999 - Rp 20,000,000 - Rp 49,999,999 | 0.19 | 0.62 | .998 | -1.51 - 1.9 |
|  |  | Rp 20,000,000 - Rp 49,999,999 - < Rp 3,000,000 | -1.62 | 0.62 | .067 | -3.3 - 0.07 |
|  |  | Rp 20,000,000 - Rp 49,999,999 - Rp 3,000,000 - Rp 4,999,999 | -1.34 | 0.59 | .156 | -2.95 - 0.27 |
|  |  | Rp 20,000,000 - Rp 49,999,999 - Rp 5,000,000 - Rp 9,999,999 | -1.33 | 0.59 | .154 | -2.93 - 0.27 |
|  |  | Rp 20,000,000 - Rp 49,999,999 - Rp 10,000,000 - Rp 19,999,999 | -0.19 | 0.62 | .998 | -1.9 - 1.51 |
| Depression | Age Group | <26 years - 26-30 years | 0.86 | 1.47 | .997 | -3.48 - 5.19 |
|  |  | <26 years - 31-40 years | 1.40 | 1.45 | .961 | -2.87 - 5.67 |
|  |  | <26 years - 41-50 years | 2.81 | 1.46 | .462 | -1.49 - 7.1 |
|  |  | <26 years - 51-60 years | 4.42^*^ | 1.47 | .043 | 0.07 - 8.77 |
|  |  | <26 years - 61-70 years | 4.70^*^ | 1.57 | .044 | 0.06 - 9.33 |
|  |  | <26 years - >71 years | 4.61 | 3.37 | .818 | -5.33 - 14.55 |
|  |  | 26-30 years - <26 years | -0.86 | 1.47 | .997 | -5.19 - 3.48 |
|  |  | 26-30 years - 31-40 years | 0.54 | 0.35 | .724 | -0.5 - 1.59 |
|  |  | 26-30 years - 41-50 years | 1.95^*^ | 0.39 | .000 | 0.79 - 3.11 |
|  |  | 26-30 years - 51-60 years | 3.56^*^ | 0.45 | .000 | 2.23 - 4.9 |
|  |  | 26-30 years - 61-70 years | 3.84^*^ | 0.71 | .000 | 1.76 - 5.93 |
|  |  | 26-30 years - >71 years | 3.75 | 3.06 | .884 | -5.28 - 12.79 |
|  |  | 31-40 years - <26 years | -1.40 | 1.45 | .961 | -5.67 - 2.87 |
|  |  | 31-40 years - 26-30 years | -0.54 | 0.35 | .724 | -1.59 - 0.5 |
|  |  | 31-40 years - 41-50 years | 1.41^*^ | 0.30 | .000 | 0.53 - 2.28 |
|  |  | 31-40 years - 51-60 years | 3.02^*^ | 0.37 | .000 | 1.93 - 4.12 |
|  |  | 31-40 years - 61-70 years | 3.30^*^ | 0.66 | .000 | 1.36 - 5.24 |
|  |  | 31-40 years - >71 years | 3.21 | 3.05 | .941 | -5.79 - 12.21 |
|  |  | 41-50 years - <26 years | -2.81 | 1.46 | .462 | -7.1 - 1.49 |
|  |  | 41-50 years - 26-30 years | -1.95^*^ | 0.39 | .000 | -3.11 - -0.79 |
|  |  | 41-50 years - 31-40 years | -1.41^*^ | 0.30 | .000 | -2.28 - -0.53 |
|  |  | 41-50 years - 51-60 years | 1.61^*^ | 0.41 | .002 | 0.4 - 2.82 |
|  |  | 41-50 years - 61-70 years | 1.89 | 0.68 | .080 | -0.12 - 3.9 |
|  |  | 41-50 years - >71 years | 1.80 | 3.05 | .997 | -7.21 - 10.82 |
|  |  | 51-60 years - <26 years | -4.42^*^ | 1.47 | .043 | -8.77 - -0.07 |
|  |  | 51-60 years - 26-30 years | -3.56^*^ | 0.45 | .000 | -4.9 - -2.23 |
|  |  | 51-60 years - 31-40 years | -3.02^*^ | 0.37 | .000 | -4.12 - -1.93 |
|  |  | 51-60 years - 41-50 years | -1.61^*^ | 0.41 | .002 | -2.82 - -0.4 |
|  |  | 51-60 years - 61-70 years | 0.28 | 0.72 | 1.000 | -1.83 - 2.39 |
|  |  | 51-60 years - >71 years | 0.19 | 3.06 | 1.000 | -8.85 - 9.23 |
|  |  | 61-70 years - <26 years | -4.70^*^ | 1.57 | .044 | -9.33 - -0.06 |
|  |  | 61-70 years - 26-30 years | -3.84^*^ | 0.71 | .000 | -5.93 - -1.76 |
|  |  | 61-70 years - 31-40 years | -3.30^*^ | 0.66 | .000 | -5.24 - -1.36 |
|  |  | 61-70 years - 41-50 years | -1.89 | 0.68 | .080 | -3.9 - 0.12 |
|  |  | 61-70 years - 51-60 years | -0.28 | 0.72 | 1.000 | -2.39 - 1.83 |
|  |  | 61-70 years - >71 years | -0.09 | 3.11 | 1.000 | -9.27 - 9.1 |
|  |  | >7 years - <26 years | -4.61 | 3.37 | .818 | -14.55 - 5.33 |
|  |  | >7 years - 26-30 years | -3.75 | 3.06 | .884 | -12.79 - 5.28 |
|  |  | >7 years - 31-40 years | -3.21 | 3.05 | .941 | -12.21 - 5.79 |
|  |  | >7 years - 41-50 years | -1.80 | 3.05 | .997 | -10.82 - 7.21 |
|  |  | >7 years - 51-60 years | -0.19 | 3.06 | 1.000 | -9.23 - 8.85 |
|  |  | >7 years - 61-70 years | 0.09 | 3.11 | 1.000 | -9.1 - 9.27 |
|  | Marital Status | Not married without children - Not married with children | 2.41530^*^ | 0.65 | .001 | 0.74 - 4.09 |
|  |  | Not married without children - Married without children | 0.45 | 0.46 | .754 | -0.72 - 1.63 |
|  |  | Not married without children - Married with children | 1.80816^*^ | 0.31 | .000 | 1.01 - 2.61 |
|  |  | Not married with children - Not married without children | -2.41530^*^ | 0.65 | .001 | -4.09 - -0.74 |
|  |  | Not married with children - Married without children | -1.96259^*^ | 0.69 | .024 | -3.74 - -0.19 |
|  |  | Not married with children - Married with children | -0.61 | 0.61 | .748 | -2.16 - 0.95 |
|  |  | Married without children - Not married without children | -0.45 | 0.46 | .754 | -1.63 - 0.72 |
|  |  | Married without children - Not married with children | 1.96259^*^ | 0.69 | .024 | 0.19 - 3.74 |
|  |  | Married without children - Married with children | 1.35544^*^ | 0.39 | .003 | 0.35 - 2.36 |
|  |  | Married with children - Not married without children | -1.80816^*^ | 0.31 | .000 | -2.61 - -1.01 |
|  |  | Married with children - Not married with children | 0.61 | 0.61 | .748 | -0.95 - 2.16 |
|  |  | Married with children - Married without children | -1.35544^*^ | 0.39 | .003 | -2.36 - -0.35 |
|  | Academic Rank | Teaching staff - Lecturer | -0.08 | 0.37 | .999 | -1.09 - 0.92 |
|  |  | Teaching staff - Assistant professor | 0.88 | 0.35 | .095 | -0.09 - 1.84 |
|  |  | Teaching staff - Associate professor | 2.32^*^ | 0.47 | .000 | 1.03 - 3.61 |
|  |  | Teaching staff - Professor | 3.33^*^ | 0.80 | .000 | 1.14 - 5.51 |
|  |  | Lecturer - Teaching staff | 0.08 | 0.37 | .999 | -0.92 - 1.09 |
|  |  | Lecturer - Assistant professor | .96^*^ | 0.28 | .006 | 0.19 - 1.73 |
|  |  | Lecturer - Associate professor | 2.40^*^ | 0.42 | .000 | 1.25 - 3.55 |
|  |  | Lecturer - Professor | 3.41^*^ | 0.77 | .000 | 1.3 - 5.52 |
|  |  | Assistant professor - Teaching staff | -0.88 | 0.35 | .095 | -1.84 - 0.09 |
|  |  | Assistant professor - Lecturer | -.96^*^ | 0.28 | .006 | -1.73 - -0.19 |
|  |  | Assistant professor - Associate professor | 1.44^*^ | 0.41 | .004 | 0.32 - 2.56 |
|  |  | Assistant professor - Professor | 2.45^*^ | 0.76 | .012 | 0.36 - 4.54 |
|  |  | Associate professor - Teaching staff | -2.32^*^ | 0.47 | .000 | -3.61 - -1.03 |
|  |  | Associate professor - Lecturer | -2.40^*^ | 0.42 | .000 | -3.55 - -1.25 |
|  |  | Associate professor - Assistant professor | -1.44^*^ | 0.41 | .004 | -2.56 - -0.32 |
|  |  | Associate professor - Professor | 1.01 | 0.83 | .739 | -1.25 - 3.27 |
|  |  | Professor - Teaching staff | -3.33^*^ | 0.80 | .000 | -5.51 - -1.14 |
|  |  | Professor - Lecturer | -3.41^*^ | 0.77 | .000 | -5.52 - -1.3 |
|  |  | Professor - Assistant professor | -2.45^*^ | 0.76 | .012 | -4.54 - -0.36 |
|  |  | Professor - Associate professor | -1.01 | 0.83 | .739 | -3.27 - 1.25 |
|  | Income | < Rp 3,000,000 - Rp 3,000,000 - Rp 4,999,999 | -0.11 | 0.36 | .998 | -1.09 - 0.87 |
|  |  | < Rp 3,000,000 - Rp 5,000,000 - Rp 9,999,999 | 0.35 | 0.35 | .857 | -0.61 - 1.31 |
|  |  | < Rp 3,000,000 - Rp 10,000,000 - Rp 19,999,999 | 1.95^*^ | 0.42 | .000 | 0.81 - 3.09 |
|  |  | < Rp 3,000,000 - Rp 20,000,000 - Rp 49,999,999 | 2.82^*^ | 0.64 | .000 | 1.06 - 4.58 |
|  |  | Rp 3,000,000 - Rp 4,999,999 - < Rp 3,000,000 | 0.11 | 0.36 | .998 | -0.87 - 1.09 |
|  |  | Rp 3,000,000 - Rp 4,999,999 - Rp 5,000,000 - Rp 9,999,999 | 0.46 | 0.30 | .534 | -0.35 - 1.27 |
|  |  | Rp 3,000,000 - Rp 4,999,999 - Rp 10,000,000 - Rp 19,999,999 | 2.06^*^ | 0.37 | .000 | 1.05 - 3.07 |
|  |  | Rp 3,000,000 - Rp 4,999,999 - Rp 20,000,000 - Rp 49,999,999 | 2.93^*^ | 0.61 | .000 | 1.25 - 4.61 |
|  |  | Rp 5,000,000 - Rp 9,999,999 - < Rp 3,000,000 | -0.35 | 0.35 | .857 | -1.31 - 0.61 |
|  |  | Rp 5,000,000 - Rp 9,999,999 - Rp 3,000,000 - Rp 4,999,999 | -0.46 | 0.30 | .534 | -1.27 - 0.35 |
|  |  | Rp 5,000,000 - Rp 9,999,999 - Rp 10,000,000 - Rp 19,999,999 | 1.60^*^ | 0.37 | .000 | 0.6 - 2.6 |
|  |  | Rp 5,000,000 - Rp 9,999,999 - Rp 20,000,000 - Rp 49,999,999 | 2.47^*^ | 0.61 | .001 | 0.8 - 4.14 |
|  |  | Rp 10,000,000 - Rp 19,999,999 - < Rp 3,000,000 | -1.95^*^ | 0.42 | .000 | -3.09 - -0.81 |
|  |  | Rp 10,000,000 - Rp 19,999,999 - Rp 3,000,000 - Rp 4,999,999 | -2.06^*^ | 0.37 | .000 | -3.07 - -1.05 |
|  |  | Rp 10,000,000 - Rp 19,999,999 - Rp 5,000,000 - Rp 9,999,999 | -1.60^*^ | 0.37 | .000 | -2.6 - -0.6 |
|  |  | Rp 10,000,000 - Rp 19,999,999 - Rp 20,000,000 - Rp 49,999,999 | 0.87 | 0.65 | .670 | -0.91 - 2.64 |
|  |  | Rp 20,000,000 - Rp 49,999,999 - < Rp 3,000,000 | -2.82^*^ | 0.64 | .000 | -4.58 - -1.06 |
|  |  | Rp 20,000,000 - Rp 49,999,999 - Rp 3,000,000 - Rp 4,999,999 | -2.93^*^ | 0.61 | .000 | -4.61 - -1.25 |
|  |  | Rp 20,000,000 - Rp 49,999,999 - Rp 5,000,000 - Rp 9,999,999 | -2.47^*^ | 0.61 | .001 | -4.14 - -0.8 |
|  |  | Rp 20,000,000 - Rp 49,999,999 - Rp 10,000,000 - Rp 19,999,999 | -0.87 | 0.65 | .670 | -2.64 - 0.91 |
| Emotional Exhaustion at Work | Age Group | <26 years - 26-30 years | 2.23 | 3.52 | .996 | -8.18 - 12.64 |
|  |  | <26 years - 31-40 years | 3.72 | 3.47 | .936 | -6.52 - 13.97 |
|  |  | <26 years - 41-50 years | 6.75 | 3.49 | .459 | -3.57 - 17.07 |
|  |  | <26 years - 51-60 years | 9.98 | 3.53 | .072 | -0.45 - 20.42 |
|  |  | <26 years - 61-70 years | 13.31^*^ | 3.77 | .008 | 2.19 - 24.44 |
|  |  | <26 years - >71 years | 12.83 | 8.08 | .690 | -11.02 - 36.69 |
|  |  | 26-30 years - <26 years | -2.23 | 3.52 | .996 | -12.64 - 8.18 |
|  |  | 26-30 years - 31-40 years | 1.49 | 0.85 | .577 | -1.01 - 4 |
|  |  | 26-30 years - 41-50 years | 4.52^*^ | 0.95 | .000 | 1.73 - 7.32 |
|  |  | 26-30 years - 51-60 years | 7.75^*^ | 1.09 | .000 | 4.55 - 10.96 |
|  |  | 26-30 years - 61-70 years | 11.08^*^ | 1.70 | .000 | 6.07 - 16.09 |
|  |  | 26-30 years - >71 years | 10.60 | 7.35 | .778 | -11.09 - 32.3 |
|  |  | 31-40 years - <26 years | -3.72 | 3.47 | .936 | -13.97 - 6.52 |
|  |  | 31-40 years - 26-30 years | -1.49 | 0.85 | .577 | -4 - 1.01 |
|  |  | 31-40 years - 41-50 years | 3.03^*^ | 0.71 | .000 | 0.92 - 5.14 |
|  |  | 31-40 years - 51-60 years | 6.26^*^ | 0.89 | .000 | 3.63 - 8.89 |
|  |  | 31-40 years - 61-70 years | 9.59^*^ | 1.58 | .000 | 4.93 - 14.25 |
|  |  | 31-40 years - >71 years | 9.11 | 7.32 | .876 | -12.5 - 30.73 |
|  |  | 41-50 years - <26 years | -6.75 | 3.49 | .459 | -17.07 - 3.57 |
|  |  | 41-50 years - 26-30 years | -4.52^*^ | 0.95 | .000 | -7.32 - -1.73 |
|  |  | 41-50 years - 31-40 years | -3.03^*^ | 0.71 | .000 | -5.14 - -0.92 |
|  |  | 41-50 years - 51-60 years | 3.23^*^ | 0.98 | .018 | 0.33 - 6.13 |
|  |  | 41-50 years - 61-70 years | 6.56^*^ | 1.63 | .001 | 1.74 - 11.38 |
|  |  | 41-50 years - >71 years | 6.08 | 7.33 | .982 | -15.57 - 27.73 |
|  |  | 51-60 years - <26 years | -9.98 | 3.53 | .072 | -20.42 - 0.45 |
|  |  | 51-60 years - 26-30 years | -7.75^*^ | 1.09 | .000 | -10.96 - -4.55 |
|  |  | 51-60 years - 31-40 years | -6.26^*^ | 0.89 | .000 | -8.89 - -3.63 |
|  |  | 51-60 years - 41-50 years | -3.23^*^ | 0.98 | .018 | -6.13 - -0.33 |
|  |  | 51-60 years - 61-70 years | 3.33 | 1.72 | .455 | -1.74 - 8.4 |
|  |  | 51-60 years - >71 years | 2.85 | 7.35 | 1.000 | -18.85 - 24.56 |
|  |  | 61-70 years - <26 years | -13.31^*^ | 3.77 | .008 | -24.44 - -2.19 |
|  |  | 61-70 years - 26-30 years | -11.08^*^ | 1.70 | .000 | -16.09 - -6.07 |
|  |  | 61-70 years - 31-40 years | -9.59^*^ | 1.58 | .000 | -14.25 - -4.93 |
|  |  | 61-70 years - 41-50 years | -6.56^*^ | 1.63 | .001 | -11.38 - -1.74 |
|  |  | 61-70 years - 51-60 years | -3.33 | 1.72 | .455 | -8.4 - 1.74 |
|  |  | 61-70 years - >71 years | -0.48 | 7.47 | 1.000 | -22.52 - 21.57 |
|  |  | >7 years - <26 years | -12.83 | 8.08 | .690 | -36.69 - 11.02 |
|  |  | >7 years - 26-30 years | -10.60 | 7.35 | .778 | -32.3 - 11.09 |
|  |  | >7 years - 31-40 years | -9.11 | 7.32 | .876 | -30.73 - 12.5 |
|  |  | >7 years - 41-50 years | -6.08 | 7.33 | .982 | -27.73 - 15.57 |
|  |  | >7 years - 51-60 years | -2.85 | 7.35 | 1.000 | -24.56 - 18.85 |
|  |  | >7 years - 61-70 years | 0.48 | 7.47 | 1.000 | -21.57 - 22.52 |
|  | Marital Status | Not married without children - Not married with children | 6.366^*^ | 1.56 | .000 | 2.35 - 10.38 |
|  |  | Not married without children - Married without children | -0.33 | 1.10 | .990 | -3.15 - 2.48 |
|  |  | Not married without children - Married with children | 3.717^*^ | 0.75 | .000 | 1.79 - 5.65 |
|  |  | Not married with children - Not married without children | -6.366^*^ | 1.56 | .000 | -10.38 - -2.35 |
|  |  | Not married with children - Married without children | -6.701^*^ | 1.66 | .000 | -10.97 - -2.43 |
|  |  | Not married with children - Married with children | -2.65 | 1.45 | .264 | -6.39 - 1.09 |
|  |  | Married without children - Not married without children | 0.33 | 1.10 | .990 | -2.48 - 3.15 |
|  |  | Married without children - Not married with children | 6.701^*^ | 1.66 | .000 | 2.43 - 10.97 |
|  |  | Married without children - Married with children | 4.051^*^ | 0.94 | .000 | 1.64 - 6.47 |
|  |  | Married with children - Not married without children | -3.717^*^ | 0.75 | .000 | -5.65 - -1.79 |
|  |  | Married with children - Not married with children | 2.65 | 1.45 | .264 | -1.09 - 6.39 |
|  |  | Married with children - Married without children | -4.051^*^ | 0.94 | .000 | -6.47 - -1.64 |
|  | Academic Rank | Teaching staff - Lecturer | -0.44 | 0.89 | .987 | -2.86 - 1.98 |
|  |  | Teaching staff - Assistant professor | 1.74 | 0.85 | .244 | -0.58 - 4.07 |
|  |  | Teaching staff - Associate professor | 3.83^*^ | 1.14 | .007 | 0.72 - 6.94 |
|  |  | Teaching staff - Professor | 7.95^*^ | 1.93 | .000 | 2.67 - 13.22 |
|  |  | Lecturer - Teaching staff | 0.44 | 0.89 | .987 | -1.98 - 2.86 |
|  |  | Lecturer - Assistant professor | 2.19^*^ | 0.68 | .012 | 0.32 - 4.05 |
|  |  | Lecturer - Associate professor | 4.28^*^ | 1.02 | .000 | 1.49 - 7.06 |
|  |  | Lecturer - Professor | 8.39^*^ | 1.86 | .000 | 3.3 - 13.48 |
|  |  | Assistant professor - Teaching staff | -1.74 | 0.85 | .244 | -4.07 - 0.58 |
|  |  | Assistant professor - Lecturer | -2.19^*^ | 0.68 | .012 | -4.05 - -0.32 |
|  |  | Assistant professor - Associate professor | 2.09 | 0.99 | .215 | -0.61 - 4.79 |
|  |  | Assistant professor - Professor | 6.20^*^ | 1.85 | .007 | 1.16 - 11.25 |
|  |  | Associate professor - Teaching staff | -3.83^*^ | 1.14 | .007 | -6.94 - -0.72 |
|  |  | Associate professor - Lecturer | -4.28^*^ | 1.02 | .000 | -7.06 - -1.49 |
|  |  | Associate professor - Assistant professor | -2.09 | 0.99 | .215 | -4.79 - 0.61 |
|  |  | Associate professor - Professor | 4.11 | 2.00 | .238 | -1.34 - 9.57 |
|  |  | Professor - Teaching staff | -7.95^*^ | 1.93 | .000 | -13.22 - -2.67 |
|  |  | Professor - Lecturer | -8.39^*^ | 1.86 | .000 | -13.48 - -3.3 |
|  |  | Professor - Assistant professor | -6.20^*^ | 1.85 | .007 | -11.25 - -1.16 |
|  |  | Professor - Associate professor | -4.11 | 2.00 | .238 | -9.57 - 1.34 |
|  | Income | < Rp 3,000,000 - Rp 3,000,000 - Rp 4,999,999 | 0.32 | 0.87 | .996 | -2.05 - 2.7 |
|  |  | < Rp 3,000,000 - Rp 5,000,000 - Rp 9,999,999 | -0.14 | 0.86 | 1.000 | -2.48 - 2.2 |
|  |  | < Rp 3,000,000 - Rp 10,000,000 - Rp 19,999,999 | 3.03^*^ | 1.01 | .024 | 0.26 - 5.79 |
|  |  | < Rp 3,000,000 - Rp 20,000,000 - Rp 49,999,999 | 4.72^*^ | 1.56 | .022 | 0.45 - 8.99 |
|  |  | Rp 3,000,000 - Rp 4,999,999 - < Rp 3,000,000 | -0.32 | 0.87 | .996 | -2.7 - 2.05 |
|  |  | Rp 3,000,000 - Rp 4,999,999 - Rp 5,000,000 - Rp 9,999,999 | -0.46 | 0.72 | .968 | -2.43 - 1.51 |
|  |  | Rp 3,000,000 - Rp 4,999,999 - Rp 10,000,000 - Rp 19,999,999 | 2.70^*^ | 0.90 | .023 | 0.25 - 5.16 |
|  |  | Rp 3,000,000 - Rp 4,999,999 - Rp 20,000,000 - Rp 49,999,999 | 4.40^*^ | 1.49 | .027 | 0.32 - 8.47 |
|  |  | Rp 5,000,000 - Rp 9,999,999 - < Rp 3,000,000 | 0.14 | 0.86 | 1.000 | -2.2 - 2.48 |
|  |  | Rp 5,000,000 - Rp 9,999,999 - Rp 3,000,000 - Rp 4,999,999 | 0.46 | 0.72 | .968 | -1.51 - 2.43 |
|  |  | Rp 5,000,000 - Rp 9,999,999 - Rp 10,000,000 - Rp 19,999,999 | 3.17^*^ | 0.89 | .003 | 0.74 - 5.59 |
|  |  | Rp 5,000,000 - Rp 9,999,999 - Rp 20,000,000 - Rp 49,999,999 | 4.86^*^ | 1.48 | .010 | 0.8 - 8.91 |
|  |  | Rp 10,000,000 - Rp 19,999,999 - < Rp 3,000,000 | -3.03^*^ | 1.01 | .024 | -5.79 - -0.26 |
|  |  | Rp 10,000,000 - Rp 19,999,999 - Rp 3,000,000 - Rp 4,999,999 | -2.70^*^ | 0.90 | .023 | -5.16 - -0.25 |
|  |  | Rp 10,000,000 - Rp 19,999,999 - Rp 5,000,000 - Rp 9,999,999 | -3.17^*^ | 0.89 | .003 | -5.59 - -0.74 |
|  |  | Rp 10,000,000 - Rp 19,999,999 - Rp 20,000,000 - Rp 49,999,999 | 1.69 | 1.58 | .822 | -2.62 - 6.01 |
|  |  | Rp 20,000,000 - Rp 49,999,999 - < Rp 3,000,000 | -4.72^*^ | 1.56 | .022 | -8.99 - -0.45 |
|  |  | Rp 20,000,000 - Rp 49,999,999 - Rp 3,000,000 - Rp 4,999,999 | -4.40^*^ | 1.49 | .027 | -8.47 - -0.32 |
|  |  | Rp 20,000,000 - Rp 49,999,999 - Rp 5,000,000 - Rp 9,999,999 | -4.86^*^ | 1.48 | .010 | -8.91 - -0.8 |
|  |  | Rp 20,000,000 - Rp 49,999,999 - Rp 10,000,000 - Rp 19,999,999 | -1.69 | 1.58 | .822 | -6.01 - 2.62 |
