# Supplementary material for: Mental health in academia: A national survey on risk and protective factors in the Indonesian context
Source: PLoS One. 2026 Sep 21;21(9):e0358652. doi: 10.1371/journal.pone.0358652 (PMC13599600; doi:10.1371/journal.pone.0358652)
Supplement: Supplementary file 5 [file pone.0358652.s004.docx]

**S4 Table. Results of Multiple Regression Analyses Predicting Mental Health.**

|  | **B** | **β** | **SE (HC3)** | **95% CI** | **t** | **p** | **B** | **β** | **SE (HC3)** | **95% CI** | **t** | **p** |
| --- | --- | --- | --- | --- | --- | --- | --- | --- | --- | --- | --- | --- |
|  | **DV: Well Being** | | | | | | **DV: Work Satisfaction** | | | | | |
| Gender | -.125 | -.004 | .632 | [-1.363, 1.114] | -.197 | .843 | .105 | .008 | .305 | [-.493, .704] | .346 | .729 |
| Age | .118 | .080 | .041 | [.038, .198] | 2.897 | .004 | .021 | .030 | .019 | [-.016, .058] | 1.135 | .257 |
| Marital and Parental Status | .603 | .018 | .739 | [-.846, 2.052] | .817 | .414 | .061 | .004 | .384 | [-.693, .815] | .159 | .874 |
| Academic Rank | -.022 | -.001 | .391 | [-.789, .746] | -.060 | .956 | .667 | .095 | .201 | [.273, 1.060] | 3.323 | **< .001** |
| Type of University | -.488 | -.016 | .681 | [-1.824, .848] | -.717 | .474 | -.869 | -.059 | .366 | [-1.590, -.151] | -2.376 | .018 |
| Geographical Location | .053 | .002 | .644 | [-1.210, 1.317] | .083 | .934 | -.342 | -.025 | .321 | [-.972, .287] | -1.067 | .286 |
| Income | .223 | .030 | .245 | [-.257, .704] | .912 | .362 | .088 | .024 | .121 | [-.149, .324] | .725 | .469 |
| Secondary (Non-Academic) Employment | -.214 | -.008 | .895 | [-1.969, 1.541] | -.240 | .811 | .469 | .034 | .448 | [-.410, 1.350] | 1.047 | .295 |
| Publication Stress | -.366 | -.146 | .072 | [-.507, -.225] | -5.100 | **< .001** | -.251 | -.207 | .038 | [-.325, -.177] | -6.637 | **< .001** |
| Workload: Weekly work hours | .069 | .011 | .142 | [-.210, .348] | .483 | .629 | -.036 | -.012 | .071 | [-.175, .103] | -.503 | .615 |
| Workload: Work on weekends and holidays | -.220 | -.021 | .285 | [-.778, .338] | -.772 | .440 | -.071 | -.014 | .151 | [-.367, .225] | -.471 | .638 |
| Workload: Work overtime and at night | .008 | .001 | .266 | [-.515, .531] | .030 | .976 | -.357 | -.071 | .139 | [-.630, -.084] | -2.568 | .010 |
| Unwanted Behavior at the Workplace | -4.990 | -.119 | .933 | [-3.158, -6.820] | -5.350 | **< .001** | -2.307 | -.114 | .514 | [-1.299, -3.315] | -4.488 | **< .001** |
| Personal Mastery | 1.130 | .394 | .087 | [.955, 1.298] | 12.912 | **< .001** | .448 | .325 | .044 | [.362, .534] | 1.209 | **< .001** |
| Relational: Number of significant others | .802 | .075 | .228 | [.355, 1.249] | 3.520 | **< .001** | .346 | .067 | .115 | [.120, .573] | 3.003 | .003 |
| Relational: Quality time with significant others | 1.422 | .063 | .583 | [.277, 2.566] | 2.440 | .015 | -.351 | -.032 | .298 | [-.935, .234] | -1.177 | .239 |
| Self-care: Time to do hobbies | 1.552 | .069 | .614 | [.347, 2.753] | 2.530 | .012 | 1.100 | .102 | .324 | [.463, 1.730] | 3.389 | **< .001** |
| Self-care: Time to exercise | 2.068 | .094 | .538 | [1.012, 3.123] | 3.844 | **< .001** | .286 | .027 | .274 | [-.252, .824] | 1.044 | .297 |

|  | **B** | **β** | **SE (HC3)** | **95% CI** | **t** | **p** | **B** | **β** | **SE (HC3)** | **95% CI** | **t** | **p** |
| --- | --- | --- | --- | --- | --- | --- | --- | --- | --- | --- | --- | --- |
|  | **DV: Happiness at Work** | | | | | | **DV: Stress** | | | | | |
| Gender | .349 | .030 | .243 | [-.128, .825] | 1.434 | .152 | .809 | .061 | .271 | [.277, 1.342] | 2.984 | .003 |
| Age | .032 | .054 | .016 | [.000, .064] | 1.974 | .049 | -.103 | -.150 | .019 | [-.141, -.065] | -5.300 | **< .001** |
| Marital and Parental Status | .251 | .019 | .286 | [-.311, .812] | .876 | .381 | .212 | .014 | .304 | [-.385, .810] | .697 | .486 |
| Academic Rank | .243 | .041 | .163 | [-.077, .563] | 1.491 | .136 | .391 | .058 | .188 | [.022, .761] | 2.076 | .038 |
| Type of University | -.074 | -.006 | .282 | [-.627, .480] | -.261 | .794 | .640 | .045 | .300 | [.051, 1.230] | 2.133 | .033 |
| Geographical Location | .349 | .030 | .256 | [-.154, .851] | 1.362 | .173 | -.139 | -.010 | .279 | [-.686, .407] | -.500 | .617 |
| Incomef | .123 | .041 | .095 | [-.064, .310] | 1.294 | .196 | -.080 | -.023 | .111 | [-.298, .138] | -.721 | .471 |
| Secondary (Non-Academic) Employment | .145 | .013 | .344 | [-.530, .820] | .422 | .673 | .118 | .009 | .402 | [-.670, .906] | .294 | .769 |
| Publication Stress | -.190 | -.188 | .032 | [-.253, -.127] | -5.900 | **< .001** | .161 | .138 | .033 | [.096, .227] | 4.844 | **< .001** |
| Workload: Weekly work hours | -.048 | -.019 | .057 | [-.160, .065] | -.833 | .405 | .050 | .018 | .066 | [-.079, .179] | .759 | .448 |
| Workload: Work on weekends and holidays | -.041 | -.010 | .115 | [-.266, .185] | -.353 | .724 | .019 | .004 | .128 | [-.232, .270] | .146 | .884 |
| Workload: Work overtime and at night | -.178 | -.043 | .112 | [-.398, .041] | -1.594 | .111 | .103 | .021 | .121 | [-.135, .342] | .851 | .395 |
| Unwanted Behavior at the Workplace | -2.673 | -.158 | .443 | [-1.804, -3.543] | -6.033 | **< .001** | 1.938 | .100 | .377 | [2.680, 1.200] | 5.142 | **< .001** |
| Personal Mastery | .429 | .372 | .034 | [.362, .495] | 12.682 | **< .001** | -.669 | -.504 | .037 | [-.742, -.596] | -18.011 | **< .001** |
| Relational: Number of significant others | .242 | .056 | .089 | [.067, .418] | 2.715 | .007 | -.097 | -.020 | .108 | [-.308, .114] | -.902 | .367 |
| Relational: Quality time with significant others | .206 | .023 | .240 | [-.266, .677] | .856 | .392 | -.349 | -.034 | .239 | [-.818, .120] | -1.46 | .144 |
| Self-care: Time to do hobbies | .881 | .098 | .255 | [.380, 1.383] | 3.451 | **< .001** | -.777 | -.075 | .262 | [-1.290, -.264] | -2.970 | .003 |
| Self-care: Time to exercise | -.004 | .001 | .218 | [-.432, .423] | -.020 | .984 | -.150 | -.015 | .229 | [-.599, .300] | -.654 | .514 |

|  | **B** | **β** | **SE (HC3)** | **95% CI** | **t** | **p** | **B** | **β** | **SE (HC3)** | **95% CI** | **t** | **p** |
| --- | --- | --- | --- | --- | --- | --- | --- | --- | --- | --- | --- | --- |
|  | **DV: Anxiety** | | | | | | **DV: Depression** | | | | | |
| Gender | .525 | .061 | .188 | [.156, .894] | 2.790 | .005 | .359 | .039 | .196 | [-.026, .743] | 1.831 | .067 |
| Age | -.053 | -.117 | .012 | [-.077, -.029] | -4.392 | **< .001** | -.053 | -.104 | .012 | [-.073, -.027] | -4.210 | **< .001** |
| Marital and Parental Status | -.051 | -.005 | .228 | [-.499, .398] | -.222 | .825 | -.185 | -.017 | .239 | [-.654, .285] | -.772 | .440 |
| Academic Rank | .223 | .050 | .134 | [-.040, .485] | 1.665 | .096 | -.034 | -.007 | .116 | [-.261, .193] | -.293 | .770 |
| Type of University | .492 | .053 | .210 | [.079, .905] | 2.339 | .020 | .472 | .047 | .214 | [.053, .891] | 2.210 | .027 |
| Geographical Location | -.272 | -.031 | .201 | [-.667, .123] | -1.351 | .177 | .133 | .014 | .202 | [-.264, .530] | .657 | .511 |
| Income | -.074 | -.032 | .071 | [-.214, .066] | -1.038 | .300 | -.087 | -.036 | .074 | [-.231, .057] | -1.181 | .238 |
| Secondary (Non-Academic) Employment | -.157 | -.018 | .267 | [-.681, .366] | -.590 | .555 | -.182 | -.020 | .280 | [-.732, .367] | -.651 | .515 |
| Publication Stress | .108 | .141 | .024 | [.060, .156] | 4.416 | **< .001** | .088 | .107 | .023 | [.043, .133] | 3.824 | **< .001** |
| Workload: Weekly work hours | .068 | .037 | .048 | [-.025, .161] | 1.434 | .152 | .035 | .018 | .047 | [-.057, .126] | .741 | .459 |
| Workload: Work on weekends and holidays | .071 | .022 | .094 | [-.114, .256] | .754 | .451 | .254 | .075 | .099 | [.061, .447] | 2.580 | .010 |
| Workload: Work overtime and at night | .182 | .058 | .084 | [.017, .348] | 2.170 | .030 | .156 | .046 | .090 | [-.021, .333] | 1.7225 | .085 |
| Unwanted Behavior at the Workplace | 1.86 | .145 | .357 | [2.560, 1.160] | 5.201 | **< .001** | 1.789 | .131 | .335 | [2.440 ,1.130] | 5.344 | **< .001** |
| Personal Mastery | -.345 | -.397 | .030 | [-.404, -.287] | -11.647 | **< .001** | -.379 | -.408 | .026 | [-.429, -.329] | -14.770 | **< .001** |
| Relational: Number of significant others | -.019 | -.006 | .073 | [-.163, .124] | -.264 | .792 | -.057 | -.016 | .077 | [-.207, .093] | -.746 | .456 |
| Relational: Quality time with significant others | -.084 | -.012 | .194 | [-.465, .296] | -.435 | .664 | -.382 | -.053 | .178 | [-.732, -.033] | -2.148 | .032 |
| Self-care: Time to do hobbies | -.454 | -.067 | .190 | [-.827, -.080] | -2.383 | .017 | -.527 | -.073 | .189 | [-.897, -.157] | -2.792 | .005 |
| Self-care: Time to exercise | -.221 | -.033 | .167 | [-.549, .107] | -1.320 | .187 | -.688 | -.096 | .167 | [-1.020, -.360] | -4.117 | **< .001** |

|  | **B** | **β** | **SE (HC3)** | **95% CI** | **t** | **p** |
| --- | --- | --- | --- | --- | --- | --- |
|  | **DV: Emotional Exhaustion** | | | | | |
| Gender | .492 | .022 | .443 | [-.378, 1.360] | 1.110 | .267 |
| Age | -.180 | -.157 | .028 | [-.235, -.126] | -6.510 | **< .001** |
| Marital and Parental Status | -.067 | -.003 | .559 | [-1.160, 1.030] | -.119 | .905 |
| Academic Rank | .302 | .027 | .281 | [-.249, .853] | 1.076 | .282 |
| Type of University | .859 | .036 | .496 | [-.114, 1.830] | 1.732 | .084 |
| Geographical Location | -1.63 | -.074 | .466 | [-2.550, -.719] | -3.505 | **< .001** |
| Incomef | .075 | .013 | .168 | [-.254, .404] | .449 | .653 |
| Secondary (Non-Academic) Employment | .084 | .004 | .632 | [-1.160, 1.320] | .134 | .894 |
| Publication Stress | .390 | .201 | .055 | [.282, .498] | 7.081 | **< .001** |
| Workload: Weekly work hours | .580 | .124 | .109 | [.366, .795] | 5.321 | **< .001** |
| Workload: Work on weekends and holidays | .763 | .094 | .213 | [.346, 1.180] | 3.587 | **< .001** |
| Workload: Work overtime and at night | .988 | .123 | .199 | [.598, 1.380] | 4.972 | **< .001** |
| Unwanted Behavior at the Workplace | 3.876 | .119 | .785 | [5.420, 2.340] | 4.939 | **< .001** |
| Personal Mastery | -.683 | -.309 | .064 | [-.809, -.557] | -10.613 | **< .001** |
| Relational: Number of significant others | -.250 | -.030 | .179 | [-.600, .101] | -1.400 | .162 |
| Relational: Quality time with significant others | .492 | .028 | .414 | [-.321, 1.310] | 1.188 | .235 |
| Self-care: Time to do hobbies | -1.870 | -.108 | .437 | [-2.730, -1.010] | -4.278 | **< .001** |
| Self-care: Time to exercise | -.319 | -.019 | .380 | [-1.060, .426] | -.841 | .401 |
