# Supplementary material for: Mental health in academia: A national survey on risk and protective factors in the Indonesian context
Source: PLoS One. 2026 Sep 21;21(9):e0358652. doi: 10.1371/journal.pone.0358652 (PMC13599600; doi:10.1371/journal.pone.0358652)
Supplement: Supplementary file 6 [file pone.0358652.s005.docx]

*“*A national survey on risk and protective factors in the Indonesian context” (Manuscript Number: PONE-D-25-66508)

I read with interest the paper titled “A national survey on risk and protective factors in the Indonesian context”: The Authors addressed a scant literature topic and tried to contribute with the goal of exploring the risk and protective factors linked to mental health challenges of academic faculty in Indonesia: The cross-sectional survey relies on a large sample and on a set of assessing tools, thus possibly contributing to the enhancement and completion of the research topic

I have several comments to be addressed before the study could actually contribute according to its aims:

1. **Introduction**

Introduction is fluent and well written reporting an updated showcase of scientific literature available up to now and the related still open research questions. It is a worthy to be mentioned the presentation of national cultural values and related challenges in the work-family life balance of academics, alongside with the ministry regulation and ethical challenges met.

The introduction could be improved by better highlighting the reason because mental health of academics needs attention and what would be the key-points for this, even by stressing the link between academic performance and academics’ mental health. It would be useful also to have a more in-depth description of the variables of the previous studies on the same topic cited (i.e. gender, age, status as possible risk and/or risk factors)

2. **Materials and methos could be replaced by Current study**

It is expected to have the number and ratio of participants who accepted the invitation to collaborate in the survey already here and a mention on missing data treatment. It is not clear how many participants accepted and how many were invited.

The section on measures for risk and protective factors need to report internal consistency for all measures and sub-measures reported.

In general, this section could be better reorganized redundancies and following a standard format organization

3. **Description of the measures**

The Measures section would benefit from a more consistent description of the instruments. For some scales, the authors provide example items, whereas for others they do not. Given the large number of measures included in the study, it would be helpful to provide at least one example item for each multi-item scale or study-specific indicator, so that readers can better understand what each construct captures.

4. **Summary table of instruments**

Given the number and heterogeneity of the measures used, I would encourage the authors to include a summary table of the instruments. This table could report, for each construct, the instrument used, number of items, response scale, possible score range, direction of scoring, reliability in the present sample, and whether the measure was previously validated, translated/adapted, modified for the work context, or developed for this study. This would also help readers contextualize the descriptive means reported in the Results.

5. **Psychometric adequacy of adapted or modified measures**

The manuscript reports internal consistency coefficients for several multi-item scales, which is useful. However, several measures appear to have been translated, adapted to the work context, or used as selected subscales/items. For these measures, Cronbach’s alpha alone may not be sufficient to establish that the adapted items retain the expected structure of the original instrument. When multi-item scales or subscales are used as single composite scores, the authors should provide evidence that the items load onto the expected factor structure, for example a one-factor structure for adapted unidimensional scales or subscales. This seems particularly relevant for the work-adapted SWLS, the work-adapted SHS, the MBI Emotional Exhaustion subscale, the PPQ-r publication stress subscale, and the Pearlin Mastery Scale.

6. **Results**

In general, this section appears organized in a too analytical format and could benefit from a more synthetic and targeted way of presentation

7. **Missing data and effective sample size**

The manuscript reports an analytic sample of 1,380 participants who completed all seven mental health outcome measures. However, the degrees of freedom reported for the regression models, for example F(18, 1229), suggest that the effective sample size in these analyses was smaller than the full analytic sample. The authors should clarify the number of participants included in each regression model and explain how missing data on predictors were handled. If listwise deletion was used, this should be explicitly stated.

Relatedly, it would be useful to know whether participants excluded from the regression analyses because of missing predictor data differed from those retained in the models. If feasible, the authors should compare included and excluded cases on key demographic or occupational characteristics, such as gender, age, academic rank, income, type of university, and geographical location. This would help readers evaluate whether missing data may have introduced systematic bias.

8. **Reporting of t-tests and ANOVAs**

The authors state that group differences were examined using independent-sample t-tests and one-way ANOVAs. However, the Results section mainly reports means, standard deviations, and bolded values in the table. The corresponding inferential statistics should be reported more fully, including test statistics, degrees of freedom, exact p-values, and effect sizes. For ANOVAs, post-hoc comparisons should also be reported, preferably with correction for multiple comparisons.

9. **Specification of regression strategy**

The Data Analysis section states that multiple regression analyses were conducted and that each model included 18 predictors, but it does not specify the model-building strategy. It should be clarified whether predictors were entered simultaneously, hierarchically in theoretically defined blocks, or through a stepwise/forward/backward procedure. If a simultaneous model was used, this should be explicitly stated. If a hierarchical or blockwise approach was used, the order of blocks and the incremental variance explained by each block should be reported.

10. **Coding of categorical predictors**

The coding of categorical predictors in the regression models should be clarified. Variables such as marital/parental status and type of university are nominal or composite categorical variables, and it is unclear whether they were entered as dummy-coded predictors, contrast-coded predictors, or as single numerically coded variables. The note to Table 5 provides numerical coding for these variables, but it does not fully clarify how they were entered into the regression models. If dummy or contrast coding was used, the reference categories should be reported. If single numerical coding was used, the rationale and implications for interpretation should be explained.

11. **Multicollinearity diagnostics**

Given the large number of predictors included in the same regression models, the authors should report multicollinearity diagnostics, such as VIF and tolerance values. This is particularly important because several predictors may be conceptually and statistically overlapping, including age, academic rank, income, workload indicators, secondary employment, publication stress, personal mastery, and workplace safety. If multicollinearity is detected, the authors may consider a more parsimonious or theoretically structured modelling strategy, such as hierarchical/blockwise models or domain-specific sensitivity analyses.

12. **Full reporting of regression results**

Table 5 is central to the manuscript, but in its current form it largely duplicates the information already summarized in the text, reporting selected standardized coefficients and significance levels. The table does report model-level statistics, including R², adjusted R², F values, and overall model p-values; however, these are placed in the last row and could be presented more clearly, for example in a model-summary section.

More importantly, the table does not provide the full statistical information needed to evaluate the individual predictors. Because each model reportedly included 18 predictors, the authors should report the full regression output for all predictors, including non-significant ones. At minimum, this should include unstandardized coefficients, standard errors, standardized beta coefficients, t values, exact p-values, and 95% confidence intervals. Reporting indices of the unique contribution of individual predictors, such as partial or semi-partial correlations or semi-partial R², would also improve interpretability. If this information is too extensive for the main text, the full regression tables could be provided in the Supplementary Materials.

**The current state of mental health among academics in Indonesia** could be reworded in a more synthetic and targeted way

It is not clear to me how authors could state that levels of mental health issues were mild or moderate

13. **Discussion**

Discussion needs to be integrated by conclusion or final consideration and could include a summary of key points highlighted with related best practice suggestions

14. **Use of causal or directional language**

Possible bidirectional explanations could be briefly acknowledged; for example, emotional exhaustion may be associated with higher perceived publication stress, but exhaustion may also shape how publication demands and workplace safety are perceived.

To sum up my comment I think that the study could convey a valuable contribution; However, in its current state needs to be substantially improved for reaching the Journal standard for publication
