# Supplementary material for: Mental health in academia: A national survey on risk and protective factors in the Indonesian context
Source: PLoS One. 2026 Sep 21;21(9):e0358652. doi: 10.1371/journal.pone.0358652 (PMC13599600; doi:10.1371/journal.pone.0358652)
Supplement: Supplementary file 7 [file pone.0358652.s008.docx]

# Submission

# Mental health in academia: A national survey on risk and protective factors in the Indonesian context

Dear reviewers,

First, we would like to thank you for your constructive input and for recognizing our effort to improve this paper. Regarding your feedback, below we have written down your comments, which we put in italics. Your comments are followed by our response and the associated changes made in the text (the line information refers to the "Revised Manuscript_Clean" file). We hope you will appreciate the new version, and we look forward to finalizing this draft and preparing it for publication.

# Reviewer #1

1. *The introduction emphasizes work–family conflict and work engagement as key determinants of academic well-being; however, these constructs are not operationalized in the methods.*

**Our response:**

Thank you for pointing this out. Since work–family conflict and work engagement were not measured in this study, we removed these constructs from the Introduction.

**Changes made in the text:**

The following sentences were removed from the Introduction (page 6):

Furthermore, these pressures can also conflict with academics’ work engagement and disrupt the balance between work and other aspects of life. In this context, work–family balance and work engagement play a crucial role in promoting happiness among academics [12], whereas work–family conflict can undermine it [13], highlighting how stressors and supports within the productivity pressures substantially shape academic happiness.

2. *The operationalization of psychological safety appears inconsistent with established definitions. The current measurement measures exposure to workplace violence rather than psychological safety.*

**Our response:**

Thank you for this important observation. We agree that the measure reflects exposure to unwanted behavior at the workplace rather than psychological safety. We replaced the term “psychological safety” with “experience of unwanted behavior at the workplace” throughout the manuscript. Accordingly, we revised the terminology throughout the manuscript and repositioned the variable as a risk factor in the Abstract, Introduction, and Methods section. Corresponding changes were also made in the Results and Discussion sections.

3. *Satisfaction With Life Scale (SWLS) adapted to work context: was reliability and validity tested after adaptation? Was the coping measured with validated instruments?*

**Our response:**

Thank you for this important comment. Reliability coefficients for all study measures were already reported in the manuscript. In the revised version, we have included evidence of construct validity for adapted measurement scales (SWLS in work context, SHS in work context, MBI, PPQ, and PMS).

Regarding the adaptation of the Satisfaction With Life Scale (SWLS) to the work context, reliability and construct validity analyses were conducted using the present study data, and the results have now been added to the manuscript.

Coping was measured using the Pearlin Mastery Scale, a validated instrument assessing individuals' perceived personal coping and sense of control over life circumstances. The scale was adapted into Indonesian through a translation and expert review process to support content validity. In the revised manuscript, we have added a description of this adaptation process and included evidence of construct validity from the current study.

The study also assessed relational coping and self-care coping. However, these constructs were measured using two self-report items rather than standardized psychometric instruments.

**Changes made in text:**

Now we write:

Construct validity for SWLS: (Page 14 lines 245-246)

In the present study, CFA supported the expected one-factor structure (CFI = .99, TLI = .97, GFI = .98, RMSEA = .11, SRMR = .02).

Construct validity for SHS: (Pages 14-15 lines 255-257)

In the present study, CFA supported the expected one-factor structure (CFI = .99, TLI = .99, GFI = .99, RMSEA = .04, SRMR = .01).

Construct validity for MBI: (Page 16 lines 291-292)

In the present study, CFA supported the expected one-factor structure (CFI = .98, TLI = .97, GFI = .98, RMSEA = .08, SRMR = .03).

Construct validity for PPQ: (Page 17 lines 304-305)

In the present study, CFA supported the expected one-factor structure (CFI = .98, TLI = .96, GFI = .98, RMSEA = .08, SRMR = .03).

Construct validity for PMS: (Page 18 lines 329-330)

In the present study, CFA supported the expected one-factor structure (CFI = .98, TLI = .96, GFI = .98, RMSEA = .06, SRMR = .03).

SWLS: (Pages 14 lines 239-242)

Work satisfaction was measured using the Satisfaction With Work Scale (SWWS) [51], a work-context version of the Satisfaction With Life Scale (SWLS) [52–54]. It is a 5-item scale measuring cognitive evaluations of one’s work life satisfaction (e.g., “In most ways my life is close to my ideal”).

PMS: (Pages 17-18 lines 323-327)

Perceived control over life circumstances was measured using the Pearlin Mastery Scale [70], a 7-item scale measure of perceived personal coping and mastery over life circumstances (e.g., “I have little control over the things that happen to me”). The scale was translated and adapted into Indonesian through a forward–backward translation procedure, followed by expert review and cognitive interviews.

4. *Statistical tests results are missing in Table 2. In table 5: Many predictors shown but no coefficient for many variables, for example- marital status, rank, university type, income.*

**Our response:**

We acknowledge that the original version of Table 2 did not include the complete statistical test results. In response to this comment, we have provided the full statistical test results, including the relevant test statistics and exact p-values, in the Supplementary Material. To maintain the readability of the main manuscript and avoid an overly dense table, Table 2 has been retained as a concise summary of the key findings.

In the previous version of the manuscript, we reported only the statistically significant coefficients in Table 5. Following the reviewer's suggestion, we have revised Table 5 to include the coefficients for all predictor variables.

**Changes made in text:**

See Table 2 (Pages 22-23 lines 428-432), Table 5 (Pages 31-33 lines 545-573), and S3 Table.

5. *The manuscript states: Older academics reported lower distress (β = .15). Seems to be inappropriate interpretation as positive beta indicates increase, not decrease.*

**Our response:**

Thank you for this careful observation. The negative sign (-) in the beta coefficient was inadvertently omitted in the manuscript text and should have been consistent with the value reported in Table 5. We have corrected the text to accurately reflect the negative association between age and psychological distress.

**Changes made in text:**

Now we write: (Pages 30 lines 532-534)

Older academics reported lower psychological distress, including reduced stress (β = -.15, p < .001), anxiety (β = -.12, p < .001), depression (β = -.10, p < .001), and emotional exhaustion (β = -.16, p < .001).

6. *Please add attrition analysis.*

**Our response:**

Thank you for this suggestion. We have conducted an attrition analysis comparing participants who completed both waves with those who did not complete the follow-up assessment. The results have been added to the manuscript, with the full details provided in the Supplementary Materials.

**Changes made in the text:** (Page 18-19 lines 346-352)

An attrition analysis was conducted by comparing completers and non-completers across demographic characteristics using Pearson's chi-square tests. Significant differences were observed for institution type, χ²(3) = 9.04, p = .029, and geographic location, χ²(1) = 5.06, p = .025. No significant differences were found for gender, age group, marital and parental status, academic rank, income, or secondary (non-academic) employment category (all ps > .05). Detailed attrition analysis results are provided in S2 Table.

# Reviewer #2

A. *Abstract*
1. *The abstract contains an internal contradiction that must be resolved before publication. The problem statement asserts that mental health challenges in this population have risen sharply, driven by systemic pressures. Yet the results section of the abstract reports that severe psychiatric symptoms were uncommon, with high stress affecting only 2.0%, severe anxiety 3.2%, and severe or very severe depression 3.9% of participants. These two framings are in tension and may mislead readers. The authors should either: (a) revise the problem statement to acknowledge that elevated subclinical distress is prevalent even when severe symptoms are rare, and that mild-to-moderate distress still carries significant individual and institutional costs; or (b) revise the framing in line with the actual findings, noting that while systemic pressures are real and well-documented internationally, Indonesian academics in this sample reported broadly mild-to-moderate distress with a small minority experiencing severe symptoms.*

**Our response:**

Thank you for this insightful comment and for providing helpful alternatives. We adopted option (a) and revised the abstract to provide a more nuanced framing of mental health challenges among academics. Specifically, we now acknowledge that systemic pressures have only partially contributed to these challenges and emphasize that the levels of psychological distress observed in our sample were generally mild to moderate.

**Changes made in text:**

(Page 3 lines 28-30)

However, mental-health challenges in this population have risen in recent years, partially driven by systemic pressures such as hypercompetition, heavy workloads, job insecurity, and escalating expectations for research productivity and publication output.

(Page 3 lines 35-37)

Most participants reported low/minimal anxiety (63.1%) and moderate stress (63.8%), while depression was mostly low/minimal (43.3%) or mild (36.3%).

2. *Additionally, the abstract should include at minimum one sentence contextualising the key regression findings quantitatively (e.g., the effect sizes for personal mastery and publication stress), so that readers can appreciate the magnitude of associations, not just their direction.*

**Our response:**

Thank you for this valuable suggestion. We have revised the abstract to include quantitative information on the key regression findings by reporting the effect sizes (*β* coefficients).

**Changes made in text:**

Now we added: (Page 3 lines 39-45)

Across the risk and protective factors examined, higher publication stress and greater experience of unwanted behavior at the workplace were consistently associated with lower levels of well-being and higher psychological distress (β = -.19 to .20 and -.15 to .16, respectively; all ps < .001). In contrast, higher personal mastery was consistently associated with better mental-health (β = .39), greater work satisfaction (β = .33), higher happiness at work (β = .37), and lower stress (β = -.50), anxiety (β = -.40), depression (β = -.41), and emotional exhaustion (β = -.31) (all ps < .001).

B. *Introduction*

*I. Conceptual framing*

*The introduction frames publication pressure and performance-based evaluation systems as inherently damaging to academic mental health. This position is too one-sided and risks alienating readers who work within or design such systems. The authors should acknowledge that the relationship between performance expectations and well-being is conditional: when institutions provide adequate support, mentorship, and resources, high expectations need not be detrimental. The discussion of Indonesia-specific pressures (lines 63–73) is valuable and should be retained, but qualified accordingly.*

**Our response:**

Thank you for this suggestion. We revised the Introduction to provide a more balanced discussion of performance expectations and academic well-being.

**Changes made in text:**

We revised the relevant paragraph as follows:

Previous version:

"It is stated that higher education institutions’ quality is benchmarked through international ranking systems, which eventually leads to academic productivity being measured in a major part through their research and publications. Such pressures are consistently associated with adverse outcomes, including emotional exhaustion, reduced job satisfaction, and increased turnover intentions [2,11]."

Revised version: (Pages 5-6 lines 74-80)

" It is stated that higher education institutions’ quality is benchmarked through international ranking systems, which has lead academic productivity to be evaluated largely through research outputs and publications. While such expectations may foster academic achievement and institutional development, they may also contribute to emotional exhaustion, reduced job satisfaction [2,11], and increased turnover intentions [12], particularly when accompanied by limited institutional support, mentoring, or resources."

II. *Specific line comments*

1. *Lines 118–119: The sentence regarding plagiarism cases involving university rectors is out of place in a section discussing systemic pressures on academic mental health. If the authors wish to illustrate the consequences of publication pressure on research integrity, a more direct link to the mental health implications (e.g., the psychological burden of being pressured into unethical conduct) should be made explicit, or the example should be removed.*

**Our response:**

Thank you for this observation. We agree that this example was not directly relevant to the focus of the present study and have therefore removed it.

**Changes made in the text:** (Page 8 lines 136)

The sentence, "A notable example includes plagiarism cases involving university rectors who were declared not guilty despite substantial evidence," was removed from the manuscript.

2. *Lines 131–132: The phrase “empirical studies focusing on the mental health of academics in Indonesia are remain nonexistent” is grammatically incorrect. Please revise throughout. Given that the claim of total nonexistence is strong, the authors should confirm through a current literature search that no relevant empirical work has been published.*

**Our response:**

Thank you for pointing this out. We revised the sentence to correct the grammatical error and to avoid an overly strong claim. Following an updated literature review, we found that empirical studies on the mental health of Indonesian academics remain limited. The text has been revised accordingly.

**Changes made in the text:** (Page 9 line 154-155)

“In contrast to the extensive body of international research, empirical studies focusing on the mental health of academics in Indonesia are remain limited.”

3. *Lines 134–135: “Below are 4 questions we would like to address” should be written in the past tense and revised to academic register. Suggested revision: “This study addressed four research questions:”*

**Our response:**

Thank you for this suggestion. We revised the sentence to follow an appropriate academic style and consistent use of tense.

**Changes made in the text:** (Page 9 line 157)

“This study addressed four research questions:”

4. *Lines 139–142: Research questions 3 and 4 are framed using the causal language “associate with” and “buffer against”. These imply directionality that cannot be established with a cross-sectional design. Revise to explicitly correlational language: e.g., “To what extent are risk factors such as workload and publication pressure associated with mental health outcomes?”*

**Our response:**

Thank you for this comment. We agree that the original wording could imply causal interpretations that are not supported by a cross-sectional design. We have revised the language throughout the manuscript, replacing causal or directional terms with correlational wording

**Changes made in text:**

Now we write: (Page 9 lines 161-164)

To what extent are risk factors such as workload, publication pressure, and experiences of unwanted behavior at the workplace associated with mental health outcomes?

To what extent are protective factors such as coping strategies associated with mental health in this population?

*C. Materials and Methods*

*Design*

*This subsection correctly identifies the cross-sectional survey design.*

1. *However, it should not include information on ethics approval and pre-registration, which belong in dedicated subsections or in an Ethical Considerations paragraph at the end of the Methods section. The design subsection should focus on the rationale for the design choice and its appropriateness for the research questions.*

**Our response:**

Thank you for this suggestion. We removed information on ethics approval and pre-registration from the Design subsection and relocated it to a separate Ethical Considerations paragraph at the end of the Methods section. The Design subsection now focuses on the study design and its appropriateness for addressing the research questions. (see Pages 10 lines 169, Page 20 lines 389-391)

*Participants and Sampling*

1. *A section on sampling strategy and sample size rationale is entirely absent. This is a significant omission. The authors should address the following:*
i. *What was the target population, and how was it defined? (E.g., all academics holding a NIDK registration number in Indonesia’s 4,400+ higher education institutions.)*

**Our response:**

Thank you for this comment. We have clarified the target population in the Methods section.

**Changes made in text:**

Now we write: (Page 10 lines 171-173)

We targeted all academics holding a NIDK registration number in Indonesia’s 4,416 higher education institutions (https://pddikti.kemdiktisaintek.go.id/statistik). Based on https://pddikti.kemdiktisaintek.go.id/statistik, there were 303,067 academics.

ii. *Was a minimum sample size calculated a priori? If so, based on what parameters (effect size, alpha, power)?*

**Our response:**

Although no formal a priori power analysis was conducted, our preregistration specified a target sample size of 1,500 participants, following a comparable study conducted in the Netherlands (https://osf.io/5vn3q). In response to the reviewer's suggestion, we additionally calculated the minimum sample size using Cochran's formula for large populations (Cochran, 1977; Ahmed, 2024), assuming a 95% confidence level, a 3% margin of error, and maximum variability (p = .50), which indicated a minimum required sample of 1,067 participants. Our final sample of 1,380 participants exceeded this threshold.

**Change made in text:** (Page 10 lines 173-176)

We calculate minimum sample size requirements using Cochran's Formula for large populations [47,48], assuming a 95% confidence level, a 3% margin of error, and maximum variability (p = .50). The calculation indicated a minimum sample size of 1.067.

iii. *How does the achieved sample of 1,380 completers out of 2,675 who accessed the link relate to the estimated population?*

*The effective response/completion rate should be reported and discussed as a potential*

*source of bias.*

**Our response:**

Thank you for this helpful suggestion. We have now reported the survey completion rate in the Methods section. To assess the potential for attrition bias, we also conducted an attrition analysis comparing completers and non-completers on demographic characteristics. Significant differences were found for institution type and geographic location, whereas no significant differences were observed for gender, age group, marital status, parental status, academic rank, income, or secondary employment. These findings have been added to the manuscript and are acknowledged as a potential source of selection bias when interpreting the results.

**Changes made in manuscript:**

In the methods section, we write: (pages 18-19 lines 344-352)

Of the 2,675 academics who accessed the survey link, 2,477 met the inclusion criteria, 2,307 provided informed consent and voluntarily participated, and 1,380 completed all seven mental health outcome measures, corresponding to a completion rate of 51.6%. An attrition analysis was conducted by comparing completers and non-completers across demographic characteristics using Pearson's chi-square tests. Significant differences were observed for institution type, χ²(3) = 9.04, p = .029, and geographic location, χ²(1) = 5.06, p = .025. No significant differences were found for gender, age group, marital and parental status, academic rank, income, or secondary (non-academic) employment category (all ps > .05). Detailed attrition analysis results are provided in S2 Table. (Page 21-22 lines 411-419)

In the limitation section, we write: (Page 40 lines 741-745)

The completion rate was 51.6% of those who accessed the survey link (59.8% of consenting participants). Attrition analyses indicated that completers and non-completers were largely comparable across most demographic characteristics. Yet differences by institution type and geographic location suggest that some degree of selection bias cannot be ruled out.

iv. *The current Participants subsection focuses on recruitment channels rather than describing who participants are. The demographic description should open this section, and recruitment details should appear under Procedure.*

**Our response:**

Thank you for these valuable comments. Participant characteristics are now presented at the Participants subsection, while recruitment procedures have been moved to the Data Collection section. (see Pages 12-13, lines 196-217)

IV. *Procedure / Data Collection*

1. *Rename this subsection “Data Collection” to be more informative. Also, clarify the following:*
i. *Was the survey available only in Bahasa Indonesia, or also in other languages? This is relevant for validity in multilingual regions outside Java.*
ii. *The data collection window (27 July to 31 October 2025) should be noted, along with any follow-up reminders sent.*
iii. *The use of paid social media promotion (“Instagram/dosenupdate”) is an unusual recruitment channel for an academic survey. The authors should briefly justify this choice and discuss its potential effect on sample representativeness.*

**Our response:**

Thank you for these helpful suggestions. We renamed the subsection “Procedure” to “Data Collection” and added details regarding the survey language, data collection period, and follow-up reminders.

We clarified that the survey was administered only in Bahasa Indonesia, given that academics in Indonesian higher education institutions are expected to be proficient in the national language. We also specified that data collection took place from 27 July to 31 October 2025. To encourage participation, periodic reminders were disseminated through the same professional networks and social media channels used for recruitment; however, no individual follow-up was conducted because the survey was anonymous.

We further explained the use of paid social media promotion through Instagram/dosen update as a strategy to achieve broader national coverage beyond the authors’ institutional networks. The potential implications for sample representativeness are acknowledged in the Limitations section.

**Changes made in text:**

In the methods section, no we write: (Pages 12-13 lines 197-217)

Data were collected using an online self-administered questionnaire distributed through multiple recruitment channels. Initially, the survey was disseminated via institutional networks, including the Indonesian Psychology Higher Education Organizer Association (AP2TPI), professional associations (e.g., the Indonesian Campus Workers' Union), and various WhatsApp groups for academics (e.g., lecturer groups across multiple universities). Personalized email invitations were also sent to 5,827 lecturers.

After one month of recruitment, an evaluation showed that the initial strategy had not yielded the targeted sample size. Formal invitations were therefore distributed through Regional Higher Education Service Institutions (LLDIKTI; formerly known as KOPERTIS). As the targeted sample size had still not been reached after another month, the survey was subsequently promoted through Dosen Update (https://www.instagram.com/dosenupdate/), an Instagram account followed by approximately 96,200 Indonesian academics and sharing information relevant to higher education and academic life.

Data collection was conducted between 27 July and 31 October 2025. The survey was administered in Bahasa Indonesia, the national language and the primary language of instruction in Indonesian higher education. Given that all participants were university academics, no language-related barriers to survey completion were anticipated. Participation was voluntary, and electronic written informed consent was obtained online prior to survey completion. Anonymity and confidentiality were assured. To respect participants’ autonomy and preserve anonymity, individuals who did not complete the survey were not contacted for follow-up or reminder purposes.

In the limitation section, now we write: (Page 40 lines 731-734)

Paid social media promotion helped achieve broad national coverage, however, recruitment relied on voluntary participation and social media exposure. As a result, the sample may not be fully representative of all Indonesian academics, as early-career academics, who are generally more active on social media, may have been more likely to participate.

*V. Measurements*

1. *Lines 182–183: The authors state that the Satisfaction With Life Scale (SWLS) was “adapted to the work context”. This is a significant modification of a widely-used instrument. The authors must specify exactly which items were changed and provide a rationale. Adaptation without psychometric re-validation in the new context is a methodological concern that should be acknowledged as a limitation.*

**Our response:**

Thank you for this important comment. We agree that the adaptation of a widely used instrument should be described more explicitly. In the revised manuscript, we have clarified that work satisfaction was measured using the Satisfaction With Work Scale (SWWS) [51], an established work-context version of the Satisfaction With Life Scale (SWLS). We then translated and adapted the scale for use in the Indonesian context. In addition, we assessed the psychometric properties of the Indonesian version of the SWWS in the present sample. The results showed acceptable construct validity and internal consistency. These findings have also been reported in the revised manuscript.

**Changes made in text:** (Page 14 lines 239-247)

Work satisfaction was measured using the Satisfaction With Work Scale (SWWS) [51], a work-context version of the Satisfaction With Life Scale (SWLS) [52–54]. It is a 5-item scale measuring cognitive evaluations of one’s work life satisfaction (e.g., “In most ways my life is close to my ideal”). Responses were rated on a 7-point Likert scale ranging from 1 (strongly disagree) to 7 (strongly agree). The scale was translated and adapted into Indonesian through a forward–backward translation procedure, followed by expert review and cognitive interviews to ensure conceptual and cultural equivalence. In the present study, CFA supported the expected one-factor structure (CFI = .99, TLI = .97, GFI = .98, RMSEA = .11, SRMR = .02). The scale also demonstrated good internal consistency (α = .90).

2. *Lines 189–193: The same concern applies to the Subjective Happiness Scale, which was also modified for the work context. Were new items generated? How was equivalence established with the original scale?*

**Our response:**

In the revised manuscript, we have also clarified how the SHS was adapted for the work context and examined the psychometric properties of the adapted scale, including its construct validity and internal consistency.

**Changes made in text:** (Page 14 lines 252-253)

Items were adapted to the work context while preserving the original item structure and response format. An example item is “In general, I consider myself very happy at work.”

3. *Lines 203–204: “The Indonesian version” of the GAD-7 is mentioned without citation at the point of first use. Ensure all instrument versions are cited on first mention.*

**Our response:**

Thank you for pointing this out. We have now included the appropriate citation for the Indonesian version of the GAD-7 in the revised manuscript.

**Changes made in text:** (Page 15 lines 267-268)

Anxiety severity was assessed using the Indonesian version of the Generalized Anxiety Disorder Scale (GAD-7) [63,64]

4. *Lines 251–256: The operationalisation of ‘psychological safety’ using a workplace violence/unwanted behaviour measure is unconventional and potentially confounded. Psychological safety in the organisational literature (Edmondson, 1999) refers to a shared belief that interpersonal risk-taking is safe, not simply the absence of violence. The authors should either (a) cite and justify this operationalisation explicitly, or (b) relabel this variable as ‘workplace safety’ or ‘freedom from workplace violence’ throughout the manuscript to avoid conceptual confusion.*

**Our response:**

Thank you for this important observation. We agree that the measure does not capture psychological safety as conceptualized by Edmondson (1999). To avoid conceptual confusion, we adopted option (b) and relabeled the variable as “experience of unwanted behavior at the workplace” throughout the manuscript. Corresponding revisions were made in the Introduction, research questions, Methods, Results, and Discussion sections.

*D. Data Analysis*

*The statistical approach is generally sound, but several clarifications are needed:*

1. *The authors report using multiple regression but do not specify whether OLS regression, logistic regression, or another variant was used. Given that all outcome variables are continuous, OLS is implied, but this should be stated explicitly.*
2. *The use of Bonferroni correction is appropriate, but the authors should note that with 18 predictors across 7 outcome models, this approach is conservative. The authors should acknowledge that some associations may be true effects that failed to reach the corrected threshold.*
3. *Assumptions of multiple regression (normality of residuals, absence of multicollinearity, homoscedasticity) are not mentioned. Were these checked? This is especially important given the inclusion of 18 predictors.*
4. *The paper does not report confidence intervals for any regression coefficients. These need to be added to Table 5.*

**Our response:**

Thank you for these valuable suggestions. We have revised the manuscript accordingly. Specifically, we clarified in the Statistical Analysis section that Ordinary Least Squares (OLS) multiple linear regression was used. We also added a description of the assumption checks conducted prior to the regression analyses, including assessments of the normality of residuals, multicollinearity, and homoscedasticity. In addition, we re-ran all regression analyses using heteroscedasticity-consistent (HC3) robust standard errors to obtain more reliable parameter estimates in the presence of heteroscedasticity. Regarding the Bonferroni correction, we acknowledged its conservative nature in the Limitations section. Finally, we have provided the complete regression results in the S4 Table. We also have revised Table 5 to report standardized regression coefficients (β) for all predictors, which improves clarity while keeping the table concise.

**Changes made in text:**

Now we write:

1. Multiple regression analyses using the Ordinary Least Squares (OLS) estimation method … (Page 19 lines 355-356)
2. Finally, the use of the Bonferroni correction to account for multiple comparisons. Although this approach reduces the risk of Type I error, it is conservative and may increase the likelihood of Type II error. Consequently, non-significant results may still be true effects and should be taken into account in future research endeavours. (Page 40 lines 745-748)
3. Regression assumptions, including normality of residuals, multicollinearity, and homoscedasticity, were evaluated prior to the multiple regression analyses. Visual inspection of the histograms and normal Q–Q plots indicated that the residuals were mostly normally distributed, with only slight departures from normality. Multicollinearity was examined using tolerance and variance inflation factor (VIF) statistics, with tolerance values ranging from .411 to .960 and VIF values ranging from 1.041 to 2.432, indicating no problematic multicollinearity. Homoscedasticity was assessed using scatterplots of standardized residuals and the Breusch–Pagan test. As some evidence of heteroscedasticity was observed, all regression models were estimated using heteroscedasticity-consistent (HC3) robust standard errors. (Page 19 lines 360-368)
4. See Table 5 (Pages 31-33 lines 545-573) and S4 Table

*E. Results*

1. *Lines 285–286: The sentence noting that Indonesia has more than 4,400 higher education institutions employing around 303,000 academics is contextual background and belongs in the Introduction, not the Results section.*

**Our response:**

Thank you for this suggestion. We agree that this information is not appropriate for the Results section. Rather than moving it to the Introduction, we have relocated it to the Methods section, where it provides context for the target population and the sampling strategy. (see Method section Page 10 lines 171-173)

2. *Lines 299–302: Income figures are reported only in Indonesian Rupiah (IDR). Given that this paper is submitted to an international journal, US dollar equivalents (or purchasing power parity equivalents) should be provided alongside IDR figures, at least in a note, to allow international readers to contextualise the income ranges.*

**Our response:**

Thank you for this helpful suggestion. We added approximate US dollar equivalents alongside the income categories to facilitate interpretation by international readers.

**Changes made in text:**

See Table 1 (Pages 11-12 lines 194-195), Table 2 (Pages 23-24 lines 428-432), and Table 5 (Pages 31-33 lines 545-573)

Now we write:

< IDR 3,000,000 (< USD 167)

IDR 3,000,000 – IDR 4,999,999 (USD 167 – 278)

IDR 5,000,000 – IDR 9,999,999 (USD 278 – 556)

IDR 10,000,000 – IDR 19,999,999 (USD 556 – 1,111)

IDR 20,000,000 – IDR 49,999,999 (USD 1,111 – 2,778)

3. *Table 2 is informative but would benefit from the addition of test statistics (t or F values) and exact p-values alongside the bolded indicators of significance. Readers should be able to evaluate the magnitude of group differences, not just their significance.*

**Our response:**

Thank you for this comment. We agree that reporting the full statistical results would improve the transparency of the findings and facilitate the interpretation of the results. Therefore, we have provided the complete statistical results in S2 Table, while keeping Table 2 concise.

**Changes made in text:**

See S3 Table

4. *The discussion of workload patterns (lines 349–356) is descriptive. However, the finding that 40.5% of academics work more than 40 hours per week, in excess of the Indonesian legal maximum, is a substantively important finding that deserves clearer prominence. Consider presenting this figure in the abstract or highlighting it in the discussion.*

**Our response:**

Thank you for this valuable comment and for offering a sharper perspective on the importance of this finding. We have therefore highlighted this result more prominently in the Abstract (see Page 3 lines 33-35)

5. *Table 3: (Types of unwanted workplace behaviour) - the authors should clarify how the categorisation was developed. Was it derived inductively from open-ended responses, or was it based on a pre-specified taxonomy? This has implications for the validity of the categorisation.*

**Our response:**

Thank you for this important question. We clarified the measurement and coding procedures in the Data Analysis sections. The categorization was primarily based on predefined response options, supplemented by an open-ended “other” category. Responses provided under “other” were subsequently reviewed and coded by the research team.

**Changes made in text:**

Data Analysis: (Pages 20 lines 382-387)

For experiences of unwanted behavior in the workplace, responses provided under the “other” option were further examined to determine whether they could be incorporated into the predefined categories. These responses were reviewed and coded by the research team. Where applicable, responses were recategorized into the existing categories, while responses that could not be classified within them were grouped into new categories based on similarities in their content.

Results: (Page 26 lines 468-471)

Additional forms emerged from responses provided under the “other” category, including professional injustice, character defamation and slander, psychological harassment or emotional abuse, and political or organizational violence.

6. *The regression results in Table 5 present standardised beta coefficients but are missing confidence intervals and standard errors. Please add these for completeness and to meet PLOS One reporting standards.*

**Our response:**

Thank you for this suggestion. The complete regression results have been provided in S4 Table. We have revised Table 5 to report standardized regression coefficients (β) for all predictors, which improves clarity while keeping the table concise.

**Changes made in text:**

See Table 5 (Pages 31-33 lines 545-573) and S4 Table

*F. Discussion*

II. *Weaknesses*
1. *The discussion repeatedly uses causal language (“contributes to”, “protect against”, “buffer”) that is not supported by the cross-sectional design. All such language must be revised to acknowledge the correlational nature of the findings. For example, “mastery predicts lower distress” should read “mastery was associated with lower distress”.*

**Our response:**

Thank you for this comment. We agree and have revised the manuscript to avoid causal language throughout, replacing it with wording that reflects the correlational nature of the findings (e.g., “was associated with” instead of “predicts” or “protects against”).

2. *The interpretation of workload in lines 496–501 is speculative. The authors suggest that academics may not experience workload-related stress because of intrinsic motivation (pengabdian) and cognitive engagement. This is a plausible hypothesis, but it is not tested in the data. The authors should acknowledge this explicitly and frame this as an area for future qualitative research rather than a finding.*

**Our response:**

Thank you. We have revised this section to frame this as a possible explanation and a topic for future qualitative research, rather than as a finding from the present study.

**Changes made in text:** (Pages 34 lines 596-601)

One possible explanation, which was not examined in the present study, is that academic work may be driven by intrinsic motivation and a strong sense of calling and commitment to service, commonly referred to as pengabdian in the Indonesian context, which may be associated with lower levels of psychological distress [75]. Future qualitative research could explore whether these factors shape how academics experience workload and psychological distress.

3. *The cultural interpretation of the ‘without children’ vulnerability finding (lines 534–537) is interesting but requires more careful handling. The claim that parenthood is “deeply intertwined with spiritual and cultural identity” as an explanatory mechanism is not tested. The alternative explanation that unmarried and childless academics are typically younger, earlier in their careers, and therefore facing higher structural pressures should also be considered and may actually provide a more parsimonious account.*

**Our response:**

Thank you for this thoughtful comment. We agree that our previous cultural interpretation was speculative and was not directly examined in this study. We have therefore revised the Discussion by giving greater emphasis to the possibility that academics without children may be younger and at earlier career stages, where structural and career-related pressures are more common. We now present the cultural interpretation as a possible contextual factor rather than a primary explanation and acknowledge that it should be explored in future research.

**Changes made in text:** (Pages 35-36 lines 634-649)

Our study also found that academics without children were more vulnerable to mental health problems, regardless of their marital status. One possible explanation is that this association may partly reflect career stage rather than parenthood status per se. Previous research suggests that many academics postpone parenthood until their careers become more established, indicating that childlessness during this period often reflects career timing rather than a permanent life choice [81]. Consequently, some academics without children may be at earlier career stages, during which structural and career-related pressures, including establishing research productivity, securing stable employment, obtaining permanent positions, and meeting promotion expectations, are particularly pronounced and may adversely affect mental health [82]. Consistent with this interpretation, a recent systematic review identified childlessness as one of the characteristics associated with poorer mental health among university academics, although the underlying mechanisms remain unclear [45]. In the Indonesian context, cultural values surrounding parenthood may also influence how this life stage is experienced, as children are widely perceived as blessings and sources of joy and hope [83], and parenthood is often regarded as an important life transition [84]. However, these cultural mechanisms were not examined in the present study and should be explored in future research.

4. *The discussion of workplace violence (lines 586–607) is rich and thought-provoking. However, the final claim that “bullying is a mechanism through which authority is enforced and dissent is silenced” is a strong interpretive claim that goes beyond what the survey data can support. It would benefit from citation or from being framed explicitly as an interpretation rather than a finding.*

**Our response:**

Thank you for this thoughtful comment. We agree that our original wording overstated what could be concluded from our survey data. We have revised the Discussion to present this statement as a possible interpretation rather than a direct finding and clarified that it was not directly examined in the present study.

**Changes made in text:** (Page 38 lines 692-694)

One possible interpretation is that bullying may also function as a means of maintaining authority and discouraging dissent, although this was not directly examined in the present study.

III. *Limitations*

*The limitations section (lines 615–622) is appropriately brief but should be expanded to include:*

1. *The modification of validated scales (SWLS, SHS) for the work context without full psychometric re-validation, and the implications this has for the validity of the findings.*
2. *The unconventional operationalisation of psychological safety as absence of workplace violence, and how this differs from established measures of psychological safety climate.*
3. *The absence of a priori power analysis and the implications for underpowered subgroup comparisons (e.g., professors n = 35, age >71 n = 2).*
4. *The potential non-independence of observations, given that some institutions may have contributed many participants while others contributed none.*

**Our response:**

Thank you for these valuable suggestions. We have carefully addressed each of these points.

(1) First, we would like to clarify that the modified versions of the SWLS and SHS were evaluated for validity in the present sample and showed satisfactory results. We have now clarified this information in the Measures section
(2) Following the reviewer’s comment, we have removed the term “psychological safety” throughout the manuscript and replaced it with “experience of unwanted behavior at the workplace,” which more accurately reflects the measure used and avoids confusion with established measures of psychological safety climate.
(3) Although an a priori power analysis was not conducted, the target sample size was specified in the preregistration and the final sample exceeded the minimum sample size estimated using Cochran’s formula. However, we acknowledge that some subgroup comparisons included relatively small numbers of participants and should therefore be interpreted with caution.
(4) We have added the potential non-independence of observations as a limitation, as the number of participants recruited from each institution may have varied and institutional clustering was not accounted for in the analyses. These points have now been expanded in the Limitations section.

**Changes made in text:**

In methods section we add :

Psychometric properties of SWLS:

In the present study, CFA supported the expected one-factor structure (CFI = .99, TLI = .97, GFI = .98, RMSEA = .11, SRMR = .02). The scale also demonstrated good internal consistency (α = .90). (Page 15 lines 276-280)

Psychometric properties of SHS:

In the present study, CFA supported the expected one-factor structure (CFI = .99, TLI = .99, GFI = .99, RMSEA = .04, SRMR = .01). The scale also demonstrated good internal consistency (α = .83). (Page 16 lines 291-293)

In the limitation section, we add: (Page 40 lines 735-739)

Furthermore, the number of participants recruited from each institution may have varied, and institutional clustering was not accounted for in the analyses. As academics within the same institution may share similar organizational contexts, policies, and working environments, potential non-independence of observations cannot be ruled out and may have affected the precision of the estimated associations.

*Minor Comments*

1. *The figure captions for Figures 2–5 are placed in the body of the manuscript (e.g., “Fig 2. Distribution of workload indicators…”) without accompanying figure panels embedded in the manuscript. While this is standard in some submission systems, the authors should confirm that figure files are correctly uploaded and linked.*

**Our response:**

Thank you for pointing this out. We reviewed the submission files and ensured that all figures are correctly uploaded, labeled, and linked to their corresponding captions in accordance with the journal requirements.

**Changes made in text:**

See Figures 2-5

2. *The number of participants across tables is slightly inconsistent (e.g., Female n = 849 in Figures 2–5, but n = 865 in Table 1). The authors should reconcile these discrepancies and explain any differences due to missing values.*

**Our response:**

Thank you for pointing this out. The sample size differs between Table 1 and Figures 2–5 because not all participants completed all sections of the questionnaire beyond the main study variables, resulting in variable-specific missing data. In addition, the sample sizes reported in Figures 2–5 were inadvertently presented as identical, whereas the actual number of participants differs slightly across figures depending on data availability for each variable. We have corrected the sample sizes in each figure to reflect the appropriate number of valid cases.

**Changes made in text:**

See Figures 2-5

3. *Table 5 contains arrows (→) to indicate direction of regression coefficients rather than explicit positive/negative signs in the cells. This is unconventional and may not render correctly across platforms. Consider replacing these with explicit “+” and “–” annotations or simply noting the sign in the coefficient column.*

**Our response:**

Thank you for this suggestion. We confirmed that Table 5 already used explicit positive and negative signs rather than arrows to indicate the direction of the coefficients. Nevertheless, to improve clarity and accessibility, we removed the color coding from the table and now report all regression coefficients, including non-significant ones, in response to comments from another reviewer. We have also carefully checked the formatting to ensure consistent rendering across platforms.

**Changes made in text:**

See Table 5 (Pages 31-33 lines 545-573)

4. *The pre-registration link (https://osf.io/zq6ef) should be confirmed as publicly accessible.*

**Our response:**

Thank you for this comment. We have verified that the pre-registration link (https://osf.io/zq6ef) is publicly accessible.

5. *Reference 33 appears to be about gender differences in social environmental factors of psychological distress among Indonesian adolescents, which does not match the claim about plagiarism cases involving university rectors. Please check that citations are correctly matched to their claims throughout.*

**Our response:**

Thank you for pointing this out. We confirmed that Reference 33 was incorrectly cited. The appropriate reference should have been:

Putra IE, Jazilah NI, Adishesa MS, Al Uyun D, Wiratraman HP. Denying the accusation of plagiarism: power relations at play in dictating plagiarism as academic misconduct. High Educ (Dordr). 2023;85: 979–997. doi:10.1007/s10734-022-00875-z.

However, following another reviewer's suggestion that this example was not directly relevant to the study, we removed the entire passage from the manuscript. Consequently, this reference has also been deleted.

# Reviewer #3: “A national survey on risk and protective factors in the Indonesian context” (Manuscript Number: PONE-D-25-66508)

1. *Introduction*

*Introduction is fluent and well written reporting an updated showcase of scientific literature available up to now and the related still open research questions. It is a worthy to be mentioned the presentation of national cultural values and related challenges in the work-family life balance of academics, alongside with the ministry regulation and ethical challenges met.*

*The introduction could be improved by better highlighting the reason because mental health of academics needs attention and what would be the key-points for this, even by stressing the link between academic performance and academics’ mental health. It would be useful also to have a more in-depth description of the variables of the previous studies on the same topic cited (i.e. gender, age, status as possible risk and/or risk factors)*

**Our response:**

Thank you for this valuable suggestion. We have revised the Introduction to further emphasize the importance of academics’ mental health, including its implications for academic functioning, teaching, research activities, and student support. We have also expanded the discussion of previously identified risk factors by providing a more detailed description of the roles of gender, age, academic rank, and marital status in relation to academics’ mental health.

**Changes made in introduction:**

(Page 9 lines 147-153)

These findings underscore the importance of addressing mental health among academics. Beyond its implications for academics’ own well-being and functioning, poor mental health may also affect their ability to fulfill the multiple roles expected of them within higher education [45]. Academics are responsible not only for teaching, research, and institutional duties, but may also serve as an important source of support for students experiencing psychological difficulties [46]. Mental health difficulties among academics may therefore have broader implications for the academic environment.

(Pages 6-7 lines 81-100)

Demographic and occupational characteristics have consistently been identified as important determinants of academics' mental health. Gender differences are among the most consistently reported findings, with female academics generally experiencing higher levels of stress, anxiety, and burnout than their male counterparts [13,14]. These disparities have been attributed to greater work–family conflict [15], disproportionate caregiving responsibilities [16], and higher psychosocial occupational demands in academic settings [17]. As a result, female academics are more vulnerable to emotional exhaustion and burnout [18]. Gender has also been identified as a significant determinant of anxiety and depression among university teachers [19].

Age has also been associated with mental health outcomes. Younger academics generally report greater psychological distress than older colleagues [19]. Age has likewise been associated with psychosocial work hazards among university lecturers [20]. This increased vulnerability has been linked to early-career challenges, including precarious employment, career uncertainty, and intense publication and promotion pressures [21]. These occupational demands have also been associated with lower job satisfaction among academics [22]. Academic rank further contributes to mental health differences, with academics in junior positions reporting higher levels of anxiety and depression than those in senior positions [23].

Other sociodemographic characteristics have also been linked to academics' mental health. Marital status may influence psychological well-being through differences in social support and family responsibilities [24]. Balancing family commitments with academic responsibilities may further increase work-related stress and burnout [2].

2. *Materials and methods could be replaced by Current study*

*It is expected to have the number and ratio of participants who accepted the invitation to collaborate in the survey already here and a mention on missing data treatment. It is not clear how many participants accepted and how many were invited.*

*The section on measures for risk and protective factors need to report internal consistency for all measures and sub-measures reported.*

*In general, this section could be better reorganized redundancies and following a standard format organization.*

**Our response:**

We appreciate the reviewer's comment. We have carefully reviewed and revised the Measures and Method section accordingly.

First, we have retained the heading "Materials and Methods," as this conforms to the standard formatting guidelines specified by PLOS ONE.

We also clarified the participant flow, including the numbers who accessed the survey, met the eligibility criteria, provided consent, and completed the survey. As recruitment was conducted through multiple channels, the exact number of individuals who received the invitation could not be determined.

Information on missing data treatment and internal consistency for all measures and subscales has also been added.

**Changes made in text:**

Now we write

In Data Collection section: (Page 12 lines 197-209)

Data were collected using an online self-administered questionnaire distributed through multiple recruitment channels. Initially, the survey was disseminated via institutional networks, including the Indonesian Psychology Higher Education Organizer Association (AP2TPI), professional associations (e.g., the Indonesian Campus Workers' Union), and various WhatsApp groups for academics (e.g., lecturer groups across multiple universities). Personalized email invitations were also sent to 5,827 lecturers.

After one month of recruitment, an evaluation showed that the initial strategy had not yielded the targeted sample size. Formal invitations were therefore distributed through Regional Higher Education Service Institutions (LLDIKTI; formerly known as KOPERTIS). As the targeted sample size had still not been reached after another month, the survey was subsequently promoted through Dosen Update (https://www.instagram.com/dosenupdate/), an Instagram account followed by approximately 96,200 Indonesian academics and sharing information relevant to higher education and academic life.

In Data Analysis section: (Page 18-19 lines 344-352)

Of the 2,675 academics who accessed the survey link, 2,477 met the inclusion criteria, 2,307 provided informed consent and voluntarily participated, and 1,380 completed all seven mental health outcome measures, corresponding to a completion rate of 51.6%. An attrition analysis was conducted by comparing completers and non-completers across demographic characteristics using Pearson's chi-square tests. Significant differences were observed for institution type, χ²(3) = 9.04, p = .029, and geographic location, χ²(1) = 5.06, p = .025. No significant differences were found for gender, age group, marital and parental status, academic rank, income, or secondary (non-academic) employment category (all ps > .05). Detailed attrition analysis results are provided in S2 Table.

3. *Description of the measures*

*The Measures section would benefit from a more consistent description of the instruments. For some scales, the authors provide example items, whereas for others they do not. Given the large number of measures included in the study, it would be helpful to provide at least one example item for each multi-item scale or study-specific indicator, so that readers can better understand what each construct captures.*

**Our response:**

Thank you for this helpful suggestion. We agree that providing a more consistent description of the measures would improve the clarity of the manuscript. In the revised manuscript, we have added an example item for each multi-item scale.

**Changes made in text:**

See Measurements section (Pages 13-18 lines 219-341)

4. *Summary table of instruments*

*Given the number and heterogeneity of the measures used, I would encourage the authors to include a summary table of the instruments. This table could report, for each construct, the instrument used, number of items, response scale, possible score range, direction of scoring, reliability in the present sample, and whether the measure was previously validated, translated/adapted, modified for the work context, or developed for this study. This would also help readers contextualize the descriptive means reported in the Results.*

**Our response:**

We thank the reviewer for this helpful suggestion. We agree that a summary table of the instruments would improve clarity and help readers better contextualize the descriptive statistics reported in the Results. We have added S1 Table, which summarizes, for each construct, the instrument used, number of items, response scale, possible score range, direction of scoring, reliability in the present sample, and validation/adaptation status. This table is referenced in the Measurements section and cross-referenced in the Results when descriptive means are first reported.

**Changes made in text:**

Now we write:

See S1 Table for a summary of all instruments (Page 18 line 341)

See S1 Table for instrument details (Page 24 line 432)

5. *Psychometric adequacy of adapted or modified measures*

*The manuscript reports internal consistency coefficients for several multi-item scales, which is useful. However, several measures appear to have been translated, adapted to the work context, or used as selected subscales/items. For these measures, Cronbach’s alpha alone may not be sufficient to establish that the adapted items retain the expected structure of the original instrument. When multi-item scales or subscales are used as single composite scores, the authors should provide evidence that the items load onto the expected factor structure, for example a one-factor structure for adapted unidimensional scales or subscales. This seems particularly relevant for the work-adapted SWLS, the work-adapted SHS, the MBI Emotional Exhaustion subscale, the PPQ-r publication stress subscale, and the Pearlin Mastery Scale.*

**Our response:**

Thank you for this valuable comment. In the revised manuscript, Confirmatory Factor Analyses (CFA) were conducted and reported for all multi-item adapted measures used in this study. The results supported the expected factor structure of each measure, with acceptable model fit and factor loadings. These findings are reported in the Measurements section alongside the internal consistency coefficients, providing evidence for the reliability and structural validity of the measures used.

**Changes made in text:**

Now we write in Measurements section:

Psychometric properties of SWLS: (Page 14 lines 245-247)

In the present study, CFA supported the expected one-factor structure (CFI = .99, TLI = .97, GFI = .98, RMSEA = .11, SRMR = .02). The scale also demonstrated good internal consistency (α = .90).

Psychometric properties of SHS: (Pages 14-15 lines 255-257)

In the present study, CFA supported the expected one-factor structure (CFI = .99, TLI = .99, GFI = .99, RMSEA = .04, SRMR = .01). The scale also demonstrated good internal consistency (α = .83).

Psychometric properties of MBI: (Page 16 lines 291-293)

In the present study, CFA supported the expected one-factor structure (CFI = .98, TLI = .97, GFI = .98, RMSEA = .08, SRMR = .03). The scale also demonstrated good internal consistency (α = .92).

Psychometric properties of PPQ: (Page 17 lines 304-306)

In the present study, CFA supported the expected one-factor structure (CFI = .98, TLI = .96, GFI = .98, RMSEA = .08, SRMR = .03). The scale also demonstrated good internal consistency (α = .84).

Psychometric properties of PMS: (Page 28 lines 329-331)

In the present study, CFA supported the expected one-factor structure (CFI = .98, TLI = .96, GFI = .98, RMSEA = .06, SRMR = .03). The scale also demonstrated acceptable internal consistency (α = .78).

6. *Missing data and effective sample size*

*The manuscript reports an analytic sample of 1,380 participants who completed all seven mental health outcome measures. However, the degrees of freedom reported for the regression models, for example F(18, 1229), suggest that the effective sample size in these analyses was smaller than the full analytic sample. The authors should clarify the number of participants included in each regression model and explain how missing data on predictors were handled. If listwise deletion was used, this should be explicitly stated.*

*Relatedly, it would be useful to know whether participants excluded from the regression analyses because of missing predictor data differed from those retained in the models. If feasible, the authors should compare included and excluded cases on key demographic or occupational characteristics, such as gender, age, academic rank, income, type of university, and geographical location. This would help readers evaluate whether missing data may have introduced systematic bias.*

**Our response:**

We thank the reviewer for identifying this important discrepancy and for the suggestion regarding missing data handling. We confirm that the effective sample size used in each regression model was smaller than the full analytic sample of 1,380 participants. Specifically, missingness varied across the 18 predictors. As we used listwise deletion, participants with missing data on any one predictor were excluded from the regression analyses. We agree it is important for readers to evaluate whether missingness may have introduced systematic bias. In the revised manuscript, we address this through an attrition analysis comparing completers and non-completers (corresponding to a completion rate of 51.6%) across demographic characteristics, using Pearson's chi-square tests. Significant differences were observed for institution type, *χ*²(3) = 9.04, *p* = .029, and geographic location, *χ*²(1) = 5.06, *p* = .025, while no significant differences were found for gender, age group, marital and parental status, academic rank, income, or secondary (non-academic) employment category (all *p*s > .05). These results suggest that attrition was unlikely to have introduced substantial systematic bias into the final sample with respect to most demographic characteristics, although the observed differences in institution type and geographic location should be considered when interpreting the generalizability of the findings.

**Changes made in text:**

Now we write: (Page 18-19 lines 344-352)

Of the 2,675 academics who accessed the survey link, 2,477 met the inclusion criteria, 2,307 provided informed consent and voluntarily participated, and 1,380 completed all seven mental health outcome measures, corresponding to a completion rate of 51.6%. An attrition analysis was conducted by comparing completers and non-completers across demographic characteristics using Pearson's chi-square tests. Significant differences were observed for institution type, χ²(3) = 9.04, p = .029, and geographic location, χ²(1) = 5.06, p = .025. No significant differences were found for gender, age group, marital and parental status, academic rank, income, or secondary (non-academic) employment category (all ps > .05). Detailed attrition analysis results are provided in S2 Table.

7. *Reporting of t-tests and ANOVAs*

*The authors state that group differences were examined using independent-sample t-tests and one-way ANOVAs. However, the Results section mainly reports means, standard deviations, and bolded values in the table. The corresponding inferential statistics should be reported more fully, including test statistics, degrees of freedom, exact p-values, and effect sizes. For ANOVAs, post-hoc comparisons should also be reported, preferably with correction for multiple comparisons.*

**Our response:**

Thank you for this suggestion. We acknowledge that the original version of Table 2 did not include the complete statistical test results. In response to this comment, we have provided the full statistical analyses, including the post hoc results, relevant test statistics, and exact *p*-values, in the Supplementary Material. To maintain the readability of the main manuscript and avoid an overly dense table, Table 2 has been retained as a concise summary of the key findings.

**Changes made in text:**

See S3 Table

8. *Specification of regression strategy*

*The Data Analysis section states that multiple regression analyses were conducted and that each model included 18 predictors, but it does not specify the model-building strategy. It should be clarified whether predictors were entered simultaneously, hierarchically in theoretically defined blocks, or through a stepwise/forward/backward procedure. If a simultaneous model was used, this should be explicitly stated. If a hierarchical or blockwise approach was used, the order of blocks and the incremental variance explained by each block should be reported.*

**Our response:**

Thank you for this observation. All predictors were entered simultaneously into each regression model, and we have now explicitly stated this in the revised manuscript.

**Changes made in text:**

Now we write in Data Analysis section: (Page 20 lines 372-373)

All predictors were entered simultaneously into each model.

9. *Coding of categorical predictors*

*The coding of categorical predictors in the regression models should be clarified. Variables such as marital/parental status and type of university are nominal or composite categorical variables, and it is unclear whether they were entered as dummy-coded predictors, contrast-coded predictors, or as single numerically coded variables. The note to Table 5 provides numerical coding for these variables, but it does not fully clarify how they were entered into the regression models. If dummy or contrast coding was used, the reference categories should be reported. If single numerical coding was used, the rationale and implications for interpretation should be explained.*

**Our response:**

Thank you for this important comment. We agree that the coding of the categorical predictors required further clarification. In the original manuscript, marital/parental status and type of university were entered into the regression models using single numerical coding. In response to this comment, we revised the coding of these nominal variables by collapsing them into dichotomous categories and entering them as binary predictors (e.g., married vs. not married; private vs. public university). Other demographic variables with naturally ordered categories (e.g., academic rank and income level) were entered using their ordinal coding. Accordingly, we have revised the Statistical Analysis section and the footnote to Table 5 to clearly describe the coding scheme for each predictor.

**Changes made in text:**

Now we write in Data Analysis section: (Page 19 lines 373-376)

Nominal demographic variables with more than two categories were recoded into dichotomous categories and entered them as binary predictors (e.g., married vs. not married; private vs. public university). Other demographic variables with naturally ordered categories (e.g., academic rank and income level) were entered using their ordinal coding.

See Table 5 (Pages 31-33 lines 545-573)

10. *Multicollinearity diagnostics*

*Given the large number of predictors included in the same regression models, the authors should report multicollinearity diagnostics, such as VIF and tolerance values. This is particularly important because several predictors may be conceptually and statistically overlapping, including age, academic rank, income, workload indicators, secondary employment, publication stress, personal mastery, and workplace safety. If multicollinearity is detected, the authors may consider a more parsimonious or theoretically structured modelling strategy, such as hierarchical/blockwise models or domain-specific sensitivity analyses.*

**Our response:**

Thank you for this valuable suggestion. We assessed multicollinearity among all predictors by examining tolerance and Variance Inflation Factor (VIF) values. The results indicated no evidence of problematic multicollinearity, with tolerance values ranging from .411 to .960 and VIF values ranging from 1.041 to 2.432, which are well within commonly accepted thresholds. Therefore, all predictors were retained in the regression models.

**Changes made in text:**

Now we write: (Page 19 lines 363-365)

Multicollinearity was examined using tolerance and variance inflation factor (VIF) statistics, with tolerance values ranging from .411 to .960 and VIF values ranging from 1.041 to 2.432, indicating no problematic multicollinearity.

11. *Full reporting of regression results*

*Table 5 is central to the manuscript, but in its current form it largely duplicates the information already summarized in the text, reporting selected standardized coefficients and significance levels. The table does report model-level statistics, including R², adjusted R², F values, and overall model p-values; however, these are placed in the last row and could be presented more clearly, for example in a model-summary section.*

*More importantly, the table does not provide the full statistical information needed to evaluate the individual predictors. Because each model reportedly included 18 predictors, the authors should report the full regression output for all predictors, including non-significant ones. At minimum, this should include unstandardized coefficients, standard errors, standardized beta coefficients, t values, exact p-values, and 95% confidence intervals. Reporting indices of the unique contribution of individual predictors, such as partial or semi-partial correlations or semi-partial R², would also improve interpretability. If this information is too extensive for the main text, the full regression tables could be provided in the Supplementary Materials.*

*The current state of mental health among academics in Indonesia could be reworded in a more synthetic and targeted way. It is not clear to me how authors could state that levels of mental health issues were mild or moderate.*

**Our response:**

We thank the reviewer for these detailed comments. We have revised Table 5 by moving the model-summary statistics (*R*², adjusted *R*², *F* values, and overall model *p*-values) to the top of the table for each outcome, allowing readers to evaluate overall model fit before examining individual predictor effects.

We agree that reporting only selected standardized coefficients was insufficient given that each model included 18 predictors. We have now included the full regression output for all predictors in each of the seven models in the Supplementary Materials (S4 Table), including unstandardized coefficients (B), standard errors (SE), standardized beta coefficients (β), t values, exact p-values, and 95% confidence intervals. The main-text Table 5 has been retained in condensed form, with a note directing readers to the supplementary materials for the complete results.

We appreciate the reviewer's observation and would like to clarify that the classification of mental health levels (e.g., mild, moderate) followed established severity categories specified in the original validation references for each measure, as cited in the Measures section.

**Changes made in text:**

See S4 Table and Table 5 (Pages 31-33 lines 545-573)

12. *Discussion*

*Discussion needs to be integrated by conclusion or final consideration and could include a summary of key points highlighted with related best practice suggestions*

**Our response:**

Thank you for this helpful suggestion. We have revised the concluding section of the Discussion to provide a clearer summary of the key findings and their implications. The revised conclusion further emphasizes the main risk and protective factors associated with academics' mental health, incorporates practical recommendations at the individual, institutional, and policy levels, and expands the directions for future research.

**Changes made in text:** (Pages 40-41 lines 748-771)

In conclusion, this national survey reveals that Indonesian academics generally reported mild to moderate levels of stress and anxiety, while severe psychiatric symptoms were relatively uncommon. Several groups appeared particularly vulnerable, including women, younger academics, unmarried academics, and those in lower academic ranks and income groups.

Among the risk factors assessed, publication related stress and unwanted behavior in the workplace emerged as a consistent correlate of reduced well-being and greater psychological distress. In contrast, personal mastery functioned as an important protective correlate, showing consistent associations with higher levels of well-being, work satisfaction, and happiness at work, as well as lower levels of stress, anxiety, depression, and emotional exhaustion. The relatively strong association observed for mastery (β = .31 - .50) further suggest that it may represent a relevant focus for future research and intervention development. These findings point to several practical priorities for supporting mental health in academia. At the individual level, initiatives that strengthen personal mastery and coping resources may be beneficial. At the institutional level, universities should consider strategies to reduce excessive publication-related pressure, prevent and respond appropriately to unwanted behavior in the workplace, and provide additional support for groups identified as more vulnerable. Reviewing workload expectations, performance evaluation systems, career pathways, and access to mental health support may also contribute to healthier academic working environments. Future research should develop and test such approaches using longitudinal, intervention, and mixed-method designs to better understand their effectiveness and the direction of the observed associations. At a broader level, universities and national stakeholders should work toward organizational cultures and policies that support open discussion of mental health and promote sustainable and supportive working conditions for academics.

14. *Use of causal or directional language*

*Possible bidirectional explanations could be briefly acknowledged; for example, emotional exhaustion may be associated with higher perceived publication stress, but exhaustion may also shape how publication demands and workplace safety are perceived.*

**Our response:**

Thank you for this comment. We have revised the Discussion to avoid causal language by replacing "increase" with "were significantly associated with." We have also acknowledged the potential bidirectional nature of the observed associations and clarified that the cross-sectional design precludes causal inferences.

**Changes made in text:**

(Pages 36 lines 651-652)

Our findings indicate that these publication demands significantly associated with symptoms of stress, anxiety, depression, and emotional exhaustion.

(Page 37 lines 664-666)

However, these associations may be bidirectional, as higher emotional exhaustion may also influence how academics perceive publication demands and other workplace experiences.
