# Supplementary material for: Mental health in academia: A national survey on risk and protective factors in the Indonesian context
Source: PLoS One. 2026 Sep 21;21(9):e0358652. doi: 10.1371/journal.pone.0358652 (PMC13599600; doi:10.1371/journal.pone.0358652)
Supplement: Supplementary file 8 [file pone.0358652.s009.docx]

# Submission

# Mental health in academia: A national survey on risk and protective factors in the Indonesian context

Dear reviewers,

First, we would like to thank you for your constructive input and for recognizing our effort to improve this paper. Regarding your feedback, below we have written down your comments, which we put in italics. Your comments are followed by our response and the associated changes made in the text (the line information refers to the "Manuscript" file). We hope you will appreciate the new version, and we look forward to finalizing this draft and preparing it for publication.

# Reviewer #3

1. *Point 2 concerning the part related to “It is expected to have the number and ratio of participants who accepted the invitation to collaborate in the survey already here and a mention on missing data treatment. It is not clear how many participants accepted and how many were invited” has been well addressed by adding essential information on participants flow. Nevertheless, from your accurate description it appears that only a minority of the whole eligible group of participants accepted to take part in the survey.*

*This needs to be fully discussed in the conclusion and limitation section, because is part of the ecological validity of your study. I understand that the issue of completers vs non completers and the attrition analysis has been fully addressed, but it needs to be clear to the reader that only a part of the academic faculty community accepted to participate, possibly with a mention of similar studies that reported this data.*

**Our response:**

Thank you for this important comment. We have now added this point to the Limitations section.

**Changes made in the text:**

In addition, only a minority of the broader eligible academic community participated in the survey, which may limit the representativeness of the sample. Similar challenges with participation rates have also been reported in large-scale surveys of academic populations [105]. (page 40, lines 745-748)

2. *Point 12 page 40-41 on discussion, lines 748-771 needs, in my view, to be reworded in a more nuanced and cautious way, given that the sample inquired on all variables is only a small part of the whole eligible academic faculty population. This point is a strength and, at the same time, a limitation of your study. You reached a large sample which is, nevertheless, only a part of the whole population.*

**Our response:**

Thank you for this important comment. We agree that the conclusion should be phrased more cautiously to reflect that, although the study included a large national sample, it represents only a proportion of the eligible academic population. We have therefore revised the wording to avoid overgeneralization.

**Changes made in the text:**

We changes this sentence:

In conclusion, this national survey reveals that Indonesian academics generally reported mild to moderate levels of stress and anxiety, while severe psychiatric symptoms were relatively uncommon. Several groups appeared particularly vulnerable, including women, younger academics, unmarried academics, and those in lower academic ranks and income groups.

To :

In conclusion, this national survey suggests that the academics who participated reported mild to moderate levels of stress and anxiety, while severe psychiatric symptoms were relatively uncommon. Within this sample, women, younger academics, unmarried academics, and those in lower academic ranks and income groups appeared to report greater psychological vulnerability. (page 41, lines 752-755)
