# Supplementary material for: Mental health in academia: A national survey on risk and protective factors in the Indonesian context
Source: PLoS One. 2026 Sep 21;21(9):e0358652. doi: 10.1371/journal.pone.0358652 (PMC13599600; doi:10.1371/journal.pone.0358652)
Supplement: Peer review history

# Peer review: Decision Letter 0

**DOI:** 10.1371/journal.pone.0358652.r001

**Authors:** Kristiawan Indriyanto

8 Jun 2026

Dear Dr. Abidin,

Thank you for submitting your manuscript to PLOS ONE. After careful consideration, we feel that it has merit but does not fully meet PLOS ONE’s publication criteria as it currently stands. Therefore, we invite you to submit a revised version of the manuscript that addresses the points raised during the review process.

Please submit your revised manuscript by Jul 23 2026 11:59PM. If you will need more time than this to complete your revisions, please reply to this message or contact the journal office at plosone@plos.org. When you're ready to submit your revision, log on to https://www.editorialmanager.com/pone/ and select the 'Submissions Needing Revision' folder to locate your manuscript file.

- A letter that responds to each point raised by the academic editor and reviewer(s). You should upload this letter as a separate file labeled 'Response to Reviewers'.
- A marked-up copy of your manuscript that highlights changes made to the original version. You should upload this as a separate file labeled 'Revised Manuscript with Track Changes'.
- An unmarked version of your revised paper without tracked changes. You should upload this as a separate file labeled 'Manuscript'.

If you would like to make changes to your financial disclosure, please include your updated statement in your cover letter. Guidelines for resubmitting your figure files are available below the reviewer comments at the end of this letter.

If applicable, we recommend that you deposit your laboratory protocols in protocols.io to enhance the reproducibility of your results. Protocols.io assigns your protocol its own identifier (DOI) so that it can be cited independently in the future. For instructions see: https://journals.plos.org/plosone/s/submission-guidelines#loc-laboratory-protocols. Additionally, PLOS ONE offers an option for publishing peer-reviewed Lab Protocol articles, which describe protocols hosted on protocols.io. Read more information on sharing protocols at https://plos.org/protocols?utm_medium=editorial-email&utm_source=authorletters&utm_campaign=protocols.

As the corresponding author, your ORCID iD is verified in the submission system and will appear in the published article. PLOS supports the use of ORCID, and we encourage all coauthors to register for an ORCID iD and use it as well. Please encourage your coauthors to verify their ORCID iD within the submission system before final acceptance, as unverified ORCID iDs will not appear in the published article. *Only*  the individual author can complete the verification step; PLOS staff *cannot*  verify ORCID iDs on behalf of authors.

We look forward to receiving your revised manuscript.

Kind regards,

Kristiawan Indriyanto

Academic Editor

PLOS One

Journal Requirements:

When submitting your revision, we need you to address these additional requirements.

1. Please ensure that your manuscript meets PLOS ONE's style requirements, including those for file naming. The PLOS ONE style templates can be found at

https://journals.plos.org/plosone/s/file?id=wjVg/PLOSOne_formatting_sample_main_body.pdf and

https://journals.plos.org/plosone/s/file?id=ba62/PLOSOne_formatting_sample_title_authors_affiliations.pdf

2. Thank you for stating the following in the Acknowledgments Section of your manuscript:

“This research was funded by the Directorate of Research and Community Service, Directorate General of Research and Development, Ministry of Higher Education, Science, and Technology, Republic of Indonesia.”

We note that you have provided funding information that is not currently declared in your Funding Statement. However, funding information should not appear in the Acknowledgments section or other areas of your manuscript. We will only publish funding information present in the Funding Statement section of the online submission form.

Please remove any funding-related text from the manuscript and let us know how you would like to update your Funding Statement. Currently, your Funding Statement reads as follows:

“This research was funded by the Directorate of Research and Community Service, Directorate General of Research and Development, Ministry of Higher Education, Science, and Technology, Republic of Indonesia, Grant Number: 093/C3/DT.05.00/PL/2025”

Please include your amended statements within your cover letter; we will change the online submission form on your behalf.

3. Thank you for stating the following financial disclosure:

“This research was funded by the Directorate of Research and Community Service, Directorate General of Research and Development, Ministry of Higher Education, Science, and Technology, Republic of Indonesia, Grant Number: 093/C3/DT.05.00/PL/2025”

Please state what role the funders took in the study.  If the funders had no role, please state: "The funders had no role in study design, data collection and analysis, decision to publish, or preparation of the manuscript."

If this statement is not correct you must amend it as needed.

Please include this amended Role of Funder statement in your cover letter; we will change the online submission form on your behalf.

4. In the online submission form, you indicated that your data will be submitted to a repository upon acceptance.  We strongly recommend all authors deposit their data before acceptance, as the process can be lengthy and hold up publication timelines. Please note that, though access restrictions are acceptable now, your entire minimal  dataset will need to be made freely accessible if your manuscript is accepted for publication. This policy applies to all data except where public deposition would breach compliance with the protocol approved by your research ethics board. If you are unable to adhere to our open data policy, please kindly revise your statement to explain your reasoning and we will seek the editor's input on an exemption.

5. If the reviewer comments include a recommendation to cite specific previously published works, please review and evaluate these publications to determine whether they are relevant and should be cited. There is no requirement to cite these works unless the editor has indicated otherwise.

[Note: HTML markup is below. Please do not edit.]

Reviewers' comments:

Reviewer's Responses to Questions

**Comments to the Author**

1. Is the manuscript technically sound, and do the data support the conclusions?

Reviewer #1: Partly

Reviewer #2: Partly

Reviewer #3: Partly

**********

2. Has the statistical analysis been performed appropriately and rigorously?

Reviewer #1: Yes

Reviewer #2: Yes

Reviewer #3: No

**********

3. Have the authors made all data underlying the findings in their manuscript fully available?

The PLOS Data policy

Reviewer #1: Yes

Reviewer #2: No

Reviewer #3: Yes

**********

4. Is the manuscript presented in an intelligible fashion and written in standard English?

Reviewer #1: Yes

Reviewer #2: No

Reviewer #3: Yes

**********

Reviewer #1: The following corrections are required:

1. The introduction emphasizes work–family conflict and work engagement as key determinants of academic well-being; however, these constructs are not operationalized in the methods.

2. The operationalization of psychological safety appears inconsistent with established definitions. The current measurement measures exposure to workplace violence rather than psychological safety.

3. Satisfaction With Life Scale (SWLS) adapted to work context: was reliability and validity tested after adaptation? Was the coping measured with validated instruments?

4. Statistical tests results are missing in Table 2. In table 5: Many predictors shown but no coefficient for many variables, for example- marital status, rank, university type, income.

5. The manuscript states: Older academics reported lower distress (β = .15). Seems to be inappropriate interpretation as positive beta indicates increase, not decrease. 6. Please add attrition analysis.

Reviewer #2: Overall comments

This manuscript presents the first large-scale, national-level survey of academic mental health in Indonesia, surveying 1,380 academics across 32 of Indonesia's 38 provinces. The study addresses a genuine gap in the literature and contributes timely, policy-relevant evidence on a population whose mental health has received scant empirical attention in the Indonesian context. The research design is appropriate for the objectives, the instruments are well-validated, and the sample is commendably broad in terms of geography, institutional type, and academic rank.

However, the manuscript requires substantial revisions before it is suitable for publication.

The primary concerns are:

i. a conceptual tension between the framing of the problem (severe and rising distress) and the relatively mild findings

ii. structural and organisational weaknesses in the Methods section

iii. unsupported causal language given the cross-sectional design

iv. pervasive grammatical and editorial issues

v. several specific interpretive claims that overreach the data.

Editorial and Language

The manuscript contains pervasive grammatical, syntactic, and stylistic errors that require systematic correction by a native English speaker or professional editing service before resubmission. The following are illustrative examples only:

• Line 36: “the most vulnerable groups that resulting in poorer mental health outcomes” → “the groups most vulnerable to poorer mental health outcomes”

• Line 132: “empirical studies… are remain nonexistent” → “empirical studies… remain nonexistent”

• Line 134: “Below are 4 questions we would like to address” → “This study addressed four research questions”

• Line 482: “resulted in poorer mental health outcomes” (causal language; revise to “associated with”)

The use of tense is inconsistent throughout. Methods and Results sections should be written in the past tense. The Introduction may use the present tense for established facts, but claims about what the study does should use the past tense.

Citations appear in the text but are not always clearly linked to the specific claims they are meant to support. In several places (e.g., lines 56–57), the citation format and connection to the claim should be clarified.

Detailed comments on the manuscript are presented below.

a. Abstract

1. The abstract contains an internal contradiction that must be resolved before publication. The problem statement asserts that mental health challenges in this population have risen sharply, driven by systemic pressures. Yet the results section of the abstract reports that severe psychiatric symptoms were uncommon, with high stress affecting only 2.0%, severe anxiety 3.2%, and severe or very severe depression 3.9% of participants. These two framings are in tension and may mislead readers.

2. The authors should either: (a) revise the problem statement to acknowledge that elevated subclinical distress is prevalent even when severe symptoms are rare, and that mild-to-moderate distress still carries significant individual and institutional costs; or (b) revise the framing in line with the actual findings, noting that while systemic pressures are real and well-documented internationally, Indonesian academics in this sample reported broadly mild-to-moderate distress with a small minority experiencing severe symptoms.

3. Additionally, the abstract should include at minimum one sentence contextualising the key regression findings quantitatively (e.g., the effect sizes for personal mastery and publication stress), so that readers can appreciate the magnitude of associations, not just their direction.

b. Introduction

i. Conceptual framing

The introduction frames publication pressure and performance-based evaluation systems as inherently damaging to academic mental health. This position is too one-sided and risks alienating readers who work within or design such systems. The authors should acknowledge that the relationship between performance expectations and well-being is conditional: when institutions provide adequate support, mentorship, and resources, high expectations need not be detrimental. The discussion of Indonesia-specific pressures (lines 63–73) is valuable and should be retained, but qualified accordingly.

ii. Specific line comments

1. Lines 118–119: The sentence regarding plagiarism cases involving university rectors is out of place in a section discussing systemic pressures on academic mental health. If the authors wish to illustrate the consequences of publication pressure on research integrity, a more direct link to the mental health implications (e.g., the psychological burden of being pressured into unethical conduct) should be made explicit, or the example should be removed.

2. Lines 131–132: The phrase “empirical studies focusing on the mental health of academics in Indonesia are remain nonexistent” is grammatically incorrect. Please revise throughout. Given that the claim of total nonexistence is strong, the authors should confirm through a current literature search that no relevant empirical work has been published.

3. Lines 134–135: “Below are 4 questions we would like to address” should be written in the past tense and revised to academic register. Suggested revision: “This study addressed four research questions:”

4. Lines 139–142: Research questions 3 and 4 are framed using the causal language “associate with” and “buffer against”. These imply directionality that cannot be established with a cross-sectional design. Revise to explicitly correlational language: e.g., “To what extent are risk factors such as workload and publication pressure associated with mental health outcomes?”

c. Materials and Methods

i. Overall comment

The Methods section is the most in need of structural revision. It mixes recruitment, sampling, ethics, and data collection across subsections in a way that reduces clarity. Specific restructuring suggestions are offered below.

ii. Design

This subsection correctly identifies the cross-sectional survey design.

1. However, it should not include information on ethics approval and pre-registration, which belong in dedicated subsections or in an Ethical Considerations paragraph at the end of the Methods section. The design subsection should focus on the rationale for the design choice and its appropriateness for the research questions.

iii. Participants and Sampling

1. A section on sampling strategy and sample size rationale is entirely absent. This is a significant omission. The authors should address the following:

i. What was the target population, and how was it defined? (E.g., all academics holding a NIDK registration number in Indonesia’s 4,400+ higher education institutions.)

ii. Was a minimum sample size calculated a priori? If so, based on what parameters (effect size, alpha, power)?

iii. How does the achieved sample of 1,380 completers out of 2,675 who accessed the link relate to the estimated population?

a. The effective response/completion rate should be reported and discussed as a potential source of bias.

iv. The current Participants subsection focuses on recruitment channels rather than describing who participants are. The demographic description should open this section, and recruitment details should appear under Procedure.

iv. Procedure / Data Collection

1. Rename this subsection “Data Collection” to be more informative. Also, clarify the following:

i. Was the survey available only in Bahasa Indonesia, or also in other languages? This is relevant for validity in multilingual regions outside Java.

ii. The data collection window (27 July to 31 October 2025) should be noted, along with any follow-up reminders sent.

iii. The use of paid social media promotion (“Instagram/dosenupdate”) is an unusual recruitment channel for an academic survey. The authors should briefly justify this choice and discuss its potential effect on sample representativeness.

v. Measurements

1. Lines 182–183: The authors state that the Satisfaction With Life Scale (SWLS) was “adapted to the work context”. This is a significant modification of a widely-used instrument. The authors must specify exactly which items were changed and provide a rationale. Adaptation without psychometric re-validation in the new context is a methodological concern that should be acknowledged as a limitation.

2. Lines 189–193: The same concern applies to the Subjective Happiness Scale, which was also modified for the work context. Were new items generated? How was equivalence established with the original scale?

3. Lines 203–204: “The Indonesian version” of the GAD-7 is mentioned without citation at the point of first use. Ensure all instrument versions are cited on first mention.

4. Lines 251–256: The operationalisation of ‘psychological safety’ using a workplace violence/unwanted behaviour measure is unconventional and potentially confounded. Psychological safety in the organisational literature (Edmondson, 1999) refers to a shared belief that interpersonal risk-taking is safe, not simply the absence of violence. The authors should either (a) cite and justify this operationalisation explicitly, or (b) relabel this variable as ‘workplace safety’ or ‘freedom from workplace violence’ throughout the manuscript to avoid conceptual confusion.

d. Data Analysis

The statistical approach is generally sound, but several clarifications are needed:

1. The authors report using multiple regression but do not specify whether OLS regression, logistic regression, or another variant was used. Given that all outcome variables are continuous, OLS is implied, but this should be stated explicitly.

2. The use of Bonferroni correction is appropriate, but the authors should note that with 18 predictors across 7 outcome models, this approach is conservative. The authors should acknowledge that some associations may be true effects that failed to reach the corrected threshold.

3. Assumptions of multiple regression (normality of residuals, absence of multicollinearity, homoscedasticity) are not mentioned.

a. Were these checked? This is especially important given the inclusion of 18 predictors.

4. The paper does not report confidence intervals for any regression coefficients. These need to be added to Table 5.

e. Results

1. Lines 285–286: The sentence noting that Indonesia has more than 4,400 higher education institutions employing around 303,000 academics is contextual background and belongs in the Introduction, not the Results section.

2. Lines 299–302: Income figures are reported only in Indonesian Rupiah (IDR). Given that this paper is submitted to an international journal, US dollar equivalents (or purchasing power parity equivalents) should be provided alongside IDR figures, at least in a note, to allow international readers to contextualise the income ranges.

3. Table 2 is informative but would benefit from the addition of test statistics (t or F values) and exact p-values alongside the bolded indicators of significance. Readers should be able to evaluate the magnitude of group differences, not just their significance.

4. The discussion of workload patterns (lines 349–356) is descriptive. However, the finding that 40.5% of academics work more than 40 hours per week, in excess of the Indonesian legal maximum, is a substantively important finding that deserves clearer prominence. Consider presenting this figure in the abstract or highlighting it in the discussion.

5. Table 3: (Types of unwanted workplace behaviour) - the authors should clarify how the categorisation was developed.

a. Was it derived inductively from open-ended responses, or was it based on a pre-specified taxonomy? This has implications for the validity of the categorisation.

6. The regression results in Table 5 present standardised beta coefficients but are missing confidence intervals and standard errors.

a. Please add these for completeness and to meet PLOS One reporting standards.

f. Discussion

i. Strengths

1. The discussion appropriately highlights the novelty of the study and draws on relevant theoretical frameworks (e.g., Pearlin and Schooler’s mastery model). The comparison of well-being scores with other Indonesian populations is a useful contextualising exercise, though the caveat regarding non-comparable samples is appropriately noted.

ii. Weaknesses

1. The discussion repeatedly uses causal language (“contributes to”, “protect against”, “buffer”) that is not supported by the cross-sectional design. All such language must be revised to acknowledge the correlational nature of the findings. For example, “mastery predicts lower distress” should read “mastery was associated with lower distress”.

2. The interpretation of workload in lines 496–501 is speculative. The authors suggest that academics may not experience workload-related stress because of intrinsic motivation (pengabdian) and cognitive engagement. This is a plausible hypothesis, but it is not tested in the data. The authors should acknowledge this explicitly and frame this as an area for future qualitative research rather than a finding.

3. The cultural interpretation of the ‘without children’ vulnerability finding (lines 534–537) is interesting but requires more careful handling. The claim that parenthood is “deeply intertwined with spiritual and cultural identity” as an explanatory mechanism is not tested. The alternative explanation that unmarried and childless academics are typically younger, earlier in their careers, and therefore facing higher structural pressures should also be considered and may actually provide a more parsimonious account.

4. The discussion of workplace violence (lines 586–607) is rich and thought-provoking. However, the final claim that “bullying is a mechanism through which authority is enforced and dissent is silenced” is a strong interpretive claim that goes beyond what the survey data can support. It would benefit from citation or from being framed explicitly as an interpretation rather than a finding.

iii. Limitations

The limitations section (lines 615–622) is appropriately brief but should be expanded to include:

1. The modification of validated scales (SWLS, SHS) for the work context without full psychometric re-validation, and the implications this has for the validity of the findings.

2. The unconventional operationalisation of psychological safety as absence of workplace violence, and how this differs from established measures of psychological safety climate.

3. The absence of a priori power analysis and the implications for underpowered subgroup comparisons (e.g., professors n = 35, age >71 n = 2).

4. The potential non-independence of observations, given that some institutions may have contributed many participants while others contributed none.

Minor Comments

1. The figure captions for Figures 2–5 are placed in the body of the manuscript (e.g., “Fig 2. Distribution of workload indicators…”) without accompanying figure panels embedded in the manuscript.

a. While this is standard in some submission systems, the authors should confirm that figure files are correctly uploaded and linked.

2. The number of participants across tables is slightly inconsistent (e.g., Female n = 849 in Figures 2–5, but n = 865 in Table 1).

a. The authors should reconcile these discrepancies and explain any differences due to missing values.

3. Table 5 contains arrows (→) to indicate direction of regression coefficients rather than explicit positive/negative signs in the cells.

a. This is unconventional and may not render correctly across platforms. Consider replacing these with explicit “+” and “–” annotations or simply noting the sign in the coefficient column.

4. The pre-registration link (https://osf.io/zq6ef) should be confirmed as publicly accessible.

5. Reference 33 appears to be about gender differences in social environmental factors of psychological distress among Indonesian adolescents, which does not match the claim about plagiarism cases involving university rectors.

a. Please check that citations are correctly matched to their claims throughout.

Major Revisions:

1. Resolve the conceptual contradiction between the severity framing in the introduction and the mild-to-moderate findings.

2. Add a sampling rationale and sample size justification.

3. Rewrite causal language as correlational throughout.

4. Clarify and justify the modification of validated instruments (SWLS, SHS) for work context.

5. Reclassify or more carefully justify the use of ‘workplace safety/violence’ as a proxy for psychological safety.

6. Add confidence intervals and test statistics to Tables 2 and 5.

7. Restructure the Methods section for clarity (separate ethics, design, participants, data collection).

8. Engage with professional editorial support to address pervasive language issues.

Minor Revisions:

1. Add US$ equivalents alongside IDR income figures.

2. Move the population context sentence from Results to Introduction.

3. Reconcile participant ‘n’ discrepancies across tables and figures.

4. Verify and correct reference 33.

5. Clarify figure embedding and panel availability.

Reviewer #3: “A national survey on risk and protective factors in the Indonesian context” (Manuscript Number: PONE-D-25-66508)

I read with interest the paper titled “A national survey on risk and protective factors in the Indonesian context”: The Authors addressed a scant literature topic and tried to contribute with the goal of exploring the risk and protective factors linked to mental health challenges of academic faculty in Indonesia: The cross-sectional survey relies on a large sample and on a set of assessing tools, thus possibly contributing to the enhancement and completion of the research topic

I have several comments to be addressed before the study could actually contribute according to its aims:

1. Introduction

Introduction is fluent and well written reporting an updated showcase of scientific literature available up to now and the related still open research questions. It is a worthy to be mentioned the presentation of national cultural values and related challenges in the work-family life balance of academics, alongside with the ministry regulation and ethical challenges met.

The introduction could be improved by better highlighting the reason because mental health of academics needs attention and what would be the key-points for this, even by stressing the link between academic performance and academics’ mental health. It would be useful also to have a more in-depth description of the variables of the previous studies on the same topic cited (i.e. gender, age, status as possible risk and/or risk factors)

2. Materials and methos could be replaced by Current study

It is expected to have the number and ratio of participants who accepted the invitation to collaborate in the survey already here and a mention on missing data treatment. It is not clear how many participants accepted and how many were invited.

The section on measures for risk and protective factors need to report internal consistency for all measures and sub-measures reported.

In general, this section could be better reorganized redundancies and following a standard format organization

3. Description of the measures

The Measures section would benefit from a more consistent description of the instruments. For some scales, the authors provide example items, whereas for others they do not. Given the large number of measures included in the study, it would be helpful to provide at least one example item for each multi-item scale or study-specific indicator, so that readers can better understand what each construct captures.

4. Summary table of instruments

Given the number and heterogeneity of the measures used, I would encourage the authors to include a summary table of the instruments. This table could report, for each construct, the instrument used, number of items, response scale, possible score range, direction of scoring, reliability in the present sample, and whether the measure was previously validated, translated/adapted, modified for the work context, or developed for this study. This would also help readers contextualize the descriptive means reported in the Results.

5. Psychometric adequacy of adapted or modified measures

The manuscript reports internal consistency coefficients for several multi-item scales, which is useful. However, several measures appear to have been translated, adapted to the work context, or used as selected subscales/items. For these measures, Cronbach’s alpha alone may not be sufficient to establish that the adapted items retain the expected structure of the original instrument. When multi-item scales or subscales are used as single composite scores, the authors should provide evidence that the items load onto the expected factor structure, for example a one-factor structure for adapted unidimensional scales or subscales. This seems particularly relevant for the work-adapted SWLS, the work-adapted SHS, the MBI Emotional Exhaustion subscale, the PPQ-r publication stress subscale, and the Pearlin Mastery Scale.

6. Results

In general, this section appears organized in a too analytical format and could benefit from a more synthetic and targeted way of presentation

7. Missing data and effective sample size

The manuscript reports an analytic sample of 1,380 participants who completed all seven mental health outcome measures. However, the degrees of freedom reported for the regression models, for example F(18, 1229), suggest that the effective sample size in these analyses was smaller than the full analytic sample. The authors should clarify the number of participants included in each regression model and explain how missing data on predictors were handled. If listwise deletion was used, this should be explicitly stated.

Relatedly, it would be useful to know whether participants excluded from the regression analyses because of missing predictor data differed from those retained in the models. If feasible, the authors should compare included and excluded cases on key demographic or occupational characteristics, such as gender, age, academic rank, income, type of university, and geographical location. This would help readers evaluate whether missing data may have introduced systematic bias.

8. Reporting of t-tests and ANOVAs

The authors state that group differences were examined using independent-sample t-tests and one-way ANOVAs. However, the Results section mainly reports means, standard deviations, and bolded values in the table. The corresponding inferential statistics should be reported more fully, including test statistics, degrees of freedom, exact p-values, and effect sizes. For ANOVAs, post-hoc comparisons should also be reported, preferably with correction for multiple comparisons.

9. Specification of regression strategy

The Data Analysis section states that multiple regression analyses were conducted and that each model included 18 predictors, but it does not specify the model-building strategy. It should be clarified whether predictors were entered simultaneously, hierarchically in theoretically defined blocks, or through a stepwise/forward/backward procedure. If a simultaneous model was used, this should be explicitly stated. If a hierarchical or blockwise approach was used, the order of blocks and the incremental variance explained by each block should be reported.

10. Coding of categorical predictors

The coding of categorical predictors in the regression models should be clarified. Variables such as marital/parental status and type of university are nominal or composite categorical variables, and it is unclear whether they were entered as dummy-coded predictors, contrast-coded predictors, or as single numerically coded variables. The note to Table 5 provides numerical coding for these variables, but it does not fully clarify how they were entered into the regression models. If dummy or contrast coding was used, the reference categories should be reported. If single numerical coding was used, the rationale and implications for interpretation should be explained.

11. Multicollinearity diagnostics

Given the large number of predictors included in the same regression models, the authors should report multicollinearity diagnostics, such as VIF and tolerance values. This is particularly important because several predictors may be conceptually and statistically overlapping, including age, academic rank, income, workload indicators, secondary employment, publication stress, personal mastery, and workplace safety. If multicollinearity is detected, the authors may consider a more parsimonious or theoretically structured modelling strategy, such as hierarchical/blockwise models or domain-specific sensitivity analyses.

12. Full reporting of regression results

Table 5 is central to the manuscript, but in its current form it largely duplicates the information already summarized in the text, reporting selected standardized coefficients and significance levels. The table does report model-level statistics, including R², adjusted R², F values, and overall model p-values; however, these are placed in the last row and could be presented more clearly, for example in a model-summary section.

More importantly, the table does not provide the full statistical information needed to evaluate the individual predictors. Because each model reportedly included 18 predictors, the authors should report the full regression output for all predictors, including non-significant ones. At minimum, this should include unstandardized coefficients, standard errors, standardized beta coefficients, t values, exact p-values, and 95% confidence intervals. Reporting indices of the unique contribution of individual predictors, such as partial or semi-partial correlations or semi-partial R², would also improve interpretability. If this information is too extensive for the main text, the full regression tables could be provided in the Supplementary Materials.

The current state of mental health among academics in Indonesia could be reworded in a more synthetic and targeted way

It is not clear to me how authors could state that levels of mental health issues were mild or moderate

13. Discussion

Discussion needs to be integrated by conclusion or final consideration and could include a summary of key points highlighted with related best practice suggestions

14. Use of causal or directional language

Possible bidirectional explanations could be briefly acknowledged; for example, emotional exhaustion may be associated with higher perceived publication stress, but exhaustion may also shape how publication demands and workplace safety are perceived.

To sum up my comment I think that the study could convey a valuable contribution; However, in its current state needs to be substantially improved for reaching the Journal standard for publication

**********

what does this mean?). If published, this will include your full peer review and any attached files.

If you choose “no”, your identity will remain anonymous but your review may still be made public.

**Do you want your identity to be public for this peer review?**  For information about this choice, including consent withdrawal, please see our Privacy Policy

Reviewer #1: No

Reviewer #2: **Yes:** Frederick Murunga Wekesah

Reviewer #3: **Yes:** Lavinia Barone

**********

[NOTE: If reviewer comments were submitted as an attachment file, they will be attached to this email and accessible via the submission site. Please log into your account, locate the manuscript record, and check for the action link "View Attachments". If this link does not appear, there are no attachment files.]

To ensure your figures meet our technical requirements, please review our figure guidelines: https://journals.plos.org/plosone/s/figures

You may also use PLOS’s free figure tool, NAAS, to help you prepare publication quality figures: https://journals.plos.org/plosone/s/figures#loc-tools-for-figure-preparation.

NAAS will assess whether your figures meet our technical requirements by comparing each figure against our figure specifications.

Attachment Submitted filename: review.docx

# Author comment: Author response to Decision Letter 1

**DOI:** 10.1371/journal.pone.0358652.r002

5 Aug 2026

Submission

Mental health in academia: A national survey on risk and protective factors in the Indonesian context

Dear reviewers,

First, we would like to thank you for your constructive input and for recognizing our effort to improve this paper. Regarding your feedback, below we have written down your comments, which we put in italics. Your comments are followed by our response and the associated changes made in the text (the line information refers to the "Revised Manuscript_Clean" file). We hope you will appreciate the new version, and we look forward to finalizing this draft and preparing it for publication.

Reviewer #1

1. The introduction emphasizes work–family conflict and work engagement as key determinants of academic well-being; however, these constructs are not operationalized in the methods.

Our response:

Thank you for pointing this out. Since work–family conflict and work engagement were not measured in this study, we removed these constructs from the Introduction.

Changes made in the text:

The following sentences were removed from the Introduction (page 6):

Furthermore, these pressures can also conflict with academics’ work engagement and disrupt the balance between work and other aspects of life. In this context, work–family balance and work engagement play a crucial role in promoting happiness among academics [12], whereas work–family conflict can undermine it [13], highlighting how stressors and supports within the productivity pressures substantially shape academic happiness.

2. The operationalization of psychological safety appears inconsistent with established definitions. The current measurement measures exposure to workplace violence rather than psychological safety.

Our response:

Thank you for this important observation. We agree that the measure reflects exposure to unwanted behavior at the workplace rather than psychological safety. We replaced the term “psychological safety” with “experience of unwanted behavior at the workplace” throughout the manuscript. Accordingly, we revised the terminology throughout the manuscript and repositioned the variable as a risk factor in the Abstract, Introduction, and Methods section. Corresponding changes were also made in the Results and Discussion sections.

3. Satisfaction With Life Scale (SWLS) adapted to work context: was reliability and validity tested after adaptation? Was the coping measured with validated instruments?

Our response:

Thank you for this important comment. Reliability coefficients for all study measures were already reported in the manuscript. In the revised version, we have included evidence of construct validity for adapted measurement scales (SWLS in work context, SHS in work context, MBI, PPQ, and PMS).

Regarding the adaptation of the Satisfaction With Life Scale (SWLS) to the work context, reliability and construct validity analyses were conducted using the present study data, and the results have now been added to the manuscript.

Coping was measured using the Pearlin Mastery Scale, a validated instrument assessing individuals' perceived personal coping and sense of control over life circumstances. The scale was adapted into Indonesian through a translation and expert review process to support content validity. In the revised manuscript, we have added a description of this adaptation process and included evidence of construct validity from the current study.

The study also assessed relational coping and self-care coping. However, these constructs were measured using two self-report items rather than standardized psychometric instruments.

Changes made in text:

Now we write:

Construct validity for SWLS: (Page 14 lines 245-246)

In the present study, CFA supported the expected one-factor structure (CFI = .99, TLI = .97, GFI = .98, RMSEA = .11, SRMR = .02).

Construct validity for SHS: (Pages 14-15 lines 255-257)

In the present study, CFA supported the expected one-factor structure (CFI = .99, TLI = .99, GFI = .99, RMSEA = .04, SRMR = .01).

Construct validity for MBI: (Page 16 lines 291-292)

In the present study, CFA supported the expected one-factor structure (CFI = .98, TLI = .97, GFI = .98, RMSEA = .08, SRMR = .03).

Construct validity for PPQ: (Page 17 lines 304-305)

In the present study, CFA supported the expected one-factor structure (CFI = .98, TLI = .96, GFI = .98, RMSEA = .08, SRMR = .03).

Construct validity for PMS: (Page 18 lines 329-330)

In the present study, CFA supported the expected one-factor structure (CFI = .98, TLI = .96, GFI = .98, RMSEA = .06, SRMR = .03).

SWLS: (Pages 14 lines 239-242)

Work satisfaction was measured using the Satisfaction With Work Scale (SWWS) [51], a work-context version of the Satisfaction With Life Scale (SWLS) [52–54]. It is a 5-item scale measuring cognitive evaluations of one’s work life satisfaction (e.g., “In most ways my life is close to my ideal”).

PMS: (Pages 17-18 lines 323-327)

Perceived control over life circumstances was measured using the Pearlin Mastery Scale [70], a 7-item scale measure of perceived personal coping and mastery over life circumstances (e.g., “I have little control over the things that happen to me”). The scale was translated and adapted into Indonesian through a forward–backward translation procedure, followed by expert review and cognitive interviews.

4. Statistical tests results are missing in Table 2. In table 5: Many predictors shown but no coefficient for many variables, for example- marital status, rank, university type, income.

Our response:

We acknowledge that the original version of Table 2 did not include the complete statistical test results. In response to this comment, we have provided the full statistical test results, including the relevant test statistics and exact p-values, in the Supplementary Material. To maintain the readability of the main manuscript and avoid an overly dense table, Table 2 has been retained as a concise summary of the key findings.

In the previous version of the manuscript, we reported only the statistically significant coefficients in Table 5. Following the reviewer's suggestion, we have revised Table 5 to include the coefficients for all predictor variables.

Changes made in text:

See Table 2 (Pages 22-23 lines 428-432), Table 5 (Pages 31-33 lines 545-573), and S3 Table.

5. The manuscript states: Older academics reported lower distress (β = .15). Seems to be inappropriate interpretation as positive beta indicates increase, not decrease.

Our response:

Thank you for this careful observation. The negative sign (-) in the beta coefficient was inadvertently omitted in the manuscript text and should have been consistent with the value reported in Table 5. We have corrected the text to accurately reflect the negative association between age and psychological distress.

Changes made in text:

Now we write: (Pages 30 lines 532-534)

Older academics reported lower psychological distress, including reduced stress (β = -.15, p < .001), anxiety (β = -.12, p < .001), depression (β = -.10, p < .001), and emotional exhaustion (β = -.16, p < .001).

6. Please add attrition analysis.

Our response:

Thank you for this suggestion. We have conducted an attrition analysis comparing participants who completed both waves with those who did not complete the follow-up assessment. The results have been added to the manuscript, with the full details provided in the Supplementary Materials.

Changes made in the text: (Page 18-19 lines 346-352)

An attrition analysis was conducted by comparing completers and non-completers across demographic characteristics using Pearson's chi-square tests. Significant differences were observed for institution type, χ²(3) = 9.04, p = .029, and geographic location, χ²(1) = 5.06, p = .025. No significant differences were found for gender, age group, marital and parental status, academic rank, income, or secondary (non-academic) employment category (all ps > .05). Detailed attrition analysis results are provided in S2 Table.

Reviewer #2

A. Abstract

1. The abstract contains an internal contradiction that must be resolved before publication. The problem statement asserts that mental health challenges in this population have risen sharply, driven by systemic pressures. Yet the results section of the abstract reports that severe psychiatric symptoms were uncommon, with high stress affecting only 2.0%, severe anxiety 3.2%, and severe or very severe depression 3.9% of participants. These two framings are in tension and may mislead readers. The authors should either: (a) revise the problem statement to acknowledge that elevated subclinical distress is prevalent even when severe symptoms are rare, and that mild-to-moderate distress still carries significant individual and institutional costs; or (b) revise the framing in line with the actual findings, noting that while systemic pressures are real and well-documented internationally, Indonesian academics in this sample reported broadly mild-to-moderate distress with a small minority experiencing severe symptoms.

Our response:

Thank you for this insightful comment and for providing helpful alternatives. We adopted option (a) and revised the abstract to provide a more nuanced framing of mental health challenges among academics. Specifically, we now acknowledge that systemic pressures have only partially contributed to these challenges and emphasize that the levels of psychological distress observed in our sample were generally mild to moderate.

Changes made in text:

(Page 3 lines 28-30)

However, mental-health challenges in this population have risen in recent years, partially driven by systemic pressures such as hypercompetition, heavy workloads, job insecurity, and escalating expectations for research productivity and publication output.

(Page 3 lines 35-37)

Most participants reported low/minimal anxiety (63.1%) and moderate stress (63.8%), while depression was mostly low/minimal (43.3%) or mild (36.3%).

2. Additionally, the abstract should include at minimum one sentence contextualising the key regression findings quantitatively (e.g., the effect sizes for personal mastery and publication stress), so that readers can appreciate the magnitude of associations, not just their direction.

Our response:

Thank you for this valuable suggestion. We have revised the abstract to include quantitative information on the key regression findings by reporting the effect sizes (β coefficients).

Changes made in text:

Now we added: (Page 3 lines 39-45)

Across the risk and protective factors examined, higher publication stress and greater experience of unwanted behavior at the workplace were consistently associated with lower levels of well-being and higher psychological distress (β = -.19 to .20 and -.15 to .16, respectively; all ps < .001). In contrast, higher personal mastery was consistently associated with better mental-health (β = .39), greater work satisfaction (β = .33), higher happiness at work (β = .37), and lower stress (β = -.50), anxiety (β = -.40), depression (β = -.41), and emotional exhaustion (β = -.31) (all ps < .001).

B. Introduction

I. Conceptual framing

The introduction frames publication pressure and performance-based evaluation systems as inherently damaging to academic mental health. This position is too one-sided and risks alienating readers who work within or design such systems. The authors should acknowledge that the relationship between performance expectations and well-being is conditional: when institutions provide adequate support, mentorship, and resources, high expectations need not be detrimental. The discussion of Indonesia-specific pressures (lines 63–73) is valuable and should be retained, but qualified accordingly.

Our response:

Thank you for this suggestion. We revised the Introduction to provide a more balanced discussion of performance expectations and academic well-being.

Changes made in text:

We revised the relevant paragraph as follows:

Previous version:

"It is stated that higher education institutions’ quality is benchmarked through international ranking systems, which eventually leads to academic productivity being measured in a major part through their research and publications. Such pressures are consistently associated with adverse outcomes, including emotional exhaustion, reduced job satisfaction, and increased turnover intentions [2,11]."

Revised version: (Pages 5-6 lines 74-80)

" It is stated that higher education institutions’ quality is benchmarked through international ranking systems, which has lead academic productivity to be evaluated largely through research outputs and publications. While such expectations may foster academic achievement and institutional development, they may also contribute to emotional exhaustion, reduced job satisfaction [2,11], and increased turnover intentions [12], particularly when accompanied by limited institutional support, mentoring, or resources."

II. Specific line comments

1. Lines 118–119: The sentence regarding plagiarism cases involving university rectors is out of place in a section discussing systemic pressures on academic mental health. If the authors wish to illustrate the consequences of publication pressure on research integrity, a more direct link to the mental health implications (e.g., the psychological burden of being pressured into unethical conduct) should be made explicit, or the example should be removed.

Our response:

Thank you for this observation. We agree that this example was not directly relevant to the focus of the present study and have therefore removed it.

Changes made in the text: (Page 8 lines 136)

The sentence, "A notable example includes plagiarism cases involving university rectors who were declared not guilty despite substantial evidence," was removed from the manuscript.

2. Lines 131–132: The phrase “empirical studies focusing on the mental health of academics in Indonesia are remain nonexistent” is grammatically incorrect. Please revise throughout. Given that the claim of total nonexistence is strong, the authors should confirm through a current literature search that no relevant empirical work has been published.

Our response:

Thank you for pointing this out. We revised the sentence to correct the grammatical error and to avoid an overly strong claim. Following an updated literature review, we found that empirical studies on the mental health of Indonesian academics remain limited. The text has been revised accordingly.

Changes made in the text: (Page 9 line 154-155)

“In contrast to the extensive body of international research, empirical studies focusing on the mental health of academics in Indonesia are remain limited.”

3. Lines 134–135: “Below are 4 questions we would like to address” should be written in the past tense and revised to academic register. Suggested revision: “This study addressed four research questions:”

Our response:

Thank you for this suggestion. We revised the sentence to follow an appropriate academic style and consistent use of tense.

Changes made in the text: (Page 9 line 157)

“This study addressed four research questions:”

4. Lines 139–142: Research questions 3 and 4 are framed using the causal language “associate with” and “buffer against”. These imply directionality that cannot be established with a cross-sectional design. Revise to explicitly correlational language: e.g., “To what extent are risk factors such as workload and publication pressure associated with mental health outcomes?”

Our response:

Thank you for this comment. We agree that the original wording could imply causal interpretations that are not supported by a cross-sectional design. We have revised the language throughout the manuscript, replacing causal or directional terms with correlational wording

Changes made in text:

Now we write: (Page 9 lines 161-164)

To what extent are risk factors such as workload, publication pressure, and experiences of unwanted behavior at the workplace associated with mental health outcomes?

To what extent are protective factors such as coping strategies associated with mental health in this population?

C. Materials and Methods

Design

This subsection correc

Attachment Submitted filename: Response to Reviewers.docx

# Peer review: Decision Letter 1

**DOI:** 10.1371/journal.pone.0358652.r003

**Authors:** Kristiawan Indriyanto

25 Aug 2026

Dear Dr. Abidin,

Please submit your revised manuscript by Oct 09 2026 11:59PM. If you will need more time than this to complete your revisions, please reply to this message or contact the journal office at plosone@plos.org. When you're ready to submit your revision, log on to https://www.editorialmanager.com/pone/ and select the 'Submissions Needing Revision' folder to locate your manuscript file.

- A letter that responds to each point raised by the academic editor and reviewer(s). You should upload this letter as a separate file labeled 'Response to Reviewers'.
- A marked-up copy of your manuscript that highlights changes made to the original version. You should upload this as a separate file labeled 'Revised Manuscript with Track Changes'.
- An unmarked version of your revised paper without tracked changes. You should upload this as a separate file labeled 'Manuscript'.

If you would like to make changes to your financial disclosure, please include your updated statement in your cover letter. Guidelines for resubmitting your figure files are available below the reviewer comments at the end of this letter.

If applicable, we recommend that you deposit your laboratory protocols in protocols.io to enhance the reproducibility of your results. Protocols.io assigns your protocol its own identifier (DOI) so that it can be cited independently in the future. For instructions see: https://journals.plos.org/plosone/s/submission-guidelines#loc-laboratory-protocols. Additionally, PLOS One offers an option for publishing peer-reviewed Lab Protocol articles, which describe protocols hosted on protocols.io. Read more information on sharing protocols at https://plos.org/protocols?utm_medium=editorial-email&utm_source=authorletters&utm_campaign=protocols.

As the corresponding author, your ORCID iD is verified in the submission system and will appear in the published article. PLOS supports the use of ORCID, and we encourage all coauthors to register for an ORCID iD and use it as well. Please encourage your coauthors to verify their ORCID iD within the submission system before final acceptance, as unverified ORCID iDs will not appear in the published article. *Only*  the individual author can complete the verification step; PLOS staff *cannot*  verify ORCID iDs on behalf of authors.

We look forward to receiving your revised manuscript.

Kind regards,

Kristiawan Indriyanto

Academic Editor

PLOS One

Journal Requirements:

If the reviewer comments include a recommendation to cite specific previously published works, please review and evaluate these publications to determine whether they are relevant and should be cited. There is no requirement to cite these works unless the editor has indicated otherwise.

Please review your reference list to ensure that it is complete and correct. If you have cited papers that have been retracted, please include the rationale for doing so in the manuscript text, or remove these references and replace them with relevant current references. Any changes to the reference list should be mentioned in the rebuttal letter that accompanies your revised manuscript. If you need to cite a retracted article, indicate the article’s retracted status in the References list and also include a citation and full reference for the retraction notice.

[Note: HTML markup is below. Please do not edit.]

Reviewers' comments:

Reviewer's Responses to Questions

**Comments to the Author**

Reviewer #1: All comments have been addressed

Reviewer #3: All comments have been addressed

**********

2. Is the manuscript technically sound, and do the data support the conclusions?

Reviewer #1: Yes

Reviewer #3: Yes

**********

3. Has the statistical analysis been performed appropriately and rigorously?

Reviewer #1: I Don't Know

Reviewer #3: Yes

**********

4. Have the authors made all data underlying the findings in their manuscript fully available?

The PLOS Data policy

Reviewer #1: Yes

Reviewer #3: Yes

**********

5. Is the manuscript presented in an intelligible fashion and written in standard English?

Reviewer #1: Yes

Reviewer #3: Yes

**********

Reviewer #1: All the comments are addressed. In my opinion, the manuscript is acceptable. The manuscript content is consistently mentioned across the sections. The conclusion reflects the study's aims and provides relevant implications based on the results. and language is appropriate.

Reviewer #3: Secon revision of the paper titled Mental health in academia: A national survey on risk and protective factors in the Indonesian context

Dear Authors

I carefully read the revised paper and your detailed point by point replies to my comments. All 13 points raised by my review have been addressed and the quality of paper has substantially improved. Almost all points do not need further refinement, but some of them (n. 2 and 12) need, in my view, minor revisions

Specifically,

Point 1 “The introduction could be improved by better highlighting the reason because mental health of academics needs attention and what would be the key-points for this, even by stressing the link between academic performance and academics’ mental health. It would be useful also to have a more in-depth description of the variables of the previous studies on the same topic cited (i.e. gender, age, status as possible risk and/or risk factors” has been fully addressed both in text and in the response letter

Point 2 concerning the part related to “It is expected to have the number and ratio of participants who accepted the invitation to collaborate in the survey already here and a mention on missing data treatment. It is not clear how many participants accepted and how many were invited” has been well addressed by adding essential information on participants flow. Nevertheless, from your accurate description it appears that only a minority of the whole eligible group of participants accepted to take part in the survey. This needs to be fully discussed in the conclusion and limitation section, because is part of the ecological validity of your study. I understand that the issue of completers vs non completers and the attrition analysis has been fully addressed, but it needs to be clear to the reader that only a part of the academic faculty community accepted to participate, possibly with a mention of similar studies that reported this data.

Point 3 Description of measures has been successfully improved.

Point 4 The inclusion of a summary table for instruments and their description would be a helpful contribution of the clarity of your paper

Point 5 The inclusion of a further CFA analysis, as suggested, fully clarified and completed the psychometric properties of all adapted or modified measures used in your study

Point 6 Missing data and attrition rate analysis for comparing completers and non completers added to your paper, fully contributed to reliability of your data

Point 7 The completion of information on table 2 results fully addressed my point

Point 8 The completion of this information by adding the model building strategy for regression analyses contributed in clarifying this aspect

Point 9 The specification of the coding of the categorical predictors in the regression analyses are now clear

Point 10 The accepted suggestion to assess multicollinearity contributed in full reporting regression analyses results

Point 11 table 5 is now reporting in a correct and complete way and allows the reader to properly catch the information on mental health levels and its correspondence with the severity categories reported for each measure in the Measures section

Point 12 page 40-41 on discussion, lines 748-771 needs, in my view, to be reworded in a more nuanced and cautious way, given that the sample inquired on all variables is only a small part of the whole eligible academic faculty population. This point is a strength and, at the same time, a limitation of your study. You reached a large sample which is, nevertheless, only a part of the whole population.

Point 13 the reduction of causal language improve the reliability of your conclusion.

To sum up my final comments, I found the paper significantly improved, with main points raised in the revision process accurately addressed by the Authors. Only 2 points need a further refinement as minor revisions.

**********

what does this mean?). If published, this will include your full peer review and any attached files.

If you choose “no”, your identity will remain anonymous but your review may still be made public.

**Do you want your identity to be public for this peer review?**  For information about this choice, including consent withdrawal, please see our Privacy Policy

Reviewer #1: No

Reviewer #3: **Yes:** Lavinia Barone University of Pavia, IT

**********

[NOTE: If reviewer comments were submitted as an attachment file, they will be attached to this email and accessible via the submission site. Please log into your account, locate the manuscript record, and check for the action link "View Attachments". If this link does not appear, there are no attachment files.]

To ensure your figures meet our technical requirements, please review our figure guidelines: https://journals.plos.org/plosone/s/figures

You may also use PLOS’s free figure tool, NAAS, to help you prepare publication quality figures: https://journals.plos.org/plosone/s/figures#loc-tools-for-figure-preparation.

NAAS will assess whether your figures meet our technical requirements by comparing each figure against our figure specifications.

Attachment Submitted filename: Secon revision of the paper titled Mental health in academia.pdf

# Author comment: Author response to Decision Letter 2

**DOI:** 10.1371/journal.pone.0358652.r004

28 Aug 2026

Submission

Mental health in academia: A national survey on risk and protective factors in the Indonesian context

Dear reviewer,

First, we would like to thank you for your constructive input and for recognizing our effort to improve this paper. Regarding your feedback, below we have written down your comments, which we put in italics. Your comments are followed by our response and the associated changes made in the text (the line information refers to the "Manuscript" file). We hope you will appreciate the new version, and we look forward to finalizing this draft and preparing it for publication.

Reviewer #3

1. Point 2 concerning the part related to “It is expected to have the number and ratio of participants who accepted the invitation to collaborate in the survey already here and a mention on missing data treatment. It is not clear how many participants accepted and how many were invited” has been well addressed by adding essential information on participants flow. Nevertheless, from your accurate description it appears that only a minority of the whole eligible group of participants accepted to take part in the survey.

This needs to be fully discussed in the conclusion and limitation section, because is part of the ecological validity of your study. I understand that the issue of completers vs non completers and the attrition analysis has been fully addressed, but it needs to be clear to the reader that only a part of the academic faculty community accepted to participate, possibly with a mention of similar studies that reported this data.

Our response:

Thank you for this important comment. We have now added this point to the Limitations section.

Changes made in the text:

In addition, only a minority of the broader eligible academic community participated in the survey, which may limit the representativeness of the sample. Similar challenges with participation rates have also been reported in large-scale surveys of academic populations [105]. (page 40, lines 745-748)

2. Point 12 page 40-41 on discussion, lines 748-771 needs, in my view, to be reworded in a more nuanced and cautious way, given that the sample inquired on all variables is only a small part of the whole eligible academic faculty population. This point is a strength and, at the same time, a limitation of your study. You reached a large sample which is, nevertheless, only a part of the whole population.

Our response:

Thank you for this important comment. We agree that the conclusion should be phrased more cautiously to reflect that, although the study included a large national sample, it represents only a proportion of the eligible academic population. We have therefore revised the wording to avoid overgeneralization.

Changes made in the text:

We changes this sentence:

In conclusion, this national survey reveals that Indonesian academics generally reported mild to moderate levels of stress and anxiety, while severe psychiatric symptoms were relatively uncommon. Several groups appeared particularly vulnerable, including women, younger academics, unmarried academics, and those in lower academic ranks and income groups.

To :

In conclusion, this national survey suggests that the academics who participated reported mild to moderate levels of stress and anxiety, while severe psychiatric symptoms were relatively uncommon. Within this sample, women, younger academics, unmarried academics, and those in lower academic ranks and income groups appeared to report greater psychological vulnerability. (page 41, lines 752-755)

Attachment Submitted filename: Response_to_Reviewers_auresp_2.docx

# Peer review: Decision Letter 2

**DOI:** 10.1371/journal.pone.0358652.r005

**Authors:** Kristiawan Indriyanto

3 Sep 2026

Mental health in academia:

A national survey on risk and protective factors in the Indonesian context

PONE-D-25-66508R2

Dear Dr. Abidin,

We’re pleased to inform you that your manuscript has been judged scientifically suitable for publication and will be formally accepted for publication once it meets all outstanding technical requirements.

Within one week, you’ll receive an e-mail detailing the required amendments. When these have been addressed, you’ll receive a formal acceptance letter and your manuscript will be scheduled for publication.

An invoice will be generated when your article is formally accepted. Please note, if your institution has a publishing partnership with PLOS and your article meets the relevant criteria, all or part of your publication costs will be covered. Please make sure your user information is up-to-date by logging into Editorial Manager at Editorial Manager® and clicking the ‘Update My Information' link at the top of the page. For questions related to billing, please contact billing support.

If your institution or institutions have a press office, please notify them about your upcoming paper to help maximize its impact. If they’ll be preparing press materials, please inform our press team as soon as possible -- no later than 48 hours after receiving the formal acceptance. Your manuscript will remain under strict press embargo until 2 pm Eastern Time on the date of publication. For more information, please contact onepress@plos.org.

Kind regards,

Kristiawan Indriyanto

Academic Editor

PLOS One

Additional Editor Comments (optional):

Reviewers' comments:

Reviewer's Responses to Questions

**Comments to the Author**

Reviewer #3: All comments have been addressed

**********

2. Is the manuscript technically sound, and do the data support the conclusions?

Reviewer #3: Yes

**********

3. Has the statistical analysis been performed appropriately and rigorously?

Reviewer #3: Yes

**********

4. Have the authors made all data underlying the findings in their manuscript fully available?

The PLOS Data policy

Reviewer #3: Yes

**********

5. Is the manuscript presented in an intelligible fashion and written in standard English?

Reviewer #3: Yes

**********

Reviewer #3: Dear Authors

even the final revisions to your manuscript fully addressed my last comments. I think that the paper has been substantially improved and it could contribute successfully to this area of research

**********

what does this mean?). If published, this will include your full peer review and any attached files.

If you choose “no”, your identity will remain anonymous but your review may still be made public.

**Do you want your identity to be public for this peer review?**  For information about this choice, including consent withdrawal, please see our Privacy Policy

Reviewer #3: **Yes:** Prof. Lavinia Barone UNiversity of Pavia Italy

**********

# Editor report: Acceptance letter

**DOI:** 10.1371/journal.pone.0358652.r006

**Authors:** Kristiawan Indriyanto

PONE-D-25-66508R2

PLOS One

Dear Dr. Abidin,

I'm pleased to inform you that your manuscript has been deemed suitable for publication in PLOS One. Congratulations! Your manuscript is now being handed over to our production team.

At this stage, our production department will prepare your paper for publication. This includes ensuring the following:

* All references, tables, and figures are properly cited

* All relevant supporting information is included in the manuscript submission,

* There are no issues that prevent the paper from being properly typeset

You will receive further instructions from the production team, including instructions on how to review your proof when it is ready. Please keep in mind that we are working through a large volume of accepted articles, so please give us a few days to review your paper and let you know the next and final steps.

Lastly, if your institution or institutions have a press office, please let them know about your upcoming paper now to help maximize its impact. If they'll be preparing press materials, please inform our press team within the next 48 hours. Your manuscript will remain under strict press embargo until 2 pm Eastern Time on the date of publication. For more information, please contact onepress@plos.org.

You will receive an invoice from PLOS for your publication fee after your manuscript has reached the completed accept phase. If you receive an email requesting payment before acceptance or for any other service, this may be a phishing scheme. Learn how to identify phishing emails and protect your accounts at https://explore.plos.org/phishing.

If we can help with anything else, please email us at customercare@plos.org.

Thank you for submitting your work to PLOS One and supporting open access.

Kind regards,

PLOS One Editorial Office Staff

on behalf of

Dr. Kristiawan Indriyanto

Academic Editor

PLOS One
